# Emerging antihypertensive therapies and cardiovascular, kidney, and metabolic outcomes: a Mendelian randomization study

**DOI:** 10.1093/ehjcvp/pvaf015

**Published:** 2025-02-17

**Authors:** Nhu Ngoc Le, Tran Quoc Bao Tran, John McClure, Dipender Gill, Sandosh Padmanabhan

**Affiliations:** BHF Cardiovascular Research Centre, School of Cardiovascular and Metabolic Health, University of Glasgow, 126 University Place, Glasgow G12 8TA, UK; BHF Cardiovascular Research Centre, School of Cardiovascular and Metabolic Health, University of Glasgow, 126 University Place, Glasgow G12 8TA, UK; BHF Cardiovascular Research Centre, School of Cardiovascular and Metabolic Health, University of Glasgow, 126 University Place, Glasgow G12 8TA, UK; Department of Epidemiology and Biostatistics, School of Public Health, Imperial College London, London W2 1PG, UK; BHF Cardiovascular Research Centre, School of Cardiovascular and Metabolic Health, University of Glasgow, 126 University Place, Glasgow G12 8TA, UK

**Keywords:** Mendelian randomization, Hypertension, Cardiovascular, Renal, PDE5 inhibitors, SGC stimulators

## Abstract

**Aims:**

Emerging antihypertensive drug classes offer new opportunities to manage hypertension; however, their long-term effects on cardiovascular, kidney, and metabolic (CKM) outcomes remain to be elucidated. This study aims to explore the effects of phosphodiesterase type 5 inhibitors (PDE5i), soluble guanylate cyclase stimulators (sGCs), endothelin receptor antagonists (ERAs), and angiotensinogen inhibitors (AGTis) on a range of CKM outcomes.

**Methods and results:**

Mendelian randomization (MR), summary-based MR (SMR), and colocalization analyses were applied to assess the drug effect on coronary artery disease (CAD), myocardial infarction (MI), ischaemic stroke, atrial fibrillation (AF), heart failure (HF), type 2 diabetes (T2D), and chronic kidney disease (CKD). Genetic association and gene expression summary data were obtained from the largest European-ancestry genome-wide association studies (GWAS) and the genotype-tissue expression version 8 for 29 tissues relevant to the outcomes' pathophysiology.

Genetically predicted systolic blood pressure (SBP) reduction was associated with reduced risks of all outcomes. PDE5i was associated with reduced risks of CAD (OR per 10-mmHg decrease in SBP: 0.348[95% confidence interval (CI): 0.199–0.607]) and ischaemic stroke (0.588[0.453–0.763]). sGCs showed protective effects against CAD (0.332[0.236–0.469]), MI (0.238[0.168–0.337]), and CKD (0.55[0.398–0.761]). ERA and AGTi showed protective effects against CAD and ischaemic stroke. SMR and colocalization supported the association of gene expression levels of *GUCY1A3* and *PDE5A* with CAD and MI risk.

**Conclusion:**

Our study highlights the potential of PDE5i, sGCs, ERA, and AGTi in reducing cardiovascular and renal risks. These findings underscore the necessity for targeted clinical trials to validate the efficacy and safety of these therapies.

## Graphical Abstract

**Graphical Abstract:**
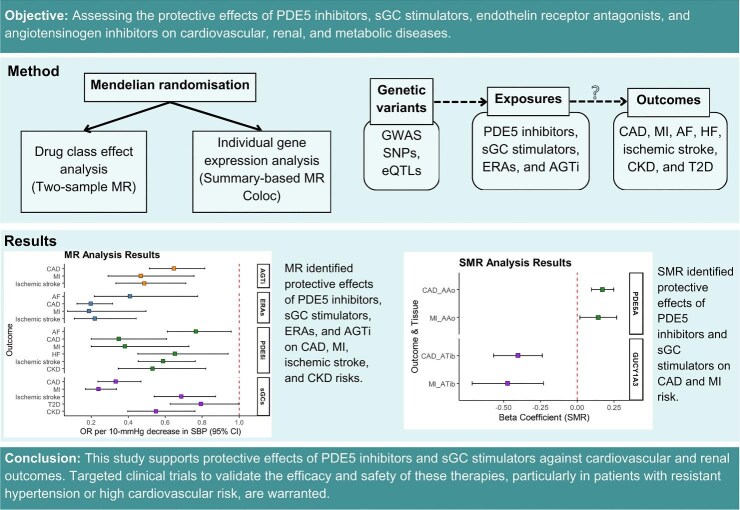
MR, Mendelian randomization; SMR, summary-based Mendelian randomization; SNP, single nucleotide polymorphism; eQTL, expression quantitative trait loci; GWAS, genome-wide association studies; ERAs, endothelin receptor antagonists; AGTi, angiotensinogen inhibitors; CAD, coronary artery disease; MI, myocardial infarction; AF, atrial fibrillation; HF, heart failure; CKD, chronic kidney disease; T2D, type 2 diabetes; SBP, systolic blood pressure; OR, odds ratio; CI, confidence interval; SBP, systolic blood pressure; AAo, artery aorta; ATib, artery tibial.

## Introduction

Hypertension remains a leading modifiable risk factor for cardiovascular morbidity and mortality worldwide, affecting ∼1.3 billion adults globally.^1^ Despite the widespread availability of antihypertensive medications, achieving optimal blood pressure (BP) control continues to be a significant challenge. Approximately 30%–50% of treated hypertensive patients fail to reach target BP levels, highlighting the need for novel therapeutic strategies.^2^

Recent advances in cardiovascular pharmacology have introduced new classes of therapeutic agents targeting alternative pathways implicated in hypertension pathophysiology. Phosphodiesterase type 5 inhibitors (PDE5i) enhance nitric oxide (NO) signalling by preventing cyclic guanosine 3′,5′ -monophosphate (cGMP) degradation, leading to vasodilation. While primarily used for erectile dysfunction and pulmonary arterial hypertension (PAH), PDE5-induced intracellular cGMP stabilization provides potential therapeutic benefits for hypertension, coronary artery disease (CAD), stroke, chronic kidney disease (CKD), and type 2 diabetes (T2D).^3^ Soluble guanylate cyclase stimulators (sGCs) directly stimulate sGC independently of NO availability, augmenting cGMP production, and promoting vasodilation. sGCs have shown efficacy in managing PAH and heart failure (HF) with reduced ejection fraction.^4^ Endothelin receptor antagonists (ERAs) block the effects of endothelin-1, a potent vasoconstrictor implicated in vascular remodelling and hypertension. ERAs have been used to treat PAH, and more recently, aprocitentan has been approved for managing resistant hypertension.^5^ Angiotensinogen inhibitors (AGTis) inhibit the renin-angiotensinogen-aldosterone system (RAAS) at its origin. By reducing angiotensinogen levels, these agents may provide more comprehensive RAAS suppression compared to angiotensin-converting enzyme inhibitors and angiotensin receptor blockers.^6^ Early-phase studies using antisense oligonucleotides targeting angiotensinogen demonstrated promising antihypertensive effects.^7^

Despite the therapeutic potential of these emerging agents, significant gaps in knowledge persist regarding their long-term impact on hypertension and cardiovascular diseases (CVDs). Mendelian randomization (MR) studies offer a valuable tool to assess the causal relationships between therapeutic targets and CVDs.^8^ By leveraging genetic variants as instrumental variables, MR can elucidate the long-term effect of exposure on health outcomes, relatively devoid of confounding factors and reverse causation.^9^ Summary-based MR (SMR) further extends this approach to identify causal effects of gene expression on traits, while colocalization distinguishes between pleiotropy and shared genetic causation.^10,11^ These methods are powerful tools to infer causality and elucidate the mechanisms underlying complex traits.

Our study aims to explore the effects of the four emerging antihypertensive drug classes across a wide range of cardiometabolic and renal outcomes through an integrative approach combining MR, SMR, and colocalization analyses.

## Methods

**Figure 1:**
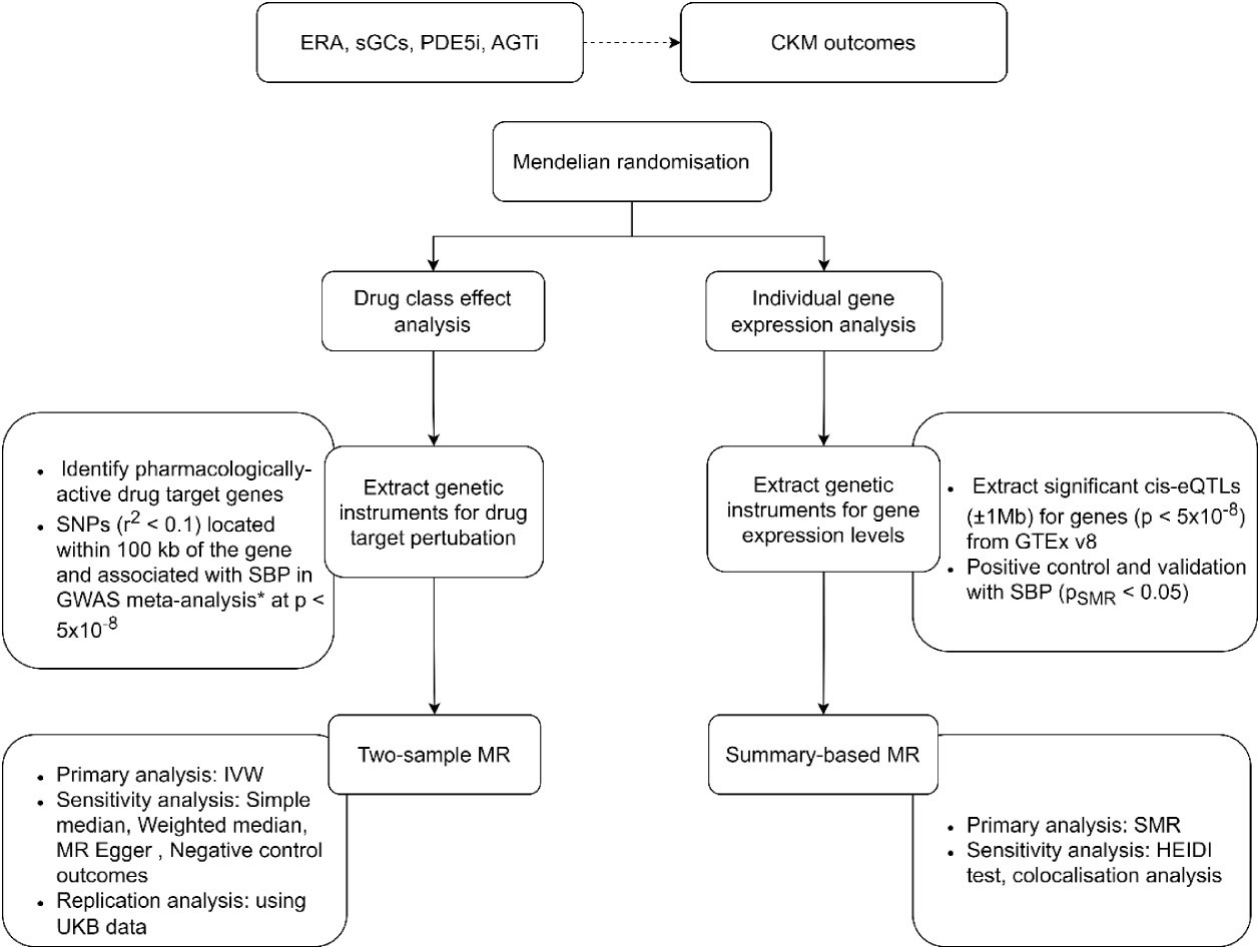
Study design. AGTi, angiotensinogen inhibitor; ERA, endothelin receptor antagonist; sGCs, soluble guanylate cyclase stimulator; PDE5i, phosphodiesterase type 5 inhibitor; SBP, systolic blood pressure; GWAS, genome-wide association study; SNP, single nucleotide polymorphism; eQTL, expression quantitative loci; MR, Mendelian randomization; SMR, summary-based Mendelian randomization; CKM, cardiovascular kidney and metabolic; IVW, inverse-variance weighted; GWAS, genome-wide association studies; (*) indicates a blood pressure GWAS meta-analysis.^12^

Our study includes two stages: (i) perform two-sample MR to explore causal effects of systolic blood pressure (SBP) reduction and emerging antihypertensive drug classes on a range of cardiovascular, kidney, and metabolic (CKM) outcomes; (ii) perform SMR and colocalization to examine if any detected drug-disease association is mediated through gene transcription (*Figure 1*).

All data used in this study are publicly available summary data extracted from genome-wide association studies (GWAS) and expression quantitative trait loci (eQTL) studies, with ethical approval and informed consent from all participants being obtained from their original studies.

The emerging drug classes include ERAs, PDE5is, sGCs, and AGTis. Pharmacologically active protein targets of each drug class and the corresponding genes were identified using the Drugbank database (Supplementary material online, *Table S1*).

### Genetic instruments selection

To proxy for drug class effects, we selected single nucleotide polymorphisms (SNPs) located within 100 kb on either side of each target gene and strongly associated with SBP at a genome-wide significant level in a GWAS meta-analysis^12^ (Supplementary Method). Summary statistics for SBP were obtained from a GWAS meta-analysis of 757 601 European-ancestry participants drawn from the UK Biobank and the International Consortium of Blood Pressure. Linkage disequilibrium (LD) clumping at *r*^2^ < 0.1 was conducted to remove highly correlated SNPs. To proxy for general SBP-lowering effects, we selected SNPs significantly associated with SBP across the whole genome. The *r*^2^ < 0.001 was used for clumping due to a larger number of SNPs. *F*-statistics were used to evaluate instrument strength.^13^

To proxy for the effect of changes in gene expression level, we selected cis-eQTL located within 1Mb window of the gene. The eQTL data were obtained from genotype-tissue expression (GTEx) version 8 (*P* < 5 × 10^−8^)^14^ (Supplementary Method). For positive control testing, only genes whose expression levels were associated with SBP (*P* < 0.05) were taken forward for the SMR analysis.

### Outcome data

The outcomes include CAD, myocardial infarction (MI), ischaemic stroke, atrial fibrillation (AF), HF, T2D, and CKD. These outcomes were selected based on their clinical significance and relevance to hypertension and BP-lowering drug effects.^15–17^ Summary data for these outcomes were obtained from the largest GWAS available, with most participants of European ancestry aligning with our exposure data (*Table 1*).

**Table 1:** GWAS used in this study

**Trait**	**Population**	**Sample size**	**Unit**	**Adjustments**
Systolic blood pressure^12^	European	757 601	mmHg	Age, age^2^, sex, BMI, genotyping chips, antihypertensive medication
Atrial fibrillation^31^	European	1 030 836 (60 620 cases)	log-odds	Age, sex, study-specific covariates, PCs
Heart failure^32^	European	977 323 (47 309 cases)	log-odds	Age, sex, PCs
Coronary artery disease^33^	>95% European	1 165 690 (181 522 cases)	log-odds	Age, sex, study-specific covariates, PCs
Myocardial infarction^27^	77% European	171 875 (43 676 cases)	log-odds	Age, sex, study-specific covariates, PCs
Ischaemic stroke^34^	European	1 296 908 (62 100 cases)	log-odds	Age, sex, study-specific covariates, PCs
Type 2 diabetes^35^	European	933 970 (80 154 cases)	log-odds	Age, sex, study-specific covariates, PCs
Chronic kidney disease^36^	European	480 698 (41 395 cases)	log-odds	Age, sex, study-specific covariates, PCs

BMI, body mass index; PC, principal component.

### Statistical analysis

#### Two-sample MR

MR relies on three main assumptions: (i) the genetic variant is strongly associated with the exposure; (ii) the genetic variant is not associated with any confounders relating to the exposure-outcome; and (iii) the genetic variant is not associated with the outcome, except through the exposure.

The inverse-variance weighted (IVW) method was used as the primary method. A multiplicative random-effects model was used when having more than three genetic variants, otherwise a fixed-effect model was used. MR estimates were presented as odds ratio (OR) for each outcome, with a 95% confidence interval (CI) per 10 mmHg decrease in SBP. A Bonferroni-corrected *P*-value of 0.0014 [0.05/5 (drug classes & SBP) × 7 outcomes] was used to detect significant associations; a *P*-value >0.0014 but <0.05 indicates nominal associations. Data harmonisation was performed. Palindromic variants were not excluded. To ensure consistency in SNPs used across analyses, proxies were not used.

The simple median,^18^ weighted median,^18^ and MR Egger^19^ methods were performed as sensitivity analyses (Supplementary Method). The MR Egger intercept test was performed to detect directional pleiotropy. The effect–effect scatter plots were created to illustrate the effects of individual variants on SBP and the outcomes.

Negative control outcome analysis was performed for the following outcomes: low hand grip strength, myopia, Parkinson's disease, and heel bone mineral density (Supplementary material online, *Table S2*). These outcomes are currently not known to share strong pathological pathways with hypertension or its related CKM outcomes.

As SBP summary data were obtained from a GWAS adjusted for body mass index (BMI), collider bias might be introduced in the MR. We performed a sensitivity analysis using BP summary data from a GWAS that did not adjust for BMI.^20^

To explore whether observed drug class-outcome associations are mediated through their BP-lowering effect, we performed MR analysis to investigate general SBP-lowering effects on the outcomes.

The propagation of error method^21^ was performed to test if MR estimates for drug target perturbation significantly differed from those for general SBP-lowering effects. This allows us to assess whether the observed effects could be attributed solely to SBP reduction or if additional mechanisms may be involved.

LD clumping was performed using the TwoSampleMR package in R^22^; IVW, weighted median, simple median, and MR Egger were performed using the MendelianRandomization package (version 0.6.0) in R.^23^ All analyses were conducted in R software version 4.2.3.

#### Summary-based MR

SMR is a form of MR analysis that integrates GWAS and eQTL summary data to discover a causal association between gene expression level and outcome. Out of 49 tissues in the GTEx v8,^14^ we performed SMR on 29 tissues that are relevant to the pathophysiology of the outcomes and drug mechanisms. SMR estimate was presented as a beta coefficient with 95% CI per 1 unit increase in the gene expression in a particular tissue. A Bonferroni-corrected *P*-value of 0.0003 (0.05/147 tests) was used to explore significant associations. A *P*-value >0.0003 but <0.05 indicates a nominal association.

HEIDI (heterogeneity in dependent instruments) test is integrated into the SMR to investigate if an observed gene expression-outcome association is due to a linkage scenario or a shared causal variant^10^ (Supplementary Method). A *P*_HEIDI_ < 0.01 indicates the association is likely due to a linkage scenario.^10^ SMR analyses and HEIDI tests were performed using SMR software version 1.3.1. Default settings were used: *P*_eQTL_ < 5 × 10^−8^, MAF > 0.01, and eQTL that are in very high or very low LD with the top associated eQTL (*r*^2^ > 0.9 or *r*^2^ < 0.05) were excluded from the HEIDI test to avoid collinearity.^10^

#### Colocalization analysis

Colocalization analysis was performed to investigate if an observed gene expression-outcome association is due to a shared causal variant (Supplementary Method). We used coloc v5.2.1 R package to perform the analysis. The default settings were used: *P*_1_ = 10^−4^, *P*_2_= 10^−4^, *P*_12_= 10^−5^. A high posterior probability (PP) for the scenario of a shared causal variant (H_4_) indicates a high probability of a shared causal variant.^11^

## Results

### Two-sample MR

A total of 454 uncorrelated SNPs (*r*^2^ < 0.001) were identified as instruments for the general SBP-lowering effect (Supplementary material online, *Table S3*). F-statistics for all SNPs were >10, suggesting a low risk of weak instrument bias. Genetic instruments accounted for 4.7% of the total variance in SBP.

We identified two SNPs for AGTi, one SNP for ERA, five SNPs for PDE5i, and six SNPs for sGCs (Supplementary material online, *Table S4–S5*). *F*-statistics for all SNPs were > 10 (ranging from 32.46 to 148.08).

**Figure 2:**
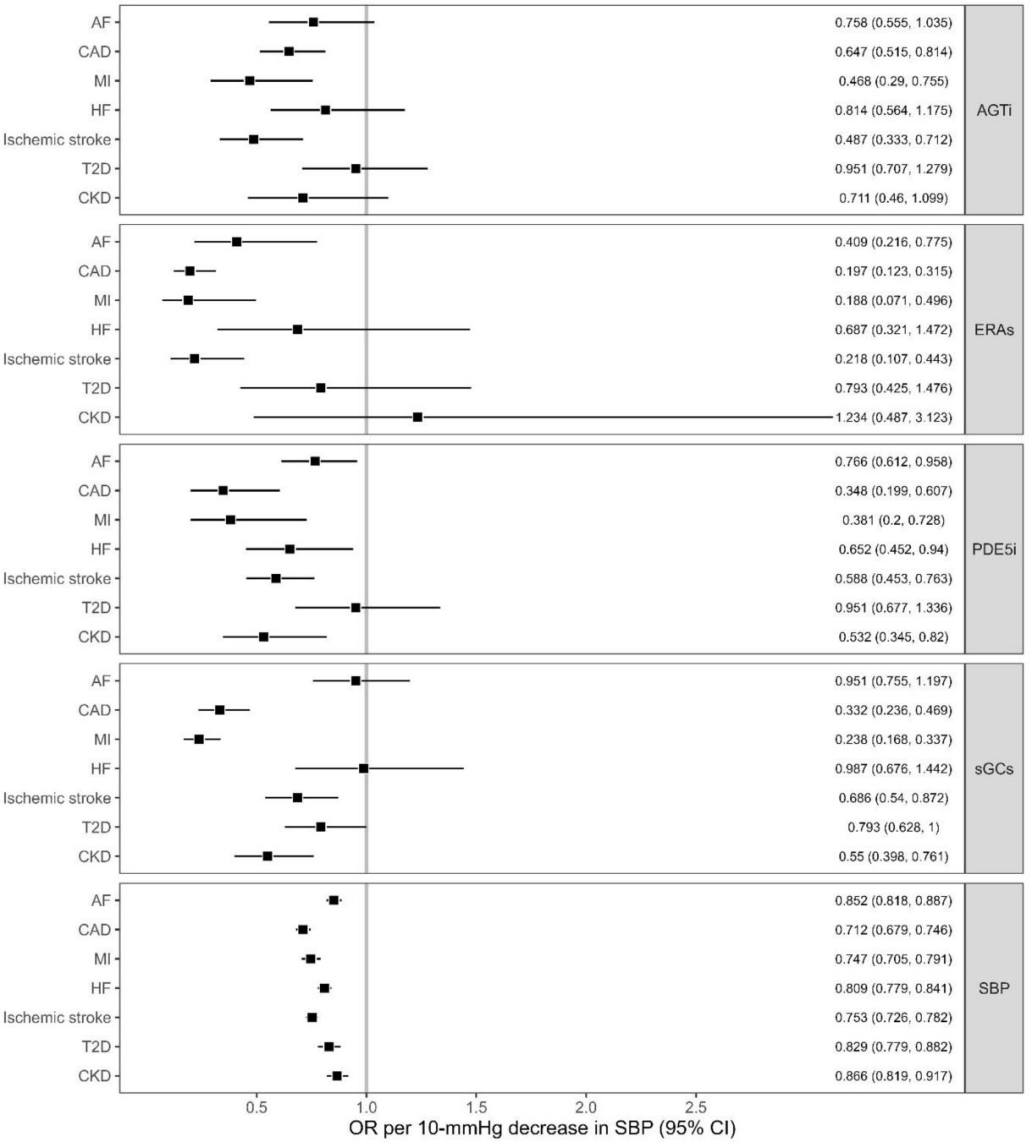
MR estimates for the effect of genetically reduced BP through antihypertensive drug target perturbation on outcomes. Data were represented as OR with 95% CI of the outcome per 10 mmHg decrease in SBP. AGTi, angiotensinogen inhibitor; ERAs, endothelin receptor antagonists; PDE5i, phosphodiesterase type 5 inhibitors; sGCs; soluble guanylate cyclase stimulators; AF, atrial fibrillation; CAD, coronary artery disease; MI myocardial infarction; HF, heart failure; T2D, type 2 diabetes; CKD, chronic kidney disease.

Genetically predicted SBP reduction was significantly associated with a reduced risk of all outcomes (ORs range: 0.71–0.87 per 10 mmHg decrease in SBP) (*Figure 2*, Supplementary material online, *Table S6*). Sensitivity analysis showed consistent results with the IVW for all outcomes except for T2D. The MR Egger intercept test indicated evidence of horizontal pleiotropy in the analysis with T2D (*P*-value intercept test = 0.0004; MR Egger OR, 1.08 (0.92–1.26)) (Supplementary material online, *Table S6*).

Genetically proxied AGTi was significantly associated with a reduced risk of CAD (OR per 10 mmHg decrease in SBP, 0.647, 95% CI, 0.515–0.814, *P* = 0.0002) and ischaemic stroke [0.487 (0.333–0.712), *P* = 0.0002]. There was suggestive evidence for the association with reduced MI risk (*Figure 2*, Supplementary material online, *Tables S7–S8*). The drug effect on ischaemic stroke was significantly larger than the general SBP-lowering effect (*Table 2*).

**Table 2:** Difference between MR estimates for general systolic BP-lowering effects and those for drug target perturbation

**Drug class**	**Exposure**	**Outcome**	*β* **_diff_ (95% CI)**	***P*-value**
AGTi	SBP	CAD	−0.009 (−0.033, 0.014)	0.433
		Ischaemic stroke	−0.044 (−0.082, −0.006)	0.025
ERA	SBP	CAD	−0.128 (−0.175, −0.081)	9.86E−08
		Ischaemic stroke	−0.124 (−0.195, −0.053)	0.001
		MI	−0.138 (−0.234, −0.042)	0.005
PDE5i	SBP	CAD	−0.071 (−0.127, −0.016)	0.012
		Ischaemic stroke	−0.025 (−0.051, 0.001)	0.064
sGCs	SBP	CAD	−0.076 (−0.111, −0.041)	0.00 002
		MI	−0.114 (−0.149, −0.079)	2.16E−10
		CKD	−0.045 (−0.078, −0.012)	0.007

*β*_diff_ indicates a difference between MR estimates for SBP and those for drug target; CI, conference interval; ERA, endothelin receptor antagonist; sGCs, soluble guanylate cyclase stimulator; PDE5i, phosphodiesterase type 5 inhibitor; AGT, angiotensinogen inhibitor; SBP, systolic blood pressure; CAD, coronary artery disease; MI, myocardial infarction; CKD, chronic kidney disease.

Genetically proxied ERA was significantly associated with a reduced risk of CAD (0.197 (0.123–0.315), *P* = 1.19 × 10^−11^), MI (0.188 (0.071–0.496), *P* = 0.0007), and ischaemic stroke [0.218 (0.107–0.443), p = 2.56 × 10^−05^] and nominally associated with a reduced AF risk (*Figure 2*, Supplementary material online, *Tables S7–S8*). The drug effects on CAD, MI, and ischaemic stroke were larger than the general SBP-lowering effects (*Table 2*).

Genetically predicted PDE5i was significantly associated with a lower risk of CAD (0.348 (0.199–0.607), *P* = 0.0002), and ischaemic stroke [0.588 (0.453–0.763), *P* = 6.55 × 10^−05^]. We also found suggestive evidence for the association with a reduced risk of MI, HF, AF, and CKD (*Figure 2*, Supplementary material online, *Table S7*). MR Egger intercept test did not detect evidence of horizontal pleiotropy for the significant associations. Sensitivity analyses consistently support the IVW (Supplementary material online, *Table S7*). The effect–effect scatter plots showed a consistent dose-response pattern (Supplementary material online, *Fig. S1*). The drug effect on CAD risk was significantly larger than the general SBP-lowering effect (*Table 2*).

Genetically proxied sGCs was significantly associated with a reduced risk of CAD (0.332 (0.236–0.469), *P* = 3.45 × 10^−10^), MI (0.238 (0.168–0.337), *P* = 5.68 × 10^−16^), and CKD [0.55 (0.398–0.761], *P* = 0.0003). There were nominal associations with lower ischaemic stroke and T2D risk (*Figure 2*, Supplementary material online, *Table S7*). MR Egger intercept test showed no significant evidence of horizontal pleiotropy. The weighted median and simple median results were consistent with the IVW results. The MR Egger showed consistent effect directions with the IVW for MI and CKD (Supplementary material online, *Table S7*). The effect–effect scatter plots demonstrated a consistent dose-response pattern for most associations (Supplementary material online, *Fig. S2*). For sGCs and CAD, one variant (rs10010626) appeared to deviate from the overall trend. We conducted an additional sensitivity analysis by excluding this SNP. The results from IVW and sensitivity analyses supported the protective effect (Supplementary material online, *Table S9, Fig. S2*). The sGCs drug effects on CAD, MI, and CKD were significantly larger than the general-SBP lowering effects (*Table 2*).

Negative control analyses did not detect any significant association (Supplementary material online, *Table S10*). MR analysis using UKB GWAS for SBP gave consistent results with the primary analyses (Supplementary material online, *Table S11*).

### Summary-based MR

We found eQTLs for the following genes that encode proteins targeted by the drug classes: *AGT* (AGTi), *EDNRA* (ERA), *GUCY1A2, GUCY1A3* (sGCs), and *PDE5A* (PDE5i). The gene expression level of *AGT, GUCY1A2, GUCY1A3*, and *PDE5A* was associated with SBP in at least 1 of the 29 tissues. These genes were taken further for the analysis (Supplementary material online, *Table S12*).

**Figure 3:**
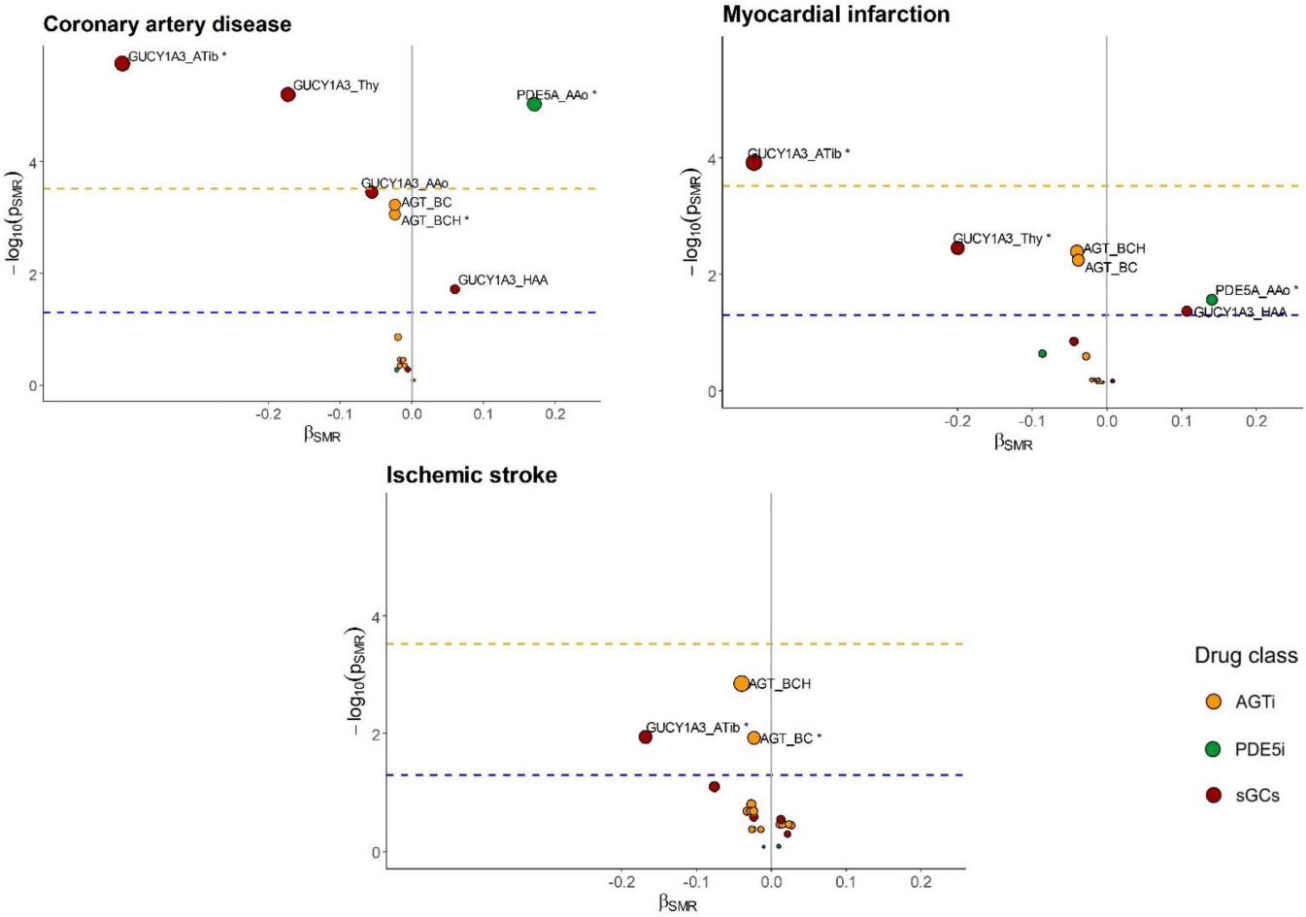
SMR results. The *x*-axis represents the β estimates from SMR analysis, and the *y*-axis represents the −log10(*P*_SMR_). The circle signifies the corresponding gene that encodes the protein targeted by the antihypertensive drug class (PDE5A gene – PDE5 inhibitors, GUCY1A3 gene – sGC stimulators, AGT gene – AGT inhibitors). *β*_SMR_ were estimates from SMR analysis per 1 unit increase in the expression of the genes in a particular tissue. *β*_SMR_ < 0 suggests that an increase in gene expression levels in a particular tissue was associated with a decreased disease risk, and vice versa. The upper horizontal dashed line represents the −log10 of the Bonferroni-corrected *P*-value (0.0003), corresponding to −log10(pSMR) = 3.523, while the lower horizontal dashed line indicates the −log10 of the nominal *P*-value (0.05), equivalent to −log10(pSMR) = 1.301. The asterisk indicates HEIDI *P*> 0.01; AAo, artery aorta; Atib, artery tibial; BCH, brain cerebellar hemisphere; BC, brain cerebellum; Thy, thyroid; HAA, heart atrial appendage; AGTi, angiotensinogen inhibitor; PDE5i, phosphodiesterase type 5 inhibitors; sGCs; soluble guanylate cyclase stimulators.

We observed nominal associations of AGT gene expression with the risk of CAD and AF in brain cerebellar hemisphere, ischaemic stroke in brain cerebellum, and T2D in adipose subcutaneous (Supplementary material online, *Table S13*, *Figure 3*, Supplementary material online, *Fig. S3*).

We detected a significant association of *PDE5A* gene expression level in the aorta with the risk of CAD (per 1-SD increase in gene expression level, *b*_SMR_ = 0.171, *P*_SMR_ = 9.12 × 10^−06^, *P*_HEIDI_ = 0.338), and a nominal association with the risk of MI and T2D (Supplementary material online, *Table S13*, *Figure 3*, *Fig. S3*).

*GUCY1A3* gene expression level in tibial artery was significantly associated with CAD risk (*b*_SMR_ = −0.404, *P*_SMR_ = 1.74 × 10^−06^, *P*_HEIDI_ = 0.663) and MI risk (*b*_SMR_ = −0.473, *P*_SMR_ = 0.0001, *P*_HEIDI_ = 0.873), and was nominally associated with ischaemic stroke and CKD risks. We observed a nominal association with the risk of HF, CKD, and T2D in the aorta, and with MI risk in the thyroid (Supplementary material online, *Table S13*, *Figure 3*, *Fig. S3*).

### Colocalization analysis

**Figure 4:**
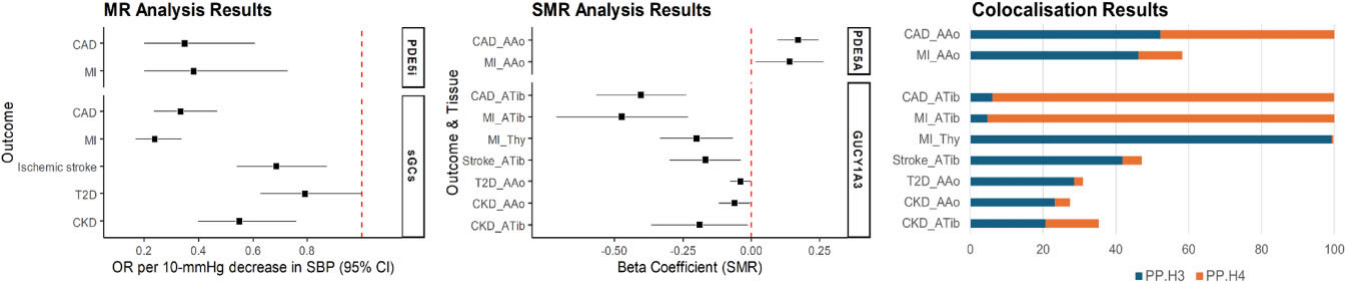
Results from MR, SMR, and colocalization analyses. From left to right, the first graph shows MR results for PDE5 inhibitors (PDE5i) and sGC stimulators (sGCs) with associated outcomes. The second graph shows SMR results for *PDE5A* and *GUCY1A3* and outcomes. The third graph shows the PP (%) for the scenario of distinct causal variants (H3) and of shared causal variant (H4) for the two traits—gene expression level and outcome risk. AAo, artery aorta; Atib, artery tibial; Thy, thyroid. CAD, coronary artery disease; MI, myocardial infarction; CKD, chronic kidney disease; T2D, type 2 diabetes.

Colocalization analysis gave a high PP for a shared causal variant for tibial artery *GUCY1A3* eQTL and CAD risk (H_4_ PP = 93.9%) and for tibial artery *GUCY1A3* eQTL and MI risk (H_4_ PP = 95.3%) (Supplementary material online, *Table S14, Fig. S4*, *Figure 4*). For aorta, *PDE5A*, eQTL, and CAD risk, coloc gave moderate PP for a shared causal variant (H_4_ PP = 47.8%, H_3_ PP = 52.2%).

## Discussion

Our study utilised an integrative approach that combines MR, SMR, and colocalization analyses to investigate the effects of emerging antihypertensive drug classes on a range of cardiometabolic and kidney diseases. We confirm that genetically predicted lower SBP is causally associated with reduced risks of all outcomes, in concordance with evidence from previous studies.^24,25^ We found protective effects of genetically proxied AGTi, ERA, sGCs, and PDE5i for CAD, MI, and ischaemic stroke, and the protective effect of sGCs for CKD. Furthermore, we detected significant associations between the expression levels of genes (*GUCY1A3* and *PDE5A)* and the risk of CAD and MI.

Our analyses demonstrate that the effects of certain drug classes differ significantly from general SBP-lowering effects. For instance, PDE5i showed a more pronounced effect on CAD, and sGCs on CAD, MI, and CKD. While these discrepancies suggest additional cardioprotective pathways, the exact alternative mechanisms remain unknown. Future work should aim to elucidate these mechanisms to further validate our findings.

Our study supports the effectiveness of sGCs in reducing the risks of CAD, MI, and CKD. These findings align with previous research highlighting the role of NO-sGC-cGMP pathway in cardiovascular protection.^26,27^ The *GUCY1A3* gene, which encodes an alpha subunit of the sGC complex, has been linked to CAD and MI in large-scale GWAS.^26,27^ Our results show that increased *GUCY1A3* expression in the tibial artery is associated with lower SBP and a reduced risk of CAD and MI, reinforcing the potential of sGC stimulation as a therapeutic strategy for CAD management.

PDE5i, commonly used for erectile dysfunction and PAH, was also found to have cardioprotective effects.^28^ PDE5i inhibits the breakdown of cGMP, thereby enhancing NO activity, which associated with cardiovascular benefits. Our MR study supports this, showing an association between PDE5i and reduced CAD and ischaemic stroke risks. This was corroborated by SMR findings, which linked increased *PDE5A* expression in the aorta to higher SBP and increased CAD risk. The beneficial effects of PDE5i for CAD and MI were reported in a recent MR study by Xiao et al.^29^ Our findings complement this and provide further insights into the cardioprotective potential of PDE5i and other emerging antihypertensive therapies across a range of CKM outcomes by integrating MR, SMR, and colocalization analyses.

Zilebesiran, an investigational siRNA therapeutic agent, inhibits the hepatic synthesis of angiotensinogen—a precursor protein in the renin-angiotensin system. Treatment with zilebesiran showed promising antihypertensive effects.^7^ Our study supports the protective effect of AGTi for CAD and ischaemic stroke, aligning with the well-established role of RAS in the cardiovascular system.

ERAs block endothelin binding to its receptors, reducing vasoconstriction and aldosterone secretion. ERAs are mainly indicated for PAH; however, recent research suggests the endothelin system's therapeutic potential in other CVDs, including resistant hypertension and microvascular angina.^5^ Our study supports the cardioprotective effect of ERAs for CAD, MI, and ischaemic stroke.

While emerging agents offer potential benefits for hypertension and CVDs, known side effects, such as fluid retention in patients using ERA,^5^ may limit their widespread use, particularly in patients with HF or renal impairment. This highlights the necessity of stratifying patients to optimize benefit-risk profiles. While monitoring and combination therapies may mitigate adverse effects, further research is necessary to elucidate the long-term safety profiles across diverse populations.

A key strength of our study is the comprehensive investigation of emerging antihypertensive drug classes at both the drug class and individual target gene expression levels across a broad spectrum of outcomes. The integration of MR, SMR, and colocalization analyses enhances the credibility of our findings. The convergence of results across different analytical approaches, particularly for PDE5i and sGCs, underscores the robustness of our conclusions.

However, our study has limitations. The summary data used were restricted to individuals of European descent, which may limit the generalizability of our findings. MR estimates reflect the lifetime effect of exposure, which may differ from the effect of clinical interventions initiated later in life. However, MR remains valuable for detecting the existence and directionality of causal effects. As genetic variants may affect other genes or pathways beyond the gene of interest, selecting variants within a 100 kb window of the gene may introduce pleiotropy in the MR analysis. Limited availability of eQTL data for certain tissues may lead to tissue misspecification. Larger eQTL studies with sufficient sample sizes are needed to alleviate this issue. The Bonferroni correction can be overly stringent and may reduce sensitivity; therefore, nominal associations warrant further investigation. The potential overlap between participants in the exposure and outcome GWAS datasets could bias the MR estimates towards observational associations. However, given the strong associations between the genetic instruments and exposures, the impact of such bias is likely minimal.^30^ The GWAS for SBP was adjusted for BMI and medication use, which could introduce bias.^12^ We addressed this by conducting sensitivity analyses using GWAS without these adjustments, yielding consistent results.

## Conclusion

Our findings highlight the cardioprotective potential of emerging antihypertensive therapies, including PDE5 inhibitors, sGC stimulators, and ERAs, across a broad range of cardiovascular and renal outcomes. These results underscore the need for targeted clinical trials to validate the efficacy and safety of these therapies, particularly in patients with resistant hypertension or high cardiovascular risk. Additionally, further research is required to elucidate the mechanisms underlying the observed effects beyond BP lowering, which may inform personalised treatment strategies for hypertension management and CVD prevention.

## Supplementary Material

pvaf015_Supplemental_File
