# Supplementary material for: Emerging antihypertensive therapies and cardiovascular, kidney, and metabolic outcomes: a Mendelian randomization study
Source: Eur Heart J Cardiovasc Pharmacother. 2025 Feb 17;11(3):264–74. doi: 10.1093/ehjcvp/pvaf015 (PMC12046581; doi:10.1093/ehjcvp/pvaf015)
Supplement: pvaf015_Supplemental_File [file pvaf015_supplemental_file.docx]

**SUPPLEMENTARY MATERIALS**

Table of Contents

[Supplementary Method 2](#_Toc190087429)

[Figure S1. The effect-effect scatter plots for the associations of PDE5 inhibition with CAD and ischemic stroke. 5](#_Toc190087430)

[Figure S2. The effect-effect scatter plots for the associations of sGC inhibition with CAD, MI, and CKD. 6](#_Toc190087431)

[Figure S3. SMR associations of antihypertensive target gene-expression levels with heart failure, atrial fibrillation, chronic kidney disease, and type 2 diabetes. 8](#_Toc190087432)

[Figure S4. Colocalisation of eQTL in tibial artery for GUCY1A3 and CAD risk. 10](#_Toc190087433)

[Table S1. Antihypertensive Drug Classes, Drug Names, and Corresponding Drug Target Genes. 11](#_Toc190087434)

[Table S2. GWAS for negative control outcome analysis 12](#_Toc190087435)

[Table S3. Genetic instruments for systolic blood pressure 13](#_Toc190087436)

[Table S4. Genetic proxies identified for each antihypertensive drug class. 21](#_Toc190087437)

[Table S5. Genetic instruments information 22](#_Toc190087438)

[Table S6. MR estimates of the effect of genetically predicted systolic blood pressure on main outcomes. 23](#_Toc190087439)

[Table S7. MR analysis results for genetically proxied drug classes on the main outcomes. 26](#_Toc190087440)

[Table S8. Effects of genetic instruments on systolic blood pressure and the significant outcomes. 32](#_Toc190087441)

[Table S9. Sensitivity analysis for sGC stimulation and coronary artery disease risk after removing a SNP 36](#_Toc190087442)

[Table S10. MR analyses on negative control outcomes, using inverse-variant weighted method. 37](#_Toc190087443)

[Table S11. MR analysis results for genetically proxied drug classes on the main outcome, using blood pressure estimates from UKB GWAS. 38](#_Toc190087444)

[Table S12. SMR associations of antihypertensive target gene-expression levels with systolic blood pressure 40](#_Toc190087445)

[Table S13. SMR associations of antihypertensive target gene-expression with CKM outcomes 42](#_Toc190087446)

[Table S14. Colocalisation results. 45](#_Toc190087447)

[Supplementary References 46](#_Toc190087448)

Supplementary Method

**Genetic selection**

Genetic instruments for drug class effects were selected as single nucleotide polymorphisms (SNPs) located within 100 kb on either side of each drug target gene and strongly associated with SBP at a genome-wide significant level p-value < 5×10^-8^ in a GWAS meta-analysis for BP (1). Relying on significant SNPs identified in the largest available SBP GWAS allows for an unbiased and systematic selection process. For some drug classes whose protein targets are encoded by different genes, genetic instruments for each gene were combined as a single set of instruments for the MR analysis (2). Linkage disequilibrium (LD) clumping at r^2^ < 0.1 was conducted to remove highly correlated SNPs that could potentially bias the MR estimates and inflate the variance of the results. F-statistics were calculated to evaluate the strength of individual genetic instruments (3).

SNPs associated with SBP at a p-value < 5×10^-8^ (1) across the whole genome were selected as genetic instruments for general SBP-lowering effects. Due to the large number of SNPs identified, a more stringent threshold r^2^ < 0.001 was used for the clumping to minimise potential bias from LD. The instrument strength was quantified using R^2^ and F-statistics (3).

Cis-expression quantitative loci (cis-eQTL) were selected as genetic instruments to proxy for the effect of changes in gene expression level. Only cis-eQTL located within 1 Mb window of the gene were considered for the selection. Significant cis-eQTL data (p < 5×10^-8^) were obtained from GTEx version 8 (49 tissues, from nearly 1000 deceased individuals, predominantly European ancestry) (4). The eQTL data is on a scale of 1 unit change in gene expression level per each additional effect allele. For positive control testing and validation, only genes whose expression levels were associated with SBP (p < 0.05) were taken forward for the SMR analysis.

**MR sensitivity analyses**

The simple median (5), weighted median (5) and MR Egger (6) methods were performed as sensitivity analyses to evaluate the robustness of MR findings to potential bias. These methods have less stringent assumptions compared to the IVW method. Simple median and weight median methods require at least 50% of the genetic variants to be valid to give a robust MR estimate. MR Egger can give a consistent MR estimate of the causal effect when up to 100% of genetic variants are invalid but require the Instrument Strength Independent of Direct Effect (InSIDE) assumption to be satisfied. The InSIDE assumption states that there is no correlation between the pleiotropic effects of genetic variants and the variant-exposure association. Additionally, the MR Egger method provides a test, known as the MR Egger intercept test, to detect directional pleiotropy. The deviation of the intercept from zero indicates evidence of directional pleiotropy (6). Additionally, the effect-effect scatter plots were created to illustrate the effects of individual variants on SBP and the corresponding outcomes.

**HEIDI test**

HEIDI test is integrated into the SMR method to investigate if an observed association between gene expression and the outcome is due to a linkage scenario or a shared causal variant (7). In the linkage scenario, the SNP that affects gene expression is in linkage disequilibrium with another SNP that independently impacts the outcome. The HEIDI examines whether the estimated effect sizes for multiple SNPs in the cis-eQTL region are heterogeneous, which indicates the linkage scenario. The null hypothesis of HEIDI is that there is no heterogeneity, which indicates there is a shared causal variant for gene expression and the outcome. A p_HEIDI_ of 0.01 was used as a threshold to indicate a linkage scenario - p_HEIDI_ < 0.01 indicates the observed association is likely due to a linkage scenario (7, 8).

**Colocalisation analysis**

Colocalisation analysis was performed to investigate if an observed association between gene expression level and the outcome is due to a shared causal variant. We used coloc v5.2.1 R package to perform the analysis. Within a particular genomic region, a Bayesian test was performed to calculate posterior probabilities (PP) for five scenarios (17):

H_0_: There is no variant associated with either trait.

H_1_ and H_2_: There is a variant associated with only one of the two traits.

H_3_: There are distinct causal variants associated with each of the traits.

H_4_: There is a shared causal variant for both traits.

A high PP for H_4_ indicates a high probability of a shared causal variant for the traits. Whereas a high PP for H_3_ suggests that the observed association is likely due to a linkage scenario. We used default prior settings for the colocalisation analysis: p1 = 10^-4^, p2 = 10^-4^, p12 = 10^-5^.

Figure S1. The effect-effect scatter plots for the associations of PDE5 inhibition with CAD and ischemic stroke.


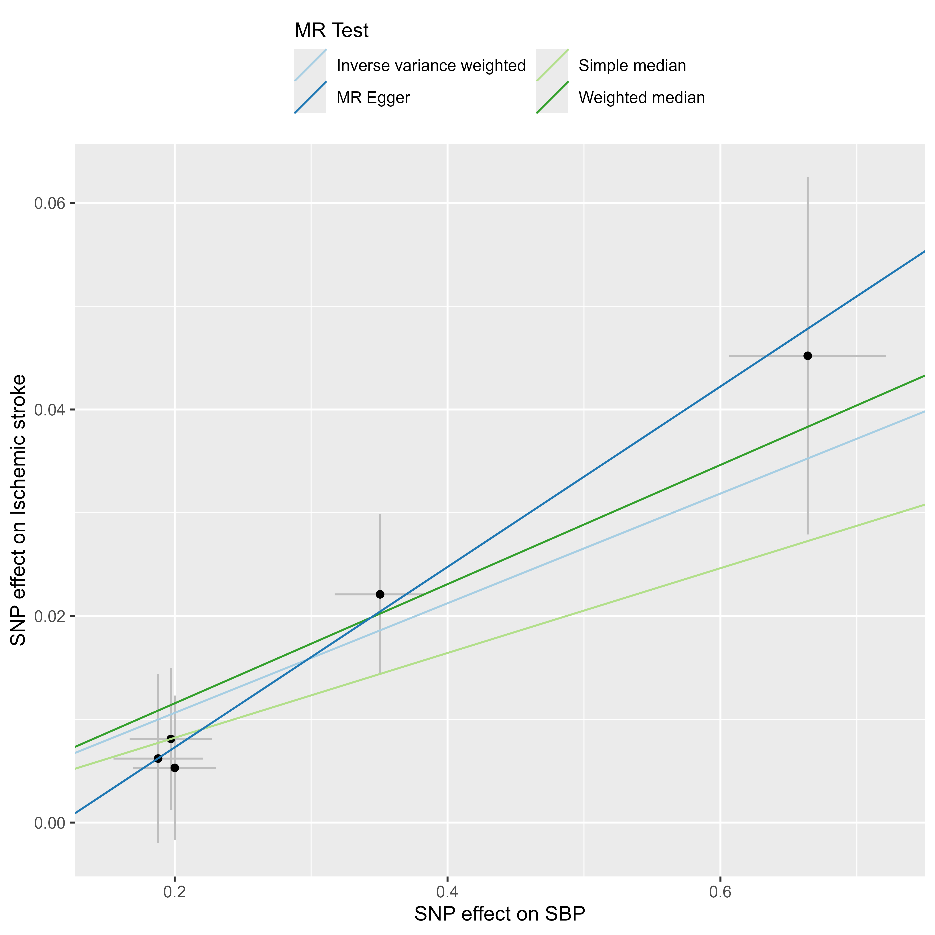


**PDE5i - Stroke**


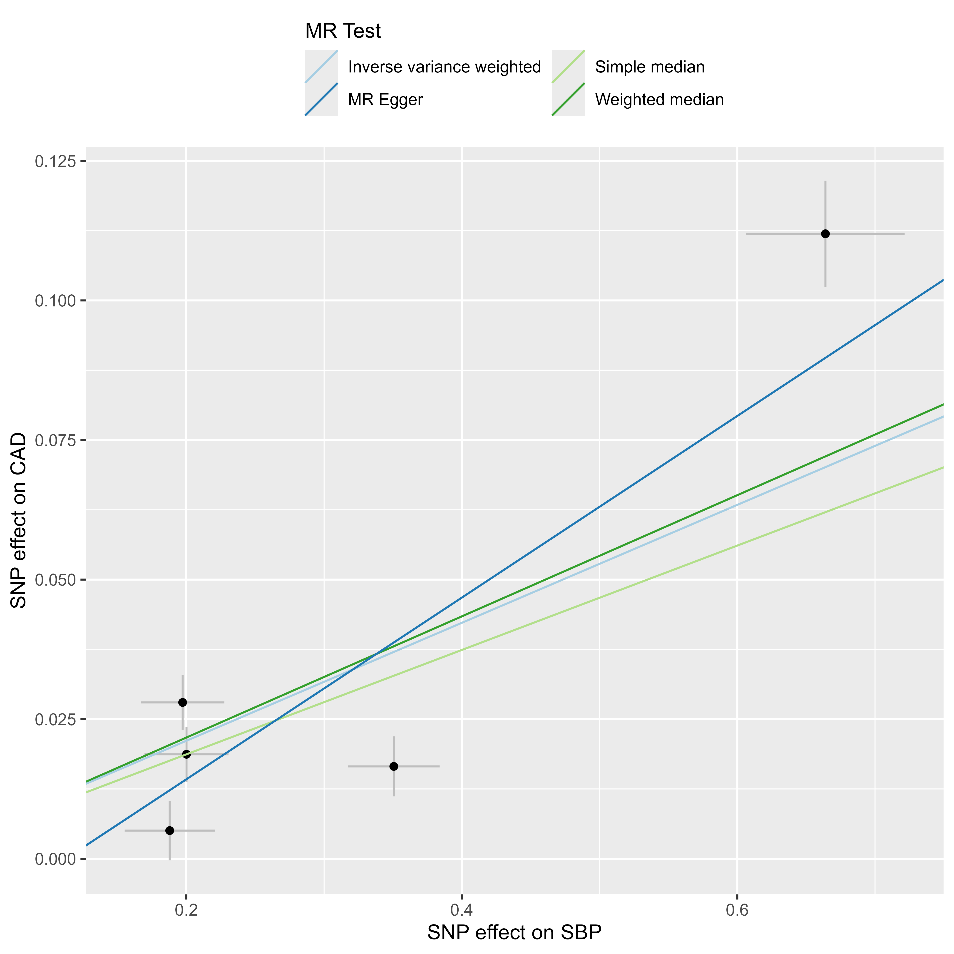


**PDE5i - CAD**

Figure S2. The effect-effect scatter plots for the associations of sGC inhibition with CAD, MI, and CKD.

For CAD, sensitivity analysis by excluding one SNP, rs10010626, is also presented.


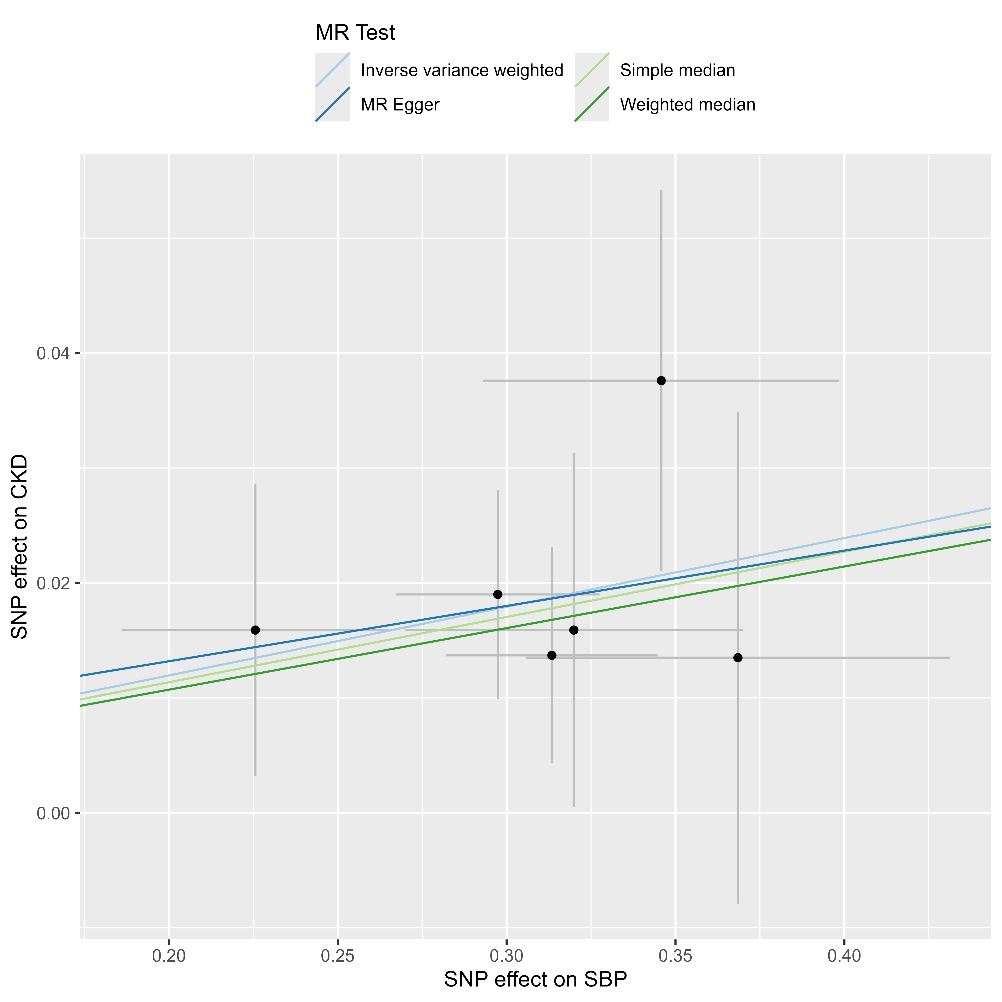


**sGCs - CKD**


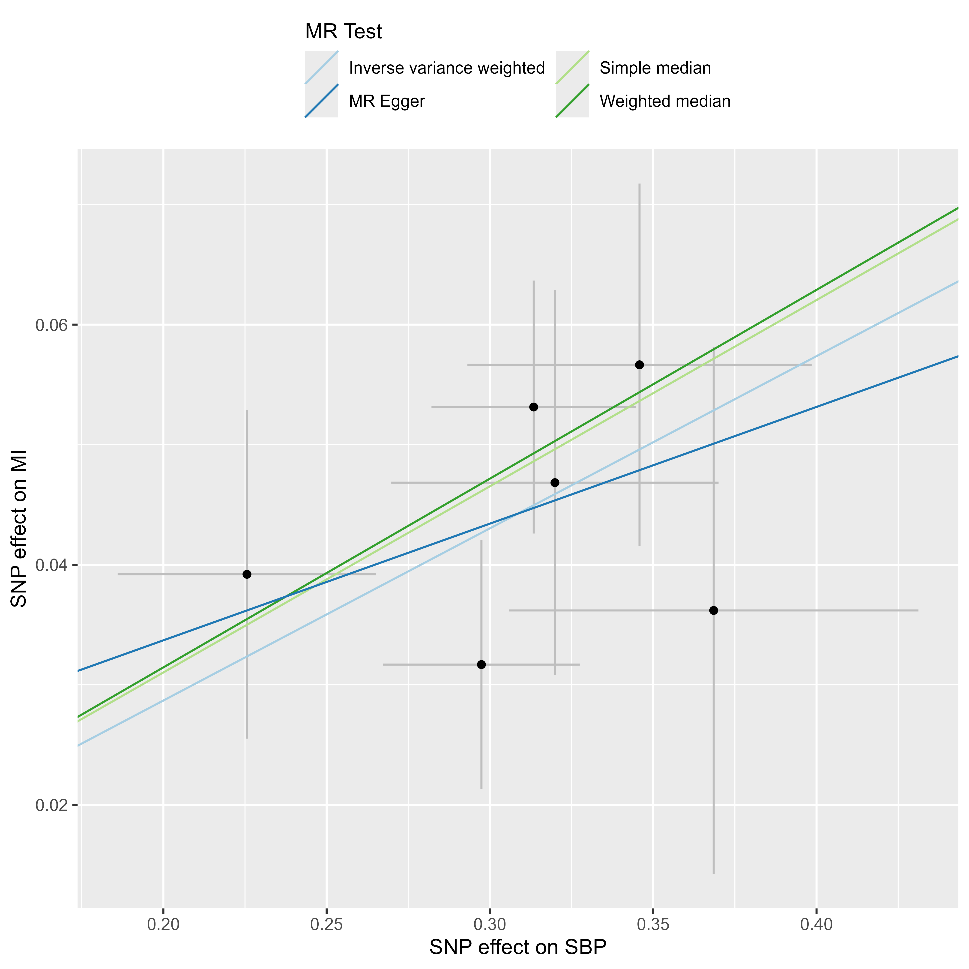


**sGCs - MI**


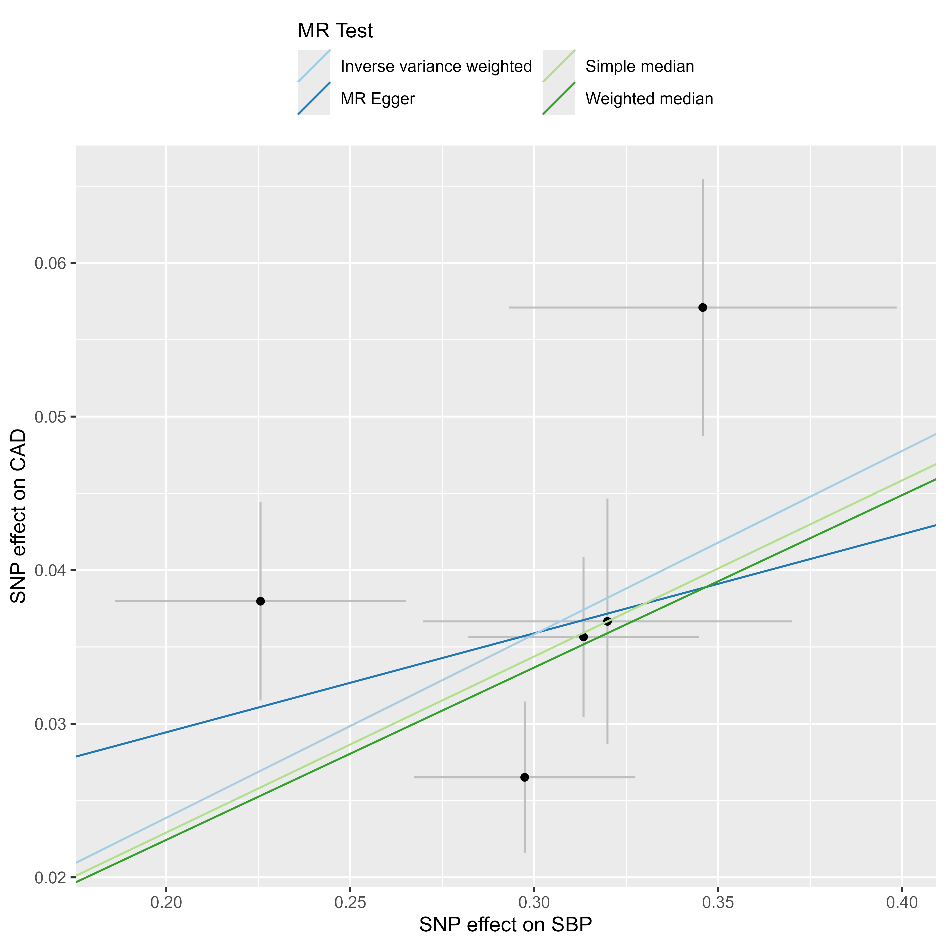


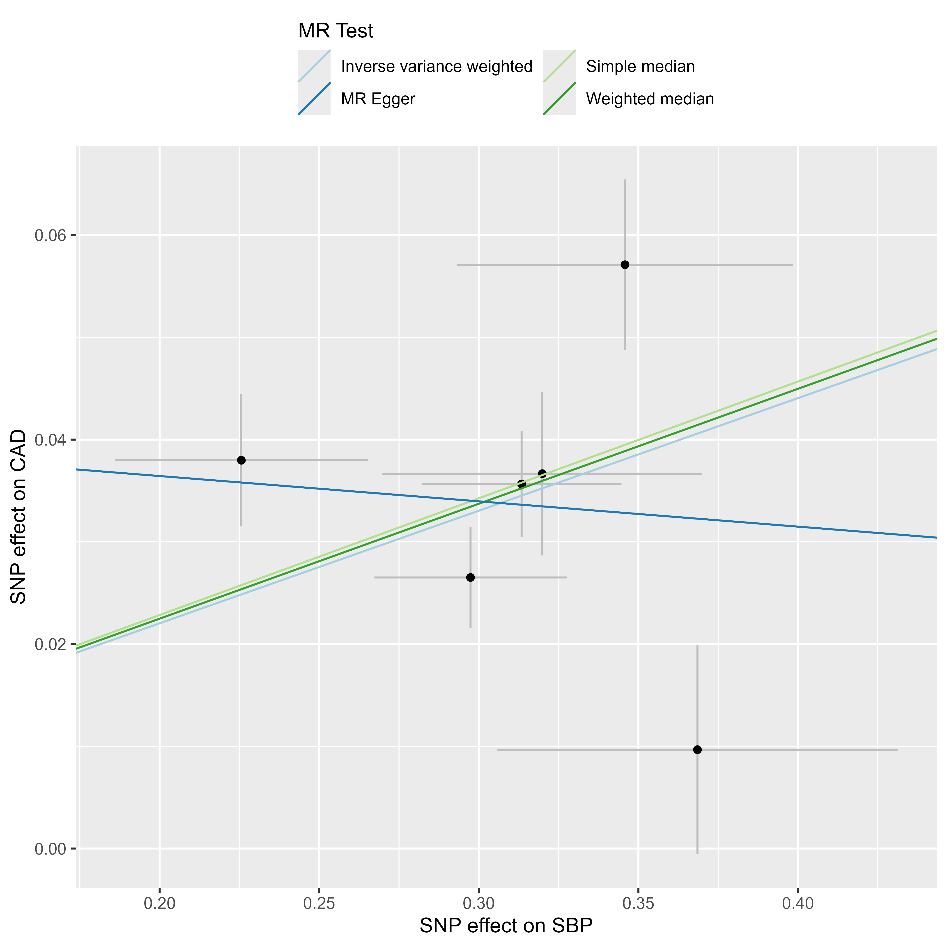


**sGCs - CAD**


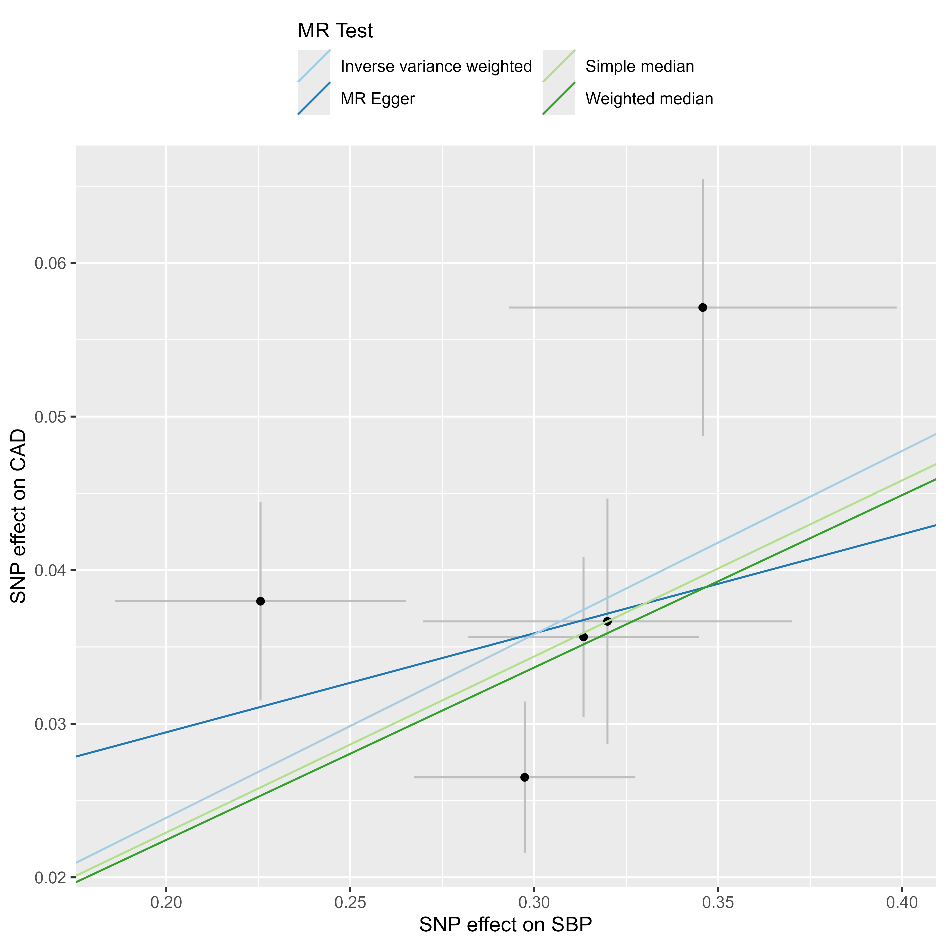


**sGCs - CAD**

remove rs10010626

Figure S3. SMR associations of antihypertensive target gene-expression levels with heart failure, atrial fibrillation, chronic kidney disease, and type 2 diabetes.

The x-axis represents the β estimates from SMR analysis, and the y-axis represents the -log10(p_SMR_). The circle, which signifies the corresponding gene, was coloured according to the drug class that targets its protein. β_SMR_ were estimates from SMR analysis per 1 unit increase in the expression of the genes in a particular tissue. β_SMR_ < 0 suggests that an increase in gene expression levels in a particular tissue was associated with a decreased disease risk, and vice versa. The blue and orange dashed lines indicate a p-value of 0.05 and a Bonferroni-corrected p-value of 0.00001, respectively. The asterisk indicates HEIDI p-value greater than 0.01; AAo, Artery aorta; Atib, Artery tibial; BCH, Brain Cerebellar Hemisphere; BC, Brain Cerebellum; Thy, thyroid; HAA, Heart Atrial Appendage.


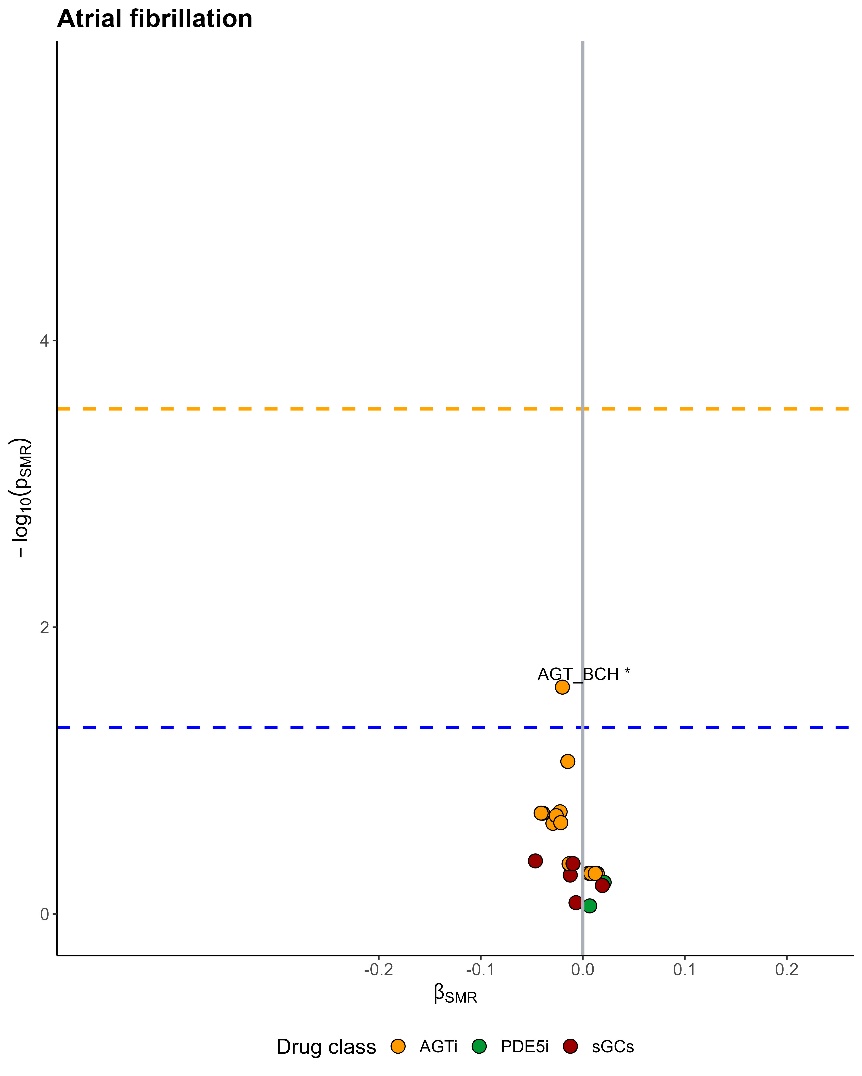

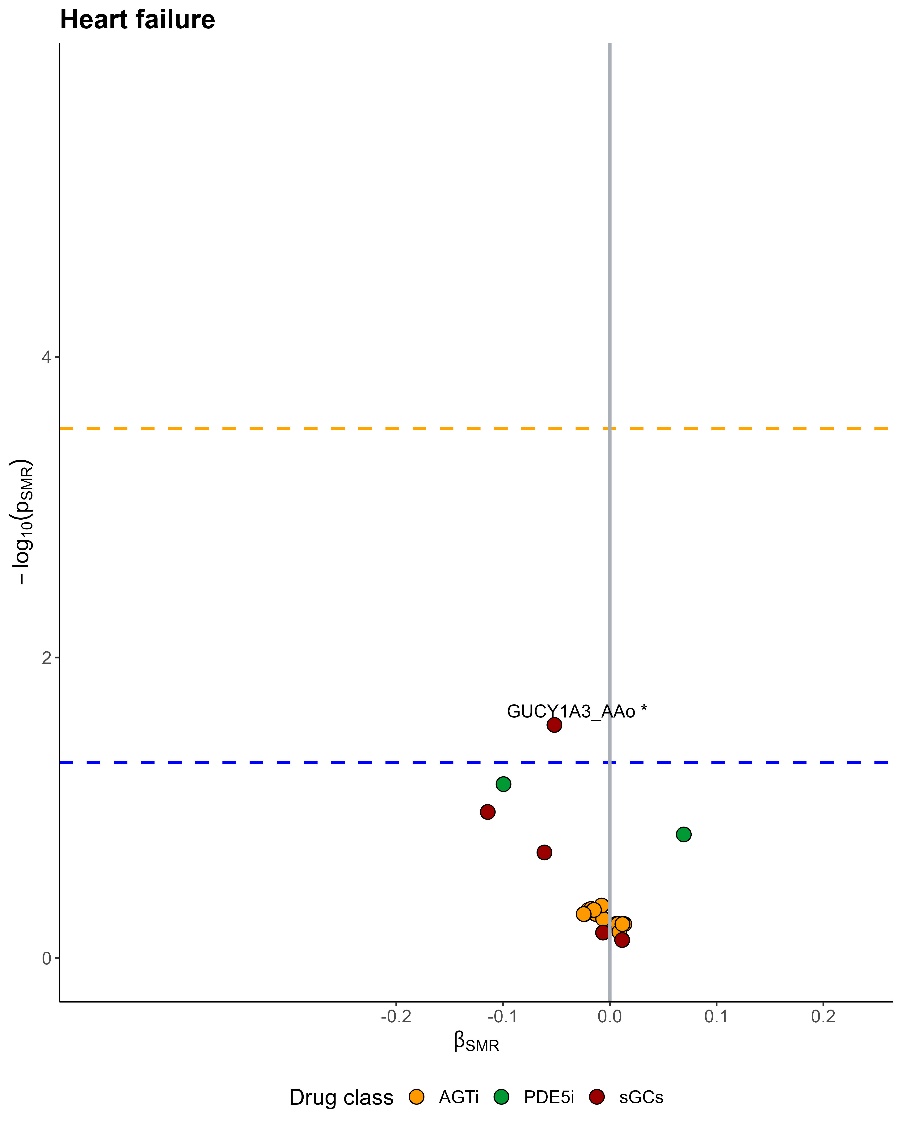

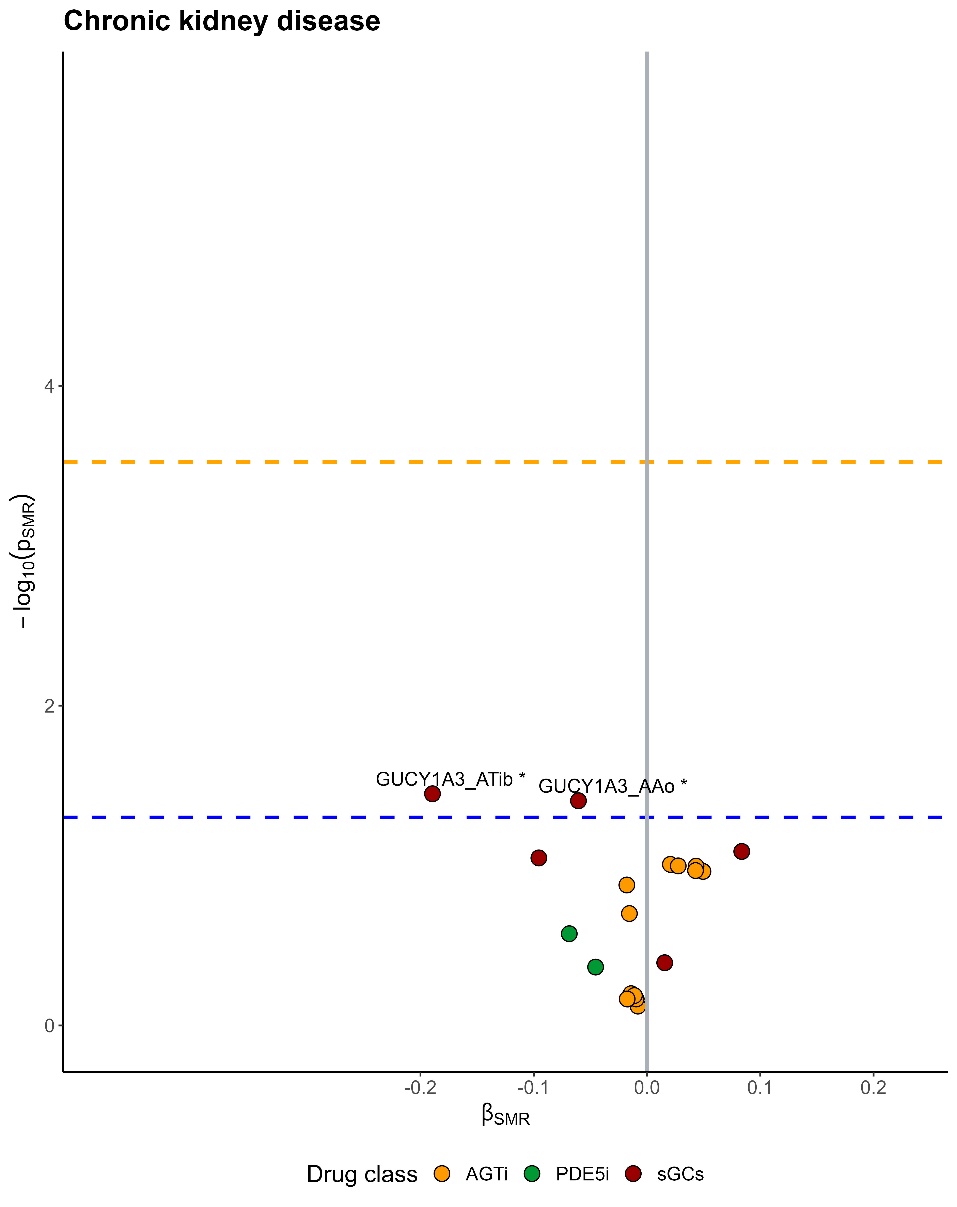

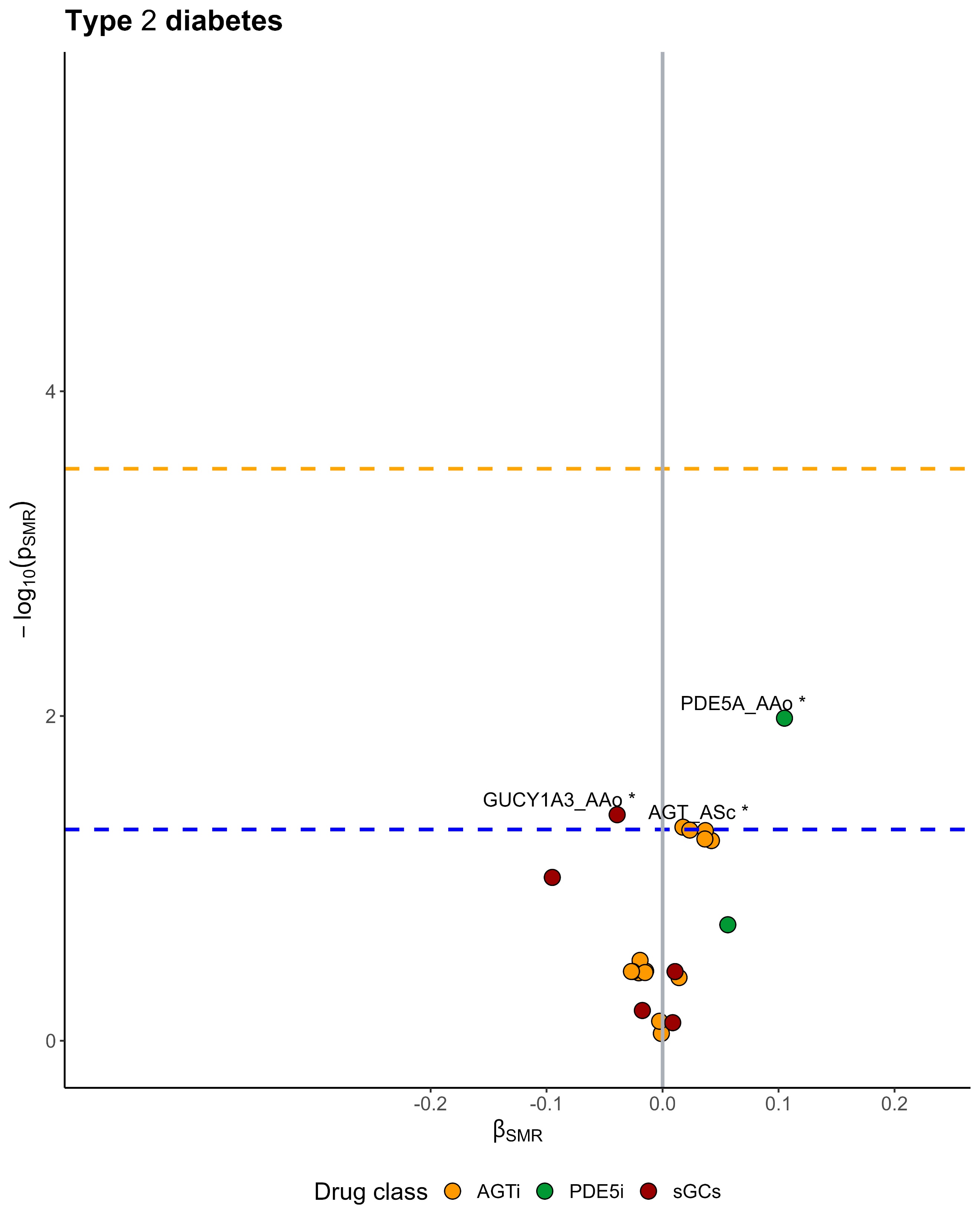


Figure S4. Colocalisation of eQTL in tibial artery for GUCY1A3 and CAD risk.

Colocalisation of genetic signal for GUCY1A3 levels and CAD outcome in the 1-Mb window of the gene. SNPs are coloured according to their LD r2 with the lead SNP rs72687508.


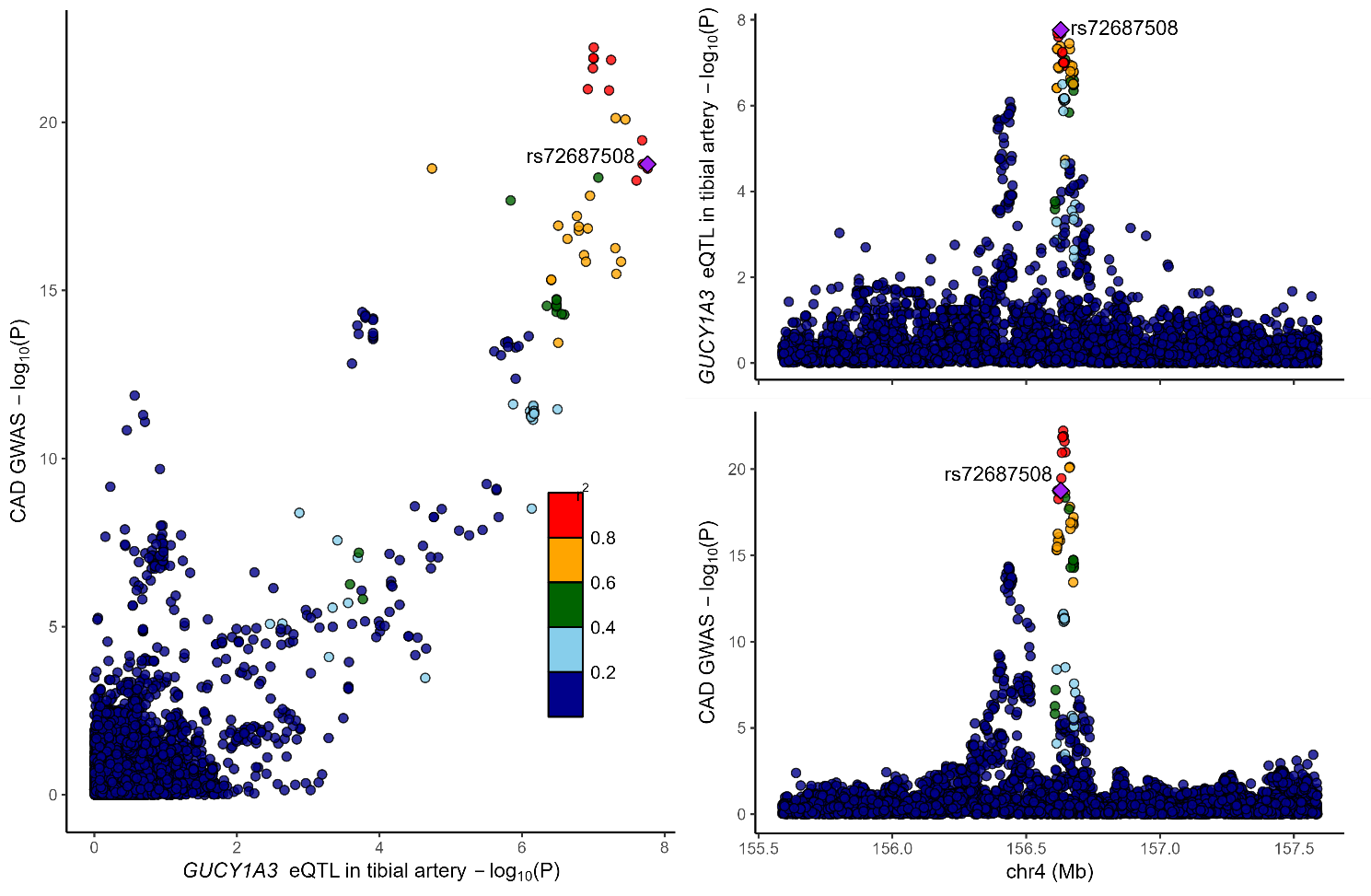


PP shared causal variant: 0.99

Table S1. Antihypertensive Drug Classes, Drug Names, and Corresponding Drug Target Genes.

AGTi, angiotensinogen inhibitors; ERAs, endothelin receptor antagonists; PDE5 inhibitors, phosphodiesterase-5 inhibitors; sGC stimulators, soluble guanylate cyclase stimulators.

| **Drug class** | **Drug name** | **Target gene** |
| --- | --- | --- |
| AGTi | zilebesiran | *AGT* |
| ERAs | ambrisentan | *EDNRA* |
|  | bosentan | *EDNRA, EDNRB* |
|  | macitentan | *EDNRA* |
|  | sitaxentan | *EDNRA* |
| PDE5 inhibitors | sildenafil | *PDE5A, NOS3* |
|  | tadalafil | *PDE5A* |
| sGC stimulators | riociguat | *GUCY1A2, GUCY1A3* |
|  | vericiguat | *GUCY1B1* |

Table S2. GWAS for negative control outcome analysis

| **Trait** | **Population** | **Sample size** |
| --- | --- | --- |
| Low hand grip strength (9) | European | 48,596 cases; 207,927 controls |
| Myopia (10) | European | 36,623 cases; 419,031 controls |
| Parkinson disease (11) | European | 15,056 cases, 18,618 proxy cases, 449,056 controls |
| Heel bone mineral density (12) | European | 583,314 individuals |

Table S3. Genetic instruments for systolic blood pressure

| **SNP** | **ea** | **nea** | **eaf** | **beta** | **se** | **p** | **n** | **chr** | **position** | **R^2^** | **F** |
| --- | --- | --- | --- | --- | --- | --- | --- | --- | --- | --- | --- |
| rs1006545 | T | G | 0.8872 | 0.6846 | 0.048 | 3.50E-46 | 738169 | 10 | 1.03E+08 | 0.000275 | 203.4184 |
| rs11191580 | T | C | 0.9176 | 1.0995 | 0.055 | 7.74E-89 | 738169 | 10 | 1.05E+08 | 0.000541 | 399.6354 |
| rs117464403 | A | G | 0.0183 | 0.864 | 0.1199 | 5.80E-13 | 727329 | 10 | 1.07E+08 | 7.14E-05 | 51.92637 |
| rs12255372 | T | G | 0.2883 | 0.2358 | 0.0335 | 1.94E-12 | 729908 | 10 | 1.15E+08 | 6.79E-05 | 49.54465 |
| rs1801253 | C | G | 0.7338 | 0.4626 | 0.0344 | 2.84E-41 | 738169 | 10 | 1.16E+08 | 0.000245 | 180.8395 |
| rs72842207 | T | C | 0.2144 | -0.203 | 0.0367 | 3.14E-08 | 738167 | 10 | 1.21E+08 | 4.14E-05 | 30.59559 |
| rs11592107 | A | G | 0.3096 | 0.3024 | 0.0326 | 1.55E-20 | 738169 | 10 | 1.23E+08 | 0.000117 | 86.04531 |
| rs7093894 | A | C | 0.1512 | 0.236 | 0.0427 | 3.16E-08 | 738169 | 10 | 1.24E+08 | 4.14E-05 | 30.5469 |
| rs7912283 | A | G | 0.6468 | -0.2144 | 0.0322 | 2.94E-11 | 736050 | 10 | 1.34E+08 | 6.02E-05 | 44.33397 |
| rs1133400 | A | G | 0.786 | -0.2975 | 0.0376 | 2.53E-15 | 722557 | 10 | 1.34E+08 | 8.66E-05 | 62.60327 |
| rs1623474 | T | C | 0.3303 | 0.3827 | 0.0321 | 7.66E-33 | 738167 | 10 | 18471794 | 0.000193 | 142.1365 |
| rs12258967 | C | G | 0.7047 | 0.6327 | 0.0337 | 1.08E-78 | 737165 | 10 | 18727959 | 0.000478 | 352.4802 |
| rs3802517 | A | T | 0.4618 | 0.2527 | 0.0301 | 4.65E-17 | 738169 | 10 | 28233469 | 9.55E-05 | 70.48169 |
| rs12264186 | T | C | 0.1871 | 0.2135 | 0.0387 | 3.58E-08 | 738168 | 10 | 32289986 | 4.12E-05 | 30.43495 |
| rs11252324 | T | G | 0.0771 | -0.4164 | 0.0573 | 3.61E-13 | 738167 | 10 | 4124568 | 7.15E-05 | 52.80937 |
| rs4948643 | T | C | 0.2819 | 0.2258 | 0.0338 | 2.40E-11 | 737164 | 10 | 45379759 | 6.05E-05 | 44.6286 |
| rs34130368 | T | G | 0.117 | -0.3016 | 0.0497 | 1.28E-09 | 736051 | 10 | 48411796 | 5.00E-05 | 36.82551 |
| rs4245599 | A | G | 0.4584 | -0.1794 | 0.0305 | 4.04E-09 | 738169 | 10 | 60365755 | 4.69E-05 | 34.59744 |
| rs57946343 | T | C | 0.8527 | 0.716 | 0.0426 | 2.10E-63 | 736110 | 10 | 63499951 | 0.000384 | 282.4917 |
| rs2236295 | T | G | 0.3978 | -0.3028 | 0.0309 | 1.05E-22 | 738169 | 10 | 64564892 | 0.00013 | 96.02705 |
| rs2177843 | T | C | 0.1505 | 0.4394 | 0.0432 | 2.80E-24 | 738168 | 10 | 75409877 | 0.00014 | 103.455 |
| rs10749572 | T | G | 0.5444 | -0.203 | 0.0302 | 1.88E-11 | 738170 | 10 | 82136664 | 6.12E-05 | 45.1832 |
| rs111866816 | T | C | 0.0709 | 0.3569 | 0.0597 | 2.29E-09 | 737163 | 10 | 94441507 | 4.85E-05 | 35.73907 |
| rs2689690 | T | C | 0.3678 | -0.2702 | 0.0316 | 1.15E-17 | 727391 | 10 | 95899706 | 0.000101 | 73.11312 |
| rs2274224 | C | G | 0.4324 | -0.4517 | 0.0304 | 5.99E-50 | 737056 | 10 | 96039597 | 0.000299 | 220.776 |
| rs604723 | T | C | 0.2756 | -0.655 | 0.0339 | 2.55E-83 | 737165 | 11 | 1.01E+08 | 0.000506 | 373.3207 |
| rs629864 | T | C | 0.6497 | -0.1868 | 0.0319 | 4.69E-09 | 737165 | 11 | 1.01E+08 | 4.65E-05 | 34.29029 |
| rs7926110 | T | G | 0.6733 | 0.2603 | 0.0321 | 5.71E-16 | 738168 | 11 | 1.07E+08 | 8.91E-05 | 65.75626 |
| rs236916 | A | G | 0.1348 | 0.3166 | 0.0446 | 1.31E-12 | 738167 | 11 | 1.17E+08 | 6.83E-05 | 50.39076 |
| rs573455 | A | G | 0.461 | 0.1994 | 0.0303 | 4.77E-11 | 737056 | 11 | 1.17E+08 | 5.88E-05 | 43.30758 |
| rs11222084 | A | T | 0.6379 | -0.3363 | 0.0316 | 1.80E-26 | 737164 | 11 | 1.3E+08 | 0.000154 | 113.2605 |
| rs7944927 | T | C | 0.7819 | 0.2235 | 0.0392 | 1.23E-08 | 737163 | 11 | 1.3E+08 | 4.41E-05 | 32.50736 |
| rs2014408 | T | C | 0.2087 | 0.5169 | 0.0373 | 1.26E-43 | 738168 | 11 | 16365282 | 0.00026 | 192.0411 |
| rs7926335 | T | C | 0.2691 | 0.3135 | 0.0339 | 2.52E-20 | 736109 | 11 | 16917869 | 0.000116 | 85.52134 |
| rs569550 | T | G | 0.6037 | -0.5765 | 0.0318 | 1.33E-73 | 718172 | 11 | 1887068 | 0.000457 | 328.6572 |
| rs74048190 | T | C | 0.9522 | -0.4404 | 0.0757 | 6.07E-09 | 718169 | 11 | 2114221 | 4.71E-05 | 33.84556 |
| rs17762 | A | G | 0.0777 | 0.4117 | 0.0571 | 5.60E-13 | 738167 | 11 | 22492454 | 7.04E-05 | 51.98623 |
| rs1382472 | A | G | 0.4041 | -0.1917 | 0.0307 | 4.47E-10 | 738170 | 11 | 27273967 | 5.28E-05 | 38.99117 |
| rs871004 | A | G | 0.3481 | 0.2336 | 0.0317 | 1.65E-13 | 738170 | 11 | 28512458 | 7.36E-05 | 54.30327 |
| rs10501122 | T | C | 0.639 | 0.1916 | 0.0315 | 1.18E-09 | 737164 | 11 | 30192151 | 5.02E-05 | 36.99719 |
| rs11604310 | T | C | 0.1655 | -0.2778 | 0.0411 | 1.46E-11 | 738168 | 11 | 45351420 | 6.19E-05 | 45.68563 |
| rs7107356 | A | G | 0.4959 | -0.4598 | 0.0301 | 1.63E-52 | 738170 | 11 | 47676170 | 0.000316 | 233.3478 |
| rs2904315 | A | G | 0.3131 | -0.2081 | 0.0325 | 1.58E-10 | 738167 | 11 | 48109948 | 5.55E-05 | 40.99928 |
| rs4427587 | T | C | 0.5619 | 0.2062 | 0.0313 | 4.28E-11 | 737164 | 11 | 58436648 | 5.89E-05 | 43.39977 |
| rs7125196 | T | C | 0.8817 | 0.4422 | 0.0472 | 7.31E-21 | 736058 | 11 | 61272565 | 0.000119 | 87.77125 |
| rs2306363 | T | G | 0.2045 | -0.4358 | 0.0376 | 5.24E-31 | 738167 | 11 | 65405600 | 0.000182 | 134.3376 |
| rs7395791 | A | G | 0.4419 | -0.2162 | 0.0308 | 2.19E-12 | 738170 | 11 | 69262916 | 6.67E-05 | 49.27297 |
| rs10501410 | A | G | 0.0692 | 0.4122 | 0.0607 | 1.10E-11 | 738166 | 11 | 72088806 | 6.25E-05 | 46.11449 |
| rs7927515 | A | C | 0.3459 | 0.2271 | 0.0319 | 1.05E-12 | 729908 | 11 | 76125330 | 6.94E-05 | 50.68176 |
| rs2289124 | A | G | 0.1673 | -0.308 | 0.0415 | 1.14E-13 | 737164 | 11 | 89224477 | 7.47E-05 | 55.08128 |
| rs360153 | T | C | 0.4166 | -0.3445 | 0.0306 | 1.73E-29 | 738170 | 11 | 9762274 | 0.000172 | 126.746 |
| rs5742643 | T | C | 0.2487 | -0.2233 | 0.0349 | 1.53E-10 | 740938 | 12 | 1.03E+08 | 5.52E-05 | 40.93789 |
| rs7310615 | C | G | 0.4816 | 0.585 | 0.0306 | 1.32E-81 | 737101 | 12 | 1.12E+08 | 0.000496 | 365.4834 |
| rs1896326 | A | G | 0.2291 | -0.2797 | 0.0371 | 4.41E-14 | 743700 | 12 | 1.15E+08 | 7.64E-05 | 56.83763 |
| rs35444 | A | G | 0.6138 | 0.4368 | 0.031 | 3.47E-45 | 737556 | 12 | 1.16E+08 | 0.000269 | 198.5367 |
| rs6490019 | A | G | 0.3796 | -0.2897 | 0.0309 | 6.61E-21 | 745818 | 12 | 1.16E+08 | 0.000118 | 87.89797 |
| rs1169078 | C | G | 0.6879 | -0.1971 | 0.0327 | 1.68E-09 | 744811 | 12 | 1.22E+08 | 4.88E-05 | 36.33094 |
| rs2024385 | A | T | 0.424 | -0.2642 | 0.0306 | 5.88E-18 | 745819 | 12 | 12888438 | 9.99E-05 | 74.54553 |
| rs117206641 | T | C | 0.1108 | 0.3154 | 0.0499 | 2.66E-10 | 725612 | 12 | 1.33E+08 | 5.51E-05 | 39.9504 |
| rs1010064 | A | C | 0.8163 | 0.3571 | 0.0387 | 3.02E-20 | 745818 | 12 | 20000315 | 0.000114 | 85.1445 |
| rs73075659 | A | G | 0.6654 | 0.3962 | 0.0321 | 5.52E-35 | 744703 | 12 | 20373541 | 0.000205 | 152.3413 |
| rs3819532 | T | C | 0.3913 | -0.1875 | 0.0306 | 9.44E-10 | 744814 | 12 | 2436837 | 5.04E-05 | 37.54555 |
| rs2129869 | A | T | 0.7778 | -0.2643 | 0.0361 | 2.44E-13 | 743761 | 12 | 26457650 | 7.21E-05 | 53.60172 |
| rs9651825 | A | G | 0.7295 | -0.2042 | 0.034 | 1.93E-09 | 737555 | 12 | 27159784 | 4.89E-05 | 36.07053 |
| rs78998485 | C | G | 0.7443 | -0.2449 | 0.0346 | 1.48E-12 | 744814 | 12 | 434755 | 6.73E-05 | 50.09844 |
| rs61917655 | T | C | 0.1014 | 0.3427 | 0.0514 | 2.68E-11 | 745817 | 12 | 48210787 | 5.96E-05 | 44.45297 |
| rs12426261 | A | G | 0.3792 | 0.3775 | 0.0309 | 2.31E-34 | 745819 | 12 | 50573037 | 0.0002 | 149.2505 |
| rs7134440 | T | C | 0.0822 | 0.4788 | 0.0562 | 1.58E-17 | 745819 | 12 | 53450097 | 9.73E-05 | 72.58293 |
| rs7134677 | T | C | 0.2978 | -0.3851 | 0.0332 | 4.46E-31 | 744813 | 12 | 54441498 | 0.000181 | 134.5457 |
| rs7306710 | T | C | 0.481 | -0.2429 | 0.0303 | 1.03E-15 | 745819 | 12 | 66376091 | 8.62E-05 | 64.26413 |
| rs4143175 | T | C | 0.2409 | 0.2187 | 0.0352 | 5.10E-10 | 744706 | 12 | 67782397 | 5.18E-05 | 38.60211 |
| rs7963801 | T | C | 0.4221 | -0.2362 | 0.0311 | 2.87E-14 | 744815 | 12 | 79685226 | 7.74E-05 | 57.68167 |
| rs6539467 | A | G | 0.1661 | 0.265 | 0.0404 | 5.57E-11 | 745818 | 12 | 79955306 | 5.77E-05 | 43.02569 |
| rs17249754 | A | G | 0.1683 | -0.8446 | 0.0403 | 1.25E-97 | 743707 | 12 | 90060586 | 0.00059 | 439.2289 |
| rs10777213 | A | G | 0.5244 | -0.1786 | 0.0299 | 2.45E-09 | 745820 | 12 | 90349999 | 4.78E-05 | 35.67955 |
| rs9549627 | A | G | 0.1175 | 0.2846 | 0.05 | 1.25E-08 | 729202 | 13 | 1.14E+08 | 4.44E-05 | 32.39878 |
| rs7331680 | T | G | 0.1491 | 0.4101 | 0.0423 | 3.35E-22 | 742899 | 13 | 1.15E+08 | 0.000127 | 93.99346 |
| rs483071 | T | C | 0.6248 | 0.2709 | 0.0313 | 5.09E-18 | 744705 | 13 | 22294117 | 0.000101 | 74.90799 |
| rs9507885 | T | C | 0.0953 | -0.3208 | 0.0542 | 3.23E-09 | 740743 | 13 | 27951090 | 4.73E-05 | 35.03233 |
| rs7338758 | T | C | 0.2448 | 0.3552 | 0.0352 | 7.02E-24 | 737099 | 13 | 30137828 | 0.000138 | 101.8262 |
| rs2065498 | T | G | 0.1706 | -0.2934 | 0.0403 | 3.36E-13 | 745818 | 13 | 41893105 | 7.11E-05 | 53.00404 |
| rs7491248 | A | G | 0.2239 | 0.2163 | 0.0362 | 2.38E-09 | 737558 | 13 | 47180671 | 4.84E-05 | 35.70218 |
| rs9526707 | A | G | 0.3216 | -0.2039 | 0.0323 | 2.77E-10 | 744706 | 13 | 51489186 | 5.35E-05 | 39.84999 |
| rs75961402 | A | G | 0.1534 | 0.2659 | 0.0418 | 1.95E-10 | 745819 | 13 | 56398286 | 5.43E-05 | 40.46532 |
| rs17245822 | A | C | 0.6267 | -0.1899 | 0.0312 | 1.15E-09 | 745819 | 13 | 73131694 | 4.97E-05 | 37.04585 |
| rs78474310 | A | G | 0.9552 | -0.4699 | 0.0734 | 1.51E-10 | 745820 | 13 | 73826901 | 5.49E-05 | 40.98431 |
| rs6562778 | A | G | 0.4589 | 0.178 | 0.0304 | 4.96E-09 | 744815 | 13 | 74223828 | 4.60E-05 | 34.28401 |
| rs17562391 | T | C | 0.4186 | 0.1967 | 0.0306 | 1.35E-10 | 745818 | 14 | 1E+08 | 5.54E-05 | 41.32042 |
| rs75016974 | T | C | 0.1423 | -0.2513 | 0.0439 | 1.05E-08 | 744815 | 14 | 1E+08 | 4.40E-05 | 32.76836 |
| rs12885878 | A | G | 0.2337 | -0.2291 | 0.0367 | 4.32E-10 | 744814 | 14 | 1.04E+08 | 5.23E-05 | 38.96879 |
| rs365990 | A | G | 0.6342 | 0.225 | 0.0312 | 5.95E-13 | 745820 | 14 | 23861811 | 6.97E-05 | 52.00615 |
| rs8904 | A | G | 0.3678 | 0.3061 | 0.0314 | 1.71E-22 | 739799 | 14 | 35871217 | 0.000128 | 95.03119 |
| rs7493678 | A | T | 0.6514 | -0.189 | 0.0316 | 2.31E-09 | 745819 | 14 | 39400917 | 4.80E-05 | 35.77242 |
| rs72683923 | T | C | 0.9788 | 0.9587 | 0.1101 | 3.08E-18 | 743244 | 14 | 50735947 | 0.000102 | 75.82102 |
| rs35413927 | A | G | 0.6946 | -0.3002 | 0.0328 | 5.25E-20 | 745820 | 14 | 53420358 | 0.000112 | 83.76692 |
| rs57140819 | C | G | 0.8268 | 0.2415 | 0.0398 | 1.30E-09 | 745819 | 14 | 68018247 | 4.94E-05 | 36.81857 |
| rs11847049 | C | G | 0.7834 | -0.2272 | 0.0364 | 4.44E-10 | 745819 | 14 | 69259406 | 5.22E-05 | 38.95944 |
| rs11159091 | A | G | 0.4615 | 0.1978 | 0.0303 | 6.79E-11 | 735987 | 14 | 75074316 | 5.79E-05 | 42.61536 |
| rs7154723 | A | G | 0.385 | 0.253 | 0.0309 | 2.72E-16 | 744815 | 14 | 98590629 | 9.00E-05 | 67.03829 |
| rs4606697 | A | G | 0.1041 | -0.3196 | 0.0523 | 9.71E-10 | 740084 | 15 | 1E+08 | 5.05E-05 | 37.34298 |
| rs8030856 | C | G | 0.6047 | -0.1764 | 0.031 | 1.21E-08 | 744815 | 15 | 40314967 | 4.35E-05 | 32.37968 |
| rs28866311 | T | G | 0.5263 | -0.2762 | 0.0302 | 5.45E-20 | 745820 | 15 | 41442195 | 0.000112 | 83.64352 |
| rs4775769 | T | G | 0.0945 | -0.4162 | 0.0517 | 7.76E-16 | 745818 | 15 | 48939888 | 8.69E-05 | 64.807 |
| rs3098186 | T | C | 0.5156 | -0.2422 | 0.0303 | 1.41E-15 | 745820 | 15 | 50810621 | 8.57E-05 | 63.89426 |
| rs2652812 | T | C | 0.7544 | -0.2516 | 0.0353 | 1.03E-12 | 744814 | 15 | 63406170 | 6.82E-05 | 50.80082 |
| rs28429256 | A | G | 0.3342 | 0.215 | 0.0325 | 3.89E-11 | 743700 | 15 | 66931617 | 5.88E-05 | 43.7632 |
| rs11636952 | T | C | 0.3141 | 0.5313 | 0.0328 | 4.22E-59 | 727894 | 15 | 75114322 | 0.00036 | 262.38 |
| rs2627313 | T | C | 0.4454 | 0.3208 | 0.0303 | 3.55E-26 | 737101 | 15 | 81006712 | 0.000152 | 112.094 |
| rs2046341 | A | G | 0.1921 | -0.2542 | 0.0382 | 2.74E-11 | 742594 | 15 | 86040872 | 5.96E-05 | 44.28159 |
| rs77032376 | T | C | 0.1485 | -0.2727 | 0.043 | 2.35E-10 | 738798 | 15 | 90010780 | 5.44E-05 | 40.21909 |
| rs4932373 | A | C | 0.6742 | -0.635 | 0.0328 | 2.49E-83 | 724766 | 15 | 91429287 | 0.000517 | 374.7991 |
| rs12906962 | T | C | 0.676 | -0.2653 | 0.0325 | 3.28E-16 | 742702 | 15 | 95312071 | 8.97E-05 | 66.63565 |
| rs2589218 | T | C | 0.7297 | -0.2258 | 0.0339 | 2.54E-11 | 743708 | 15 | 96785017 | 5.97E-05 | 44.3657 |
| rs11641374 | A | C | 0.5995 | -0.1943 | 0.0309 | 3.26E-10 | 741588 | 16 | 1347717 | 5.33E-05 | 39.53916 |
| rs77924615 | A | G | 0.1986 | -0.4081 | 0.039 | 1.12E-25 | 743700 | 16 | 20392332 | 0.000147 | 109.4971 |
| rs12596630 | T | C | 0.0903 | 0.4278 | 0.0547 | 5.01E-15 | 723276 | 16 | 2065666 | 8.46E-05 | 61.16538 |
| rs7186298 | T | C | 0.4295 | -0.2315 | 0.0302 | 1.88E-14 | 745820 | 16 | 21088031 | 7.88E-05 | 58.7607 |
| rs8044992 | T | C | 0.7123 | 0.2138 | 0.0331 | 1.07E-10 | 745819 | 16 | 24811207 | 5.59E-05 | 41.72134 |
| rs72778133 | T | C | 0.8578 | -0.2417 | 0.0443 | 4.98E-08 | 744813 | 16 | 3578718 | 4.00E-05 | 29.76766 |
| rs111929315 | A | G | 0.8917 | 0.3146 | 0.0485 | 8.60E-11 | 745818 | 16 | 4136871 | 5.64E-05 | 42.07584 |
| rs12446456 | T | C | 0.4274 | -0.3003 | 0.0302 | 2.97E-23 | 745820 | 16 | 4922201 | 0.000133 | 98.87708 |
| rs34941092 | A | G | 0.1498 | -0.3225 | 0.0425 | 3.23E-14 | 745818 | 16 | 50550137 | 7.72E-05 | 57.58116 |
| rs4784541 | T | C | 0.4748 | -0.2015 | 0.0307 | 4.93E-11 | 744815 | 16 | 51704452 | 5.78E-05 | 43.07965 |
| rs2060664 | T | C | 0.7484 | 0.216 | 0.0345 | 4.06E-10 | 744812 | 16 | 60652439 | 5.26E-05 | 39.19838 |
| rs146550789 | T | C | 0.9583 | -0.4824 | 0.0778 | 5.64E-10 | 745818 | 16 | 66781040 | 5.15E-05 | 38.44627 |
| rs62047964 | T | C | 0.0622 | 0.5115 | 0.0686 | 9.29E-14 | 736711 | 16 | 70729954 | 7.55E-05 | 55.59578 |
| rs1012089 | C | G | 0.4752 | -0.192 | 0.0302 | 1.95E-10 | 745819 | 16 | 74171973 | 5.42E-05 | 40.41917 |
| rs4888408 | A | G | 0.5855 | 0.3653 | 0.0307 | 1.42E-32 | 744815 | 16 | 75432824 | 0.00019 | 141.5864 |
| rs12926550 | A | G | 0.3156 | -0.2548 | 0.0324 | 3.43E-15 | 745819 | 16 | 81510155 | 8.29E-05 | 61.84544 |
| rs3950627 | A | C | 0.531 | 0.1851 | 0.0308 | 1.82E-09 | 738794 | 16 | 86436343 | 4.89E-05 | 36.11688 |
| rs6540119 | A | T | 0.334 | 0.2016 | 0.0322 | 3.93E-10 | 745819 | 16 | 87984477 | 5.26E-05 | 39.19838 |
| rs908951 | T | C | 0.4378 | -0.2261 | 0.0315 | 7.14E-13 | 734063 | 16 | 89697625 | 7.02E-05 | 51.52035 |
| rs9303175 | T | G | 0.3463 | -0.2048 | 0.0327 | 3.65E-10 | 743700 | 17 | 1372987 | 5.27E-05 | 39.22503 |
| rs4925159 | A | G | 0.4246 | 0.2174 | 0.0305 | 9.66E-13 | 737558 | 17 | 18185510 | 6.89E-05 | 50.80638 |
| rs7218708 | A | G | 0.4831 | -0.1781 | 0.0303 | 4.38E-09 | 737558 | 17 | 19926836 | 4.68E-05 | 34.54947 |
| rs11653927 | T | C | 0.3845 | -0.2796 | 0.0308 | 1.17E-19 | 745820 | 17 | 2012094 | 0.00011 | 82.40845 |
| rs1551355 | T | C | 0.2334 | 0.2098 | 0.0356 | 3.89E-09 | 745818 | 17 | 30032420 | 4.66E-05 | 34.7304 |
| rs9899540 | A | T | 0.3999 | 0.2011 | 0.0316 | 1.87E-10 | 744813 | 17 | 30777924 | 5.44E-05 | 40.49942 |
| rs7213273 | A | G | 0.655 | -0.4 | 0.0315 | 6.24E-37 | 745819 | 17 | 43155914 | 0.000216 | 161.2493 |
| rs17608766 | T | C | 0.8555 | -0.6903 | 0.0433 | 2.48E-57 | 737558 | 17 | 45013271 | 0.000344 | 254.1551 |
| rs3764400 | T | C | 0.8635 | 0.3748 | 0.0445 | 3.69E-17 | 744815 | 17 | 46123932 | 9.52E-05 | 70.93784 |
| rs9897429 | A | G | 0.52 | 0.2645 | 0.0319 | 1.19E-16 | 744815 | 17 | 47518378 | 9.23E-05 | 68.74939 |
| rs1000423 | T | C | 0.7316 | 0.4138 | 0.0346 | 6.50E-33 | 737099 | 17 | 59475642 | 0.000194 | 143.0302 |
| rs56288724 | A | G | 0.5831 | -0.2178 | 0.031 | 2.01E-12 | 744706 | 17 | 60767135 | 6.63E-05 | 49.36182 |
| rs62076622 | A | G | 0.8013 | 0.2363 | 0.0377 | 3.79E-10 | 745819 | 17 | 61090958 | 5.27E-05 | 39.28652 |
| rs6504213 | T | C | 0.4182 | -0.2982 | 0.0312 | 1.25E-21 | 744814 | 17 | 62381714 | 0.000123 | 91.34924 |
| rs113086489 | T | C | 0.5525 | 0.3249 | 0.0307 | 3.80E-26 | 744814 | 17 | 7171356 | 0.00015 | 112.0009 |
| rs4511593 | T | C | 0.6528 | -0.2881 | 0.0318 | 1.28E-19 | 737557 | 17 | 7455536 | 0.000111 | 82.07882 |
| rs1436138 | A | G | 0.6367 | 0.3119 | 0.0315 | 4.73E-23 | 744705 | 17 | 75316880 | 0.000132 | 98.04117 |
| rs9302885 | A | G | 0.4452 | 0.2242 | 0.0302 | 1.03E-13 | 744706 | 17 | 76799898 | 7.40E-05 | 55.11327 |
| rs117285318 | T | C | 0.9225 | 0.4413 | 0.0589 | 6.93E-14 | 744704 | 17 | 7870642 | 7.54E-05 | 56.13531 |
| rs11655604 | T | C | 0.3579 | -0.2033 | 0.0333 | 1.09E-09 | 699816 | 17 | 79365861 | 5.33E-05 | 37.2722 |
| rs62082230 | A | T | 0.2773 | -0.1884 | 0.0345 | 4.69E-08 | 744814 | 18 | 22676071 | 4.00E-05 | 29.82102 |
| rs1154214 | T | G | 0.3963 | -0.2031 | 0.0306 | 3.27E-11 | 744706 | 18 | 24546824 | 5.92E-05 | 44.05303 |
| rs56407827 | T | C | 0.2687 | 0.3603 | 0.034 | 2.78E-26 | 744704 | 18 | 42179819 | 0.000151 | 112.2974 |
| rs11874246 | T | C | 0.2963 | 0.2856 | 0.0328 | 3.23E-18 | 745818 | 18 | 42596789 | 0.000102 | 75.81717 |
| rs7245140 | T | C | 0.8198 | -0.3367 | 0.0391 | 7.67E-18 | 745820 | 18 | 43095231 | 9.94E-05 | 74.15348 |
| rs1437649 | A | G | 0.2345 | -0.2189 | 0.0357 | 8.57E-10 | 745819 | 18 | 48132646 | 5.04E-05 | 37.59706 |
| rs665445 | A | C | 0.2794 | -0.1909 | 0.0334 | 1.15E-08 | 745819 | 18 | 51842682 | 4.38E-05 | 32.66764 |
| rs10048404 | T | C | 0.3701 | -0.2607 | 0.0317 | 1.91E-16 | 744815 | 18 | 54578482 | 9.08E-05 | 67.63358 |
| rs10460108 | A | G | 0.4801 | 0.2141 | 0.0301 | 1.12E-12 | 745819 | 18 | 73034151 | 6.78E-05 | 50.59402 |
| rs34413141 | A | T | 0.1822 | -0.3531 | 0.0393 | 2.47E-19 | 745818 | 18 | 777282 | 0.000108 | 80.72521 |
| rs167479 | T | G | 0.4726 | -0.5642 | 0.0327 | 7.21E-67 | 675533 | 19 | 11526765 | 0.00044 | 297.6935 |
| rs698748 | A | G | 0.421 | 0.1871 | 0.0325 | 8.90E-09 | 722073 | 19 | 1424888 | 4.59E-05 | 33.14207 |
| rs1077795 | A | G | 0.7393 | 0.2507 | 0.0344 | 3.33E-13 | 744813 | 19 | 17222584 | 7.13E-05 | 53.11175 |
| rs149339216 | T | C | 0.9566 | -0.6912 | 0.0779 | 6.93E-19 | 724208 | 19 | 2144046 | 0.000109 | 78.72839 |
| rs62112908 | A | G | 0.8464 | -0.2388 | 0.0419 | 1.25E-08 | 745818 | 19 | 22213956 | 4.36E-05 | 32.48175 |
| rs28572357 | A | C | 0.6023 | -0.2733 | 0.0308 | 6.34E-19 | 741283 | 19 | 31867447 | 0.000106 | 78.7366 |
| rs1433121 | T | C | 0.6906 | -0.228 | 0.0326 | 2.66E-12 | 745817 | 19 | 32591878 | 6.56E-05 | 48.91402 |
| rs33836 | T | C | 0.4622 | 0.1766 | 0.0304 | 6.56E-09 | 745820 | 19 | 34008600 | 4.52E-05 | 33.74684 |
| rs10420519 | T | G | 0.0347 | -0.4921 | 0.0887 | 2.86E-08 | 738581 | 19 | 45298461 | 4.17E-05 | 30.77927 |
| rs7255933 | A | G | 0.2574 | 0.2306 | 0.0345 | 2.44E-11 | 745819 | 19 | 45766729 | 5.99E-05 | 44.67651 |
| rs11672660 | T | C | 0.1996 | 0.2212 | 0.0381 | 6.32E-09 | 737033 | 19 | 46180184 | 4.57E-05 | 33.70692 |
| rs571689 | T | C | 0.5196 | 0.228 | 0.0304 | 6.77E-14 | 737035 | 19 | 49207554 | 7.63E-05 | 56.24985 |
| rs73046792 | A | G | 0.1588 | -0.3554 | 0.0426 | 7.23E-17 | 737035 | 19 | 49605705 | 9.44E-05 | 69.60084 |
| rs68096471 | A | G | 0.2659 | -0.2098 | 0.0343 | 9.26E-10 | 745819 | 19 | 5175709 | 5.02E-05 | 37.41292 |
| rs12985940 | T | C | 0.8408 | 0.4642 | 0.0434 | 1.08E-26 | 721439 | 19 | 7262734 | 0.000159 | 114.4009 |
| rs488834 | T | C | 0.7645 | -0.3799 | 0.0365 | 2.35E-25 | 724655 | 1 | 10767902 | 0.000149 | 108.3307 |
| rs10776752 | T | G | 0.0809 | 0.8211 | 0.0576 | 4.61E-46 | 738168 | 1 | 1.13E+08 | 0.000275 | 203.2104 |
| rs59980837 | T | G | 0.0178 | 1.0997 | 0.1163 | 3.32E-21 | 734855 | 1 | 1.16E+08 | 0.000122 | 89.41036 |
| rs6699618 | C | G | 0.8401 | 0.9115 | 0.041 | 1.68E-109 | 738170 | 1 | 11881441 | 0.000669 | 494.2475 |
| rs11585169 | A | T | 0.5773 | 0.1796 | 0.0308 | 5.34E-09 | 728445 | 1 | 1.51E+08 | 4.67E-05 | 34.00244 |
| rs76719272 | T | C | 0.1312 | -0.2738 | 0.0461 | 2.97E-09 | 737164 | 1 | 1.56E+08 | 4.78E-05 | 35.27474 |
| rs75461554 | T | C | 0.2007 | -0.3016 | 0.0377 | 1.18E-15 | 738168 | 1 | 15810172 | 8.67E-05 | 63.99983 |
| rs1889785 | A | G | 0.4552 | 0.1782 | 0.0304 | 4.35E-09 | 738170 | 1 | 16348729 | 4.65E-05 | 34.3611 |
| rs7796 | C | G | 0.5114 | 0.3385 | 0.0314 | 5.00E-27 | 726899 | 1 | 1684169 | 0.00016 | 116.2136 |
| rs12731646 | T | C | 0.409 | -0.189 | 0.0307 | 7.21E-10 | 737225 | 1 | 1.69E+08 | 5.14E-05 | 37.90056 |
| rs1043069 | T | G | 0.6156 | 0.234 | 0.0311 | 5.26E-14 | 738169 | 1 | 1.81E+08 | 7.67E-05 | 56.61216 |
| rs4651224 | T | C | 0.4474 | 0.1986 | 0.0306 | 9.00E-11 | 737054 | 1 | 1.85E+08 | 5.71E-05 | 42.12253 |
| rs12042924 | T | C | 0.5284 | -0.1807 | 0.0303 | 2.62E-09 | 738170 | 1 | 1.97E+08 | 4.82E-05 | 35.56558 |
| rs11120093 | T | C | 0.4082 | -0.1792 | 0.0307 | 5.13E-09 | 738170 | 1 | 2.07E+08 | 4.62E-05 | 34.07204 |
| rs2724377 | A | G | 0.5303 | 0.1938 | 0.0301 | 1.29E-10 | 738170 | 1 | 2.08E+08 | 5.62E-05 | 41.45466 |
| rs7555285 | C | G | 0.8011 | 0.2294 | 0.0376 | 1.05E-09 | 738168 | 1 | 2.1E+08 | 5.04E-05 | 37.22288 |
| rs263532 | T | C | 0.5755 | 0.1798 | 0.0307 | 4.72E-09 | 735052 | 1 | 2164116 | 4.67E-05 | 34.30058 |
| rs68085857 | T | C | 0.234 | 0.274 | 0.0357 | 1.68E-14 | 738167 | 1 | 2.18E+08 | 7.98E-05 | 58.90654 |
| rs4595370 | A | G | 0.3012 | -0.2092 | 0.0328 | 1.73E-10 | 738168 | 1 | 2.21E+08 | 5.51E-05 | 40.6794 |
| rs1745417 | T | C | 0.5201 | 0.2871 | 0.0301 | 1.59E-21 | 738170 | 1 | 2.28E+08 | 0.000123 | 90.97713 |
| rs699 | A | G | 0.5928 | -0.3748 | 0.0308 | 5.59E-34 | 721189 | 1 | 2.31E+08 | 0.000205 | 148.08 |
| rs1565440 | A | G | 0.3752 | 0.1746 | 0.0311 | 1.94E-08 | 738169 | 1 | 2.43E+08 | 4.27E-05 | 31.51857 |
| rs4926499 | C | G | 0.8263 | 0.2965 | 0.0438 | 1.33E-11 | 711084 | 1 | 2.49E+08 | 6.44E-05 | 45.82473 |
| rs404100 | T | C | 0.4513 | 0.1935 | 0.0303 | 1.68E-10 | 737164 | 1 | 25366987 | 5.53E-05 | 40.78266 |
| rs34079867 | T | C | 0.266 | 0.1992 | 0.0354 | 1.78E-08 | 737163 | 1 | 27407850 | 4.30E-05 | 31.66438 |
| rs4908348 | T | G | 0.6944 | 0.2366 | 0.033 | 8.07E-13 | 737164 | 1 | 28706949 | 6.97E-05 | 51.40442 |
| rs2493296 | T | C | 0.1425 | 0.4183 | 0.0442 | 3.14E-21 | 724085 | 1 | 3327032 | 0.000124 | 89.56328 |
| rs11210029 | A | G | 0.6322 | -0.203 | 0.0313 | 8.92E-11 | 737056 | 1 | 41865293 | 5.71E-05 | 42.06319 |
| rs1408945 | T | G | 0.4243 | -0.3196 | 0.0304 | 8.33E-26 | 737056 | 1 | 42364877 | 0.00015 | 110.5262 |
| rs1209384 | A | G | 0.3878 | 0.2558 | 0.0313 | 2.85E-16 | 733288 | 1 | 43765089 | 9.11E-05 | 66.78997 |
| rs778124 | A | G | 0.3736 | 0.2965 | 0.0311 | 1.45E-21 | 738170 | 1 | 56606206 | 0.000123 | 90.89237 |
| rs61772592 | A | G | 0.8745 | -0.3181 | 0.0455 | 2.86E-12 | 738170 | 1 | 56979681 | 6.62E-05 | 48.87687 |
| rs12063372 | A | G | 0.3846 | 0.1989 | 0.0318 | 3.86E-10 | 737165 | 1 | 59621911 | 5.31E-05 | 39.12138 |
| rs2232460 | A | G | 0.3343 | -0.2171 | 0.032 | 1.10E-11 | 737056 | 1 | 6659505 | 6.24E-05 | 46.02762 |
| rs12136922 | A | G | 0.4949 | 0.2027 | 0.0304 | 2.69E-11 | 719076 | 1 | 67007389 | 6.18E-05 | 44.45894 |
| rs658780 | T | G | 0.7447 | -0.2028 | 0.0347 | 5.29E-09 | 737164 | 1 | 78555928 | 4.63E-05 | 34.15669 |
| rs786923 | T | C | 0.6239 | -0.3082 | 0.031 | 2.83E-23 | 737055 | 1 | 89242954 | 0.000134 | 98.84181 |
| rs7514579 | A | C | 0.7712 | 0.2243 | 0.0361 | 5.45E-10 | 738168 | 1 | 94051350 | 5.23E-05 | 38.60495 |
| rs2423514 | A | G | 0.5411 | 0.3011 | 0.0302 | 1.77E-23 | 745820 | 20 | 10693337 | 0.000133 | 99.4046 |
| rs6108787 | T | G | 0.5296 | -0.4274 | 0.03 | 5.38E-46 | 743761 | 20 | 10967214 | 0.000273 | 202.967 |
| rs6078093 | A | G | 0.428 | -0.1849 | 0.0304 | 1.20E-09 | 744815 | 20 | 11168669 | 4.97E-05 | 36.99351 |
| rs8125763 | A | C | 0.4717 | 0.1761 | 0.0301 | 4.84E-09 | 745819 | 20 | 17883531 | 4.59E-05 | 34.22824 |
| rs17812022 | T | C | 0.0958 | -0.3613 | 0.0525 | 5.65E-12 | 744814 | 20 | 19007099 | 6.36E-05 | 47.36049 |
| rs6058088 | T | G | 0.8439 | 0.2832 | 0.0417 | 1.14E-11 | 745819 | 20 | 30139886 | 6.18E-05 | 46.12254 |
| rs79384779 | T | C | 0.1512 | 0.3179 | 0.0428 | 1.08E-13 | 745820 | 20 | 31214944 | 7.40E-05 | 55.16865 |
| rs6029756 | A | G | 0.3225 | -0.2712 | 0.033 | 1.88E-16 | 744814 | 20 | 40266681 | 9.07E-05 | 67.53833 |
| rs6031431 | A | G | 0.5376 | -0.2617 | 0.0304 | 7.05E-18 | 743700 | 20 | 42795152 | 9.96E-05 | 74.10698 |
| rs2598 | A | G | 0.533 | 0.168 | 0.0303 | 2.87E-08 | 745818 | 20 | 47241618 | 4.12E-05 | 30.742 |
| rs6090907 | A | G | 0.147 | -0.3854 | 0.0425 | 1.29E-19 | 745820 | 20 | 47410231 | 0.00011 | 82.23267 |
| rs234623 | A | G | 0.5041 | -0.1804 | 0.0302 | 2.43E-09 | 741285 | 20 | 57488964 | 4.81E-05 | 35.68272 |
| rs6026744 | A | T | 0.8771 | -0.7131 | 0.0461 | 7.00E-54 | 741284 | 20 | 57742388 | 0.000323 | 239.2753 |
| rs28374392 | T | C | 0.6231 | 0.1924 | 0.0338 | 1.21E-08 | 678027 | 20 | 61189717 | 4.78E-05 | 32.40227 |
| rs6062324 | A | G | 0.2364 | -0.3294 | 0.0363 | 1.18E-19 | 738823 | 20 | 62446351 | 0.000111 | 82.34415 |
| rs6054139 | A | G | 0.606 | 0.2094 | 0.0306 | 8.23E-12 | 745818 | 20 | 6327810 | 6.28E-05 | 46.8284 |
| rs2776037 | T | C | 0.4151 | -0.1851 | 0.0309 | 2.15E-09 | 743701 | 21 | 16317933 | 4.82E-05 | 35.88349 |
| rs1882961 | T | C | 0.3087 | 0.2443 | 0.0326 | 6.69E-14 | 745820 | 21 | 16556367 | 7.53E-05 | 56.15786 |
| rs2833834 | A | C | 0.2765 | 0.2177 | 0.0338 | 1.22E-10 | 737558 | 21 | 33814378 | 5.62E-05 | 41.48416 |
| rs12627651 | A | G | 0.2872 | 0.3498 | 0.0341 | 1.02E-24 | 741589 | 21 | 44760603 | 0.000142 | 105.2276 |
| rs34487963 | A | C | 0.0185 | -0.8819 | 0.1244 | 1.35E-12 | 716018 | 21 | 44838330 | 7.02E-05 | 50.25702 |
| rs7278003 | T | C | 0.4378 | -0.1876 | 0.0304 | 6.63E-10 | 744814 | 21 | 44966069 | 5.11E-05 | 38.08179 |
| rs2238787 | A | G | 0.292 | 0.2552 | 0.0332 | 1.45E-14 | 743706 | 22 | 19976406 | 7.94E-05 | 59.08592 |
| rs12321 | C | G | 0.4328 | -0.2292 | 0.0303 | 3.81E-14 | 745820 | 22 | 29453193 | 7.67E-05 | 57.21933 |
| rs113264678 | T | C | 0.046 | 0.4063 | 0.0727 | 2.26E-08 | 745818 | 22 | 30135079 | 4.19E-05 | 31.23372 |
| rs8142376 | T | C | 0.491 | 0.1676 | 0.03 | 2.20E-08 | 745819 | 22 | 32001037 | 4.18E-05 | 31.21076 |
| rs148140538 | T | C | 0.0808 | -0.3252 | 0.0562 | 7.39E-09 | 742190 | 22 | 50228044 | 4.51E-05 | 33.48322 |
| rs28578714 | T | C | 0.6062 | 0.2066 | 0.0327 | 2.53E-10 | 713093 | 22 | 50727921 | 5.60E-05 | 39.91755 |
| rs10207726 | T | C | 0.296 | -0.2142 | 0.033 | 8.06E-11 | 738169 | 2 | 1.13E+08 | 5.71E-05 | 42.13179 |
| rs6737318 | A | G | 0.7782 | 0.2348 | 0.0364 | 1.13E-10 | 738168 | 2 | 1.14E+08 | 5.64E-05 | 41.60948 |
| rs2580350 | A | G | 0.5609 | 0.1769 | 0.0307 | 8.39E-09 | 737163 | 2 | 1.22E+08 | 4.50E-05 | 33.20303 |
| rs17257081 | A | G | 0.8065 | 0.2274 | 0.0392 | 6.35E-09 | 722234 | 2 | 1.36E+08 | 4.66E-05 | 33.65174 |
| rs55944332 | A | G | 0.7632 | -0.2613 | 0.0355 | 1.79E-13 | 738168 | 2 | 1.46E+08 | 7.34E-05 | 54.17775 |
| rs62170470 | T | C | 0.6017 | 0.1972 | 0.0321 | 7.69E-10 | 728446 | 2 | 1.47E+08 | 5.18E-05 | 37.74006 |
| rs62187653 | T | C | 0.9029 | 0.3286 | 0.0511 | 1.23E-10 | 738168 | 2 | 1.62E+08 | 5.60E-05 | 41.35158 |
| rs4667454 | A | G | 0.6705 | 0.2636 | 0.0322 | 2.63E-16 | 738169 | 2 | 1.65E+08 | 9.08E-05 | 67.01591 |
| rs73029563 | C | G | 0.4549 | -0.514 | 0.0304 | 4.20E-64 | 737165 | 2 | 1.65E+08 | 0.000388 | 285.8761 |
| rs10048760 | T | G | 0.5288 | -0.1862 | 0.0301 | 6.56E-10 | 738170 | 2 | 1.75E+08 | 5.18E-05 | 38.26707 |
| rs71421551 | C | G | 0.2885 | 0.2374 | 0.0333 | 1.05E-12 | 737163 | 2 | 1.77E+08 | 6.89E-05 | 50.82434 |
| rs17610485 | A | T | 0.5761 | 0.1738 | 0.0304 | 1.06E-08 | 738169 | 2 | 1.78E+08 | 4.43E-05 | 32.6852 |
| rs4894132 | T | C | 0.7283 | 0.2469 | 0.0342 | 5.51E-13 | 737164 | 2 | 1.81E+08 | 7.07E-05 | 52.11813 |
| rs12473915 | A | G | 0.2017 | -0.295 | 0.0375 | 3.42E-15 | 738169 | 2 | 1.83E+08 | 8.38E-05 | 61.88428 |
| rs13412750 | A | G | 0.2708 | -0.2889 | 0.0341 | 2.33E-17 | 729907 | 2 | 1.92E+08 | 9.83E-05 | 71.77697 |
| rs17760259 | T | C | 0.5724 | -0.2654 | 0.0304 | 2.25E-18 | 738170 | 2 | 19744462 | 0.000103 | 76.21729 |
| rs12693982 | T | C | 0.4024 | 0.2575 | 0.0309 | 7.49E-17 | 735106 | 2 | 2.04E+08 | 9.45E-05 | 69.44426 |
| rs3845811 | C | G | 0.5661 | -0.2942 | 0.0309 | 1.88E-21 | 737165 | 2 | 2.09E+08 | 0.000123 | 90.64987 |
| rs12694277 | T | C | 0.2946 | -0.2018 | 0.0335 | 1.80E-09 | 738169 | 2 | 2.13E+08 | 4.92E-05 | 36.28704 |
| rs2161967 | T | G | 0.4279 | 0.2836 | 0.0307 | 2.87E-20 | 738169 | 2 | 2.19E+08 | 0.000116 | 85.33644 |
| rs3828282 | C | G | 0.4279 | 0.1857 | 0.0318 | 5.29E-09 | 737165 | 2 | 2.19E+08 | 4.63E-05 | 34.1011 |
| rs10804330 | T | C | 0.5668 | 0.2351 | 0.0306 | 1.62E-14 | 736111 | 2 | 2.27E+08 | 8.02E-05 | 59.02843 |
| rs1044822 | T | C | 0.1482 | -0.248 | 0.0424 | 5.16E-09 | 738169 | 2 | 2.31E+08 | 4.63E-05 | 34.21137 |
| rs3754944 | A | C | 0.5875 | 0.1768 | 0.0308 | 9.30E-09 | 729906 | 2 | 2.31E+08 | 4.51E-05 | 32.95049 |
| rs139354822 | T | C | 0.9704 | 0.6115 | 0.0975 | 3.51E-10 | 720286 | 2 | 2.42E+08 | 5.46E-05 | 39.3353 |
| rs2384063 | T | C | 0.7607 | 0.3266 | 0.0357 | 6.33E-20 | 737164 | 2 | 25187115 | 0.000114 | 83.69408 |
| rs1275988 | T | C | 0.6112 | -0.541 | 0.0308 | 4.42E-69 | 738170 | 2 | 26914364 | 0.000418 | 308.5261 |
| rs13420463 | A | G | 0.7734 | 0.3143 | 0.036 | 2.72E-18 | 729450 | 2 | 37517566 | 0.000104 | 76.22239 |
| rs4952609 | A | G | 0.7439 | 0.2124 | 0.0347 | 9.60E-10 | 737163 | 2 | 40555733 | 5.08E-05 | 37.467 |
| rs115262049 | A | T | 0.9132 | 0.5893 | 0.0552 | 1.29E-26 | 738168 | 2 | 43196694 | 0.000154 | 113.9708 |
| rs12464602 | A | G | 0.6208 | -0.2437 | 0.0315 | 1.02E-14 | 738170 | 2 | 43397614 | 8.11E-05 | 59.85339 |
| rs13016772 | T | C | 0.7651 | 0.2522 | 0.0355 | 1.23E-12 | 738168 | 2 | 55779476 | 6.84E-05 | 50.46988 |
| rs2249105 | A | G | 0.6321 | 0.2927 | 0.0313 | 7.63E-21 | 729908 | 2 | 65287896 | 0.00012 | 87.44915 |
| rs10188003 | T | C | 0.393 | 0.1883 | 0.0307 | 8.80E-10 | 737056 | 2 | 66773469 | 5.10E-05 | 37.62034 |
| rs6731373 | A | G | 0.3492 | 0.1913 | 0.0326 | 4.18E-09 | 737164 | 2 | 68503044 | 4.67E-05 | 34.43448 |
| rs6732123 | C | G | 0.4174 | -0.1737 | 0.0307 | 1.52E-08 | 738169 | 2 | 69534650 | 4.34E-05 | 32.01266 |
| rs4577304 | T | C | 0.5233 | -0.1767 | 0.0302 | 4.99E-09 | 738170 | 2 | 73403040 | 4.64E-05 | 34.23403 |
| rs72847885 | A | G | 0.663 | 0.2413 | 0.0318 | 3.08E-14 | 737055 | 2 | 86326717 | 7.81E-05 | 57.57835 |
| rs9848170 | C | G | 0.597 | 0.3231 | 0.0307 | 7.01E-26 | 738170 | 3 | 11495983 | 0.00015 | 110.7633 |
| rs12637573 | A | G | 0.4718 | -0.1731 | 0.0302 | 9.95E-09 | 737164 | 3 | 1.22E+08 | 4.46E-05 | 32.85331 |
| rs6438857 | T | C | 0.5774 | 0.2736 | 0.0305 | 3.13E-19 | 738170 | 3 | 1.25E+08 | 0.000109 | 80.46951 |
| rs9880098 | A | G | 0.3946 | 0.3081 | 0.0308 | 1.59E-23 | 738169 | 3 | 1.34E+08 | 0.000136 | 100.0647 |
| rs1199330 | A | G | 0.8824 | -0.2654 | 0.047 | 1.65E-08 | 729906 | 3 | 1.38E+08 | 4.37E-05 | 31.88636 |
| rs9876694 | T | C | 0.0584 | 0.4713 | 0.0651 | 4.64E-13 | 737055 | 3 | 1.41E+08 | 7.11E-05 | 52.41212 |
| rs11925504 | A | G | 0.5721 | -0.2901 | 0.0305 | 1.78E-21 | 737164 | 3 | 14943965 | 0.000123 | 90.46792 |
| rs4408839 | A | G | 0.7433 | -0.2301 | 0.0345 | 2.43E-11 | 738168 | 3 | 1.54E+08 | 6.03E-05 | 44.48298 |
| rs79539362 | T | C | 0.8992 | 0.4003 | 0.0504 | 2.09E-15 | 738166 | 3 | 1.55E+08 | 8.55E-05 | 63.0825 |
| rs17684859 | T | C | 0.7335 | -0.2241 | 0.034 | 4.24E-11 | 738170 | 3 | 1.58E+08 | 5.88E-05 | 43.44349 |
| rs3980686 | T | G | 0.1075 | -0.4998 | 0.0487 | 1.03E-24 | 738167 | 3 | 1.69E+08 | 0.000143 | 105.3255 |
| rs1290784 | T | C | 0.4483 | 0.4124 | 0.0303 | 2.97E-42 | 736111 | 3 | 1.69E+08 | 0.000252 | 185.2469 |
| rs2111557 | T | C | 0.4675 | 0.1764 | 0.0302 | 5.22E-09 | 738170 | 3 | 1.69E+08 | 4.62E-05 | 34.11788 |
| rs4955575 | A | C | 0.7461 | 0.2158 | 0.0348 | 5.63E-10 | 738169 | 3 | 1.7E+08 | 5.21E-05 | 38.45415 |
| rs262986 | A | G | 0.4704 | -0.2371 | 0.0305 | 7.67E-15 | 738170 | 3 | 1.83E+08 | 8.19E-05 | 60.43134 |
| rs13091418 | C | G | 0.6659 | -0.2234 | 0.0325 | 6.15E-12 | 737163 | 3 | 1.85E+08 | 6.41E-05 | 47.24963 |
| rs9869437 | A | C | 0.3523 | -0.2001 | 0.0318 | 3.22E-10 | 737165 | 3 | 1.96E+08 | 5.37E-05 | 39.59486 |
| rs189267552 | A | T | 0.0132 | -0.8664 | 0.139 | 4.55E-10 | 735474 | 3 | 20073193 | 5.28E-05 | 38.85135 |
| rs2643826 | T | C | 0.4505 | 0.4473 | 0.0306 | 1.74E-48 | 738169 | 3 | 27562988 | 0.000289 | 213.675 |
| rs68115553 | A | G | 0.9801 | -0.6445 | 0.1143 | 1.74E-08 | 727329 | 3 | 27704702 | 4.37E-05 | 31.79451 |
| rs743395 | T | C | 0.3834 | 0.2597 | 0.0317 | 2.55E-16 | 737165 | 3 | 37598382 | 9.10E-05 | 67.11571 |
| rs6788984 | A | G | 0.8563 | 0.2999 | 0.0432 | 3.81E-12 | 738169 | 3 | 41107173 | 6.53E-05 | 48.19303 |
| rs1052501 | T | C | 0.8329 | 0.2262 | 0.0412 | 4.14E-08 | 729908 | 3 | 41925398 | 4.13E-05 | 30.14322 |
| rs6771917 | T | C | 0.2477 | -0.3793 | 0.0355 | 1.39E-26 | 738168 | 3 | 48108442 | 0.000155 | 114.1584 |
| rs7615099 | A | G | 0.6675 | 0.1891 | 0.0321 | 3.90E-09 | 737163 | 3 | 53143901 | 4.71E-05 | 34.70338 |
| rs6445583 | A | G | 0.7465 | 0.2774 | 0.0349 | 1.90E-15 | 738167 | 3 | 53562894 | 8.56E-05 | 63.17727 |
| rs3772219 | A | C | 0.6824 | 0.2733 | 0.0324 | 3.10E-17 | 738170 | 3 | 56771251 | 9.64E-05 | 71.15216 |
| rs7618284 | C | G | 0.3394 | -0.1891 | 0.0331 | 1.10E-08 | 737164 | 3 | 66422246 | 4.43E-05 | 32.63818 |
| rs4499560 | A | T | 0.3171 | -0.2199 | 0.0326 | 1.46E-11 | 737162 | 3 | 70920485 | 6.17E-05 | 45.50028 |
| rs1375564 | T | C | 0.6395 | 0.2579 | 0.0315 | 2.84E-16 | 736109 | 3 | 85656311 | 9.11E-05 | 67.03173 |
| rs13107325 | T | C | 0.0739 | -0.9086 | 0.0592 | 4.22E-53 | 735152 | 4 | 1.03E+08 | 0.00032 | 235.5596 |
| rs11097909 | T | C | 0.1472 | -0.3628 | 0.043 | 3.35E-17 | 735150 | 4 | 1.07E+08 | 9.68E-05 | 71.18631 |
| rs1493132 | T | C | 0.6603 | -0.1766 | 0.0318 | 2.73E-08 | 735150 | 4 | 1.09E+08 | 4.20E-05 | 30.84082 |
| rs1814951 | A | G | 0.8785 | -0.3231 | 0.0466 | 3.91E-12 | 735150 | 4 | 1.11E+08 | 6.54E-05 | 48.07296 |
| rs4834792 | A | T | 0.4796 | 0.1973 | 0.0303 | 7.24E-11 | 735152 | 4 | 1.21E+08 | 5.77E-05 | 42.40018 |
| rs7439567 | T | C | 0.4106 | 0.2537 | 0.0309 | 2.31E-16 | 734147 | 4 | 1.38E+08 | 9.18E-05 | 67.40976 |
| rs72719160 | A | T | 0.6829 | -0.2243 | 0.0324 | 4.34E-12 | 735151 | 4 | 1.44E+08 | 6.52E-05 | 47.92558 |
| rs2353940 | T | C | 0.7507 | -0.2075 | 0.0358 | 6.85E-09 | 732820 | 4 | 1.46E+08 | 4.58E-05 | 33.59456 |
| rs73855810 | A | G | 0.1406 | 0.2732 | 0.0434 | 3.04E-10 | 738169 | 4 | 1.48E+08 | 5.37E-05 | 39.62605 |
| rs7683728 | T | C | 0.5312 | -0.3654 | 0.0304 | 2.43E-33 | 728337 | 4 | 1.56E+08 | 0.000198 | 144.4737 |
| rs12643599 | A | G | 0.6395 | 0.3134 | 0.0313 | 1.23E-23 | 738170 | 4 | 1.57E+08 | 0.000136 | 100.2555 |
| rs17035181 | T | G | 0.8552 | 0.3074 | 0.0429 | 7.61E-13 | 737055 | 4 | 1.58E+08 | 6.97E-05 | 51.34427 |
| rs869396 | A | C | 0.4659 | -0.2115 | 0.0305 | 4.12E-12 | 737165 | 4 | 1.7E+08 | 6.52E-05 | 48.08614 |
| rs2610990 | A | G | 0.2641 | -0.2903 | 0.0343 | 2.86E-17 | 735152 | 4 | 18008232 | 9.74E-05 | 71.6316 |
| rs34535756 | T | C | 0.0394 | 0.478 | 0.0786 | 1.18E-09 | 737053 | 4 | 2246927 | 5.02E-05 | 36.98363 |
| rs1290933 | A | C | 0.6919 | -0.2847 | 0.0327 | 3.17E-18 | 738170 | 4 | 2668217 | 0.000103 | 75.80158 |
| rs55924432 | T | C | 0.401 | 0.2651 | 0.0317 | 5.70E-17 | 734146 | 4 | 26812737 | 9.53E-05 | 69.93583 |
| rs2498323 | A | G | 0.098 | 0.3171 | 0.0517 | 8.52E-10 | 736057 | 4 | 3451109 | 5.11E-05 | 37.61926 |
| rs2291434 | T | G | 0.5335 | -0.2622 | 0.0303 | 5.10E-18 | 735151 | 4 | 38387244 | 0.000102 | 74.88226 |
| rs12511987 | T | G | 0.8226 | -0.2329 | 0.0399 | 5.39E-09 | 738169 | 4 | 46595623 | 4.62E-05 | 34.07156 |
| rs62309747 | A | G | 0.4734 | -0.2244 | 0.0304 | 1.59E-13 | 737165 | 4 | 48713862 | 7.39E-05 | 54.48756 |
| rs60991988 | T | G | 0.8931 | 0.3789 | 0.0498 | 2.82E-14 | 729449 | 4 | 54801228 | 7.94E-05 | 57.88811 |
| rs13107261 | A | G | 0.3687 | -0.1778 | 0.0314 | 1.57E-08 | 729906 | 4 | 63768826 | 4.39E-05 | 32.06292 |
| rs5020545 | T | C | 0.4437 | -0.2179 | 0.0305 | 9.71E-13 | 737164 | 4 | 77414988 | 6.92E-05 | 51.04035 |
| rs12509595 | T | C | 0.7077 | -0.8367 | 0.0334 | 2.55E-138 | 737164 | 4 | 81182554 | 0.000851 | 627.5458 |
| rs6823199 | T | C | 0.7438 | 0.2094 | 0.0348 | 1.72E-09 | 732535 | 4 | 83925895 | 4.94E-05 | 36.20709 |
| rs17010957 | T | C | 0.8537 | -0.534 | 0.043 | 1.78E-35 | 735152 | 4 | 86719165 | 0.00021 | 154.2213 |
| rs13149209 | T | C | 0.7773 | 0.281 | 0.0367 | 1.97E-14 | 735149 | 4 | 89750668 | 7.97E-05 | 58.62452 |
| rs11241313 | T | C | 0.3112 | -0.2071 | 0.0326 | 2.23E-10 | 738169 | 5 | 1.14E+08 | 5.47E-05 | 40.35746 |
| rs1624823 | A | G | 0.3801 | 0.3371 | 0.0313 | 4.26E-27 | 738170 | 5 | 1.22E+08 | 0.000157 | 115.9919 |
| rs9327297 | C | G | 0.6676 | 0.2747 | 0.0319 | 8.07E-18 | 738169 | 5 | 1.23E+08 | 0.0001 | 74.15403 |
| rs758179 | C | G | 0.7756 | -0.2091 | 0.0367 | 1.19E-08 | 737164 | 5 | 1.27E+08 | 4.40E-05 | 32.46196 |
| rs6892983 | A | C | 0.4022 | 0.3427 | 0.0307 | 7.11E-29 | 737164 | 5 | 1.28E+08 | 0.000169 | 124.6092 |
| rs10069690 | T | C | 0.2582 | 0.3098 | 0.0369 | 4.47E-17 | 707524 | 5 | 1279790 | 9.96E-05 | 70.48697 |
| rs702395 | T | C | 0.4369 | 0.2318 | 0.0305 | 3.24E-14 | 737165 | 5 | 1.4E+08 | 7.83E-05 | 57.75984 |
| rs2913920 | T | C | 0.765 | 0.2418 | 0.0359 | 1.62E-11 | 735150 | 5 | 1.42E+08 | 6.17E-05 | 45.36517 |
| rs7725413 | T | C | 0.7699 | -0.1985 | 0.0359 | 3.07E-08 | 737054 | 5 | 15695987 | 4.15E-05 | 30.5725 |
| rs1957563 | T | C | 0.265 | 0.3629 | 0.0342 | 2.32E-26 | 735150 | 5 | 1.57E+08 | 0.000153 | 112.5954 |
| rs11960210 | T | C | 0.6245 | 0.4727 | 0.0313 | 1.25E-51 | 726890 | 5 | 1.58E+08 | 0.000314 | 228.0769 |
| rs13358657 | A | G | 0.8668 | -0.388 | 0.0445 | 2.95E-18 | 735151 | 5 | 1.58E+08 | 0.000103 | 76.02252 |
| rs3860770 | A | G | 0.2916 | -0.2663 | 0.0333 | 1.20E-15 | 733093 | 5 | 1.73E+08 | 8.72E-05 | 63.95179 |
| rs12153395 | A | G | 0.1147 | -0.3303 | 0.0486 | 1.07E-11 | 734145 | 5 | 1.79E+08 | 6.29E-05 | 46.18952 |
| rs12656497 | T | C | 0.4034 | -0.6382 | 0.0307 | 7.14E-96 | 736111 | 5 | 32831939 | 0.000587 | 432.1511 |
| rs10941043 | T | G | 0.7098 | -0.2585 | 0.0332 | 6.42E-15 | 738168 | 5 | 33194751 | 8.21E-05 | 60.62388 |
| rs4957026 | A | G | 0.3399 | 0.1982 | 0.0323 | 8.12E-10 | 735051 | 5 | 361148 | 5.12E-05 | 37.65313 |
| rs2113077 | A | G | 0.4303 | 0.2097 | 0.0305 | 6.09E-12 | 737056 | 5 | 50799442 | 6.41E-05 | 47.27113 |
| rs1694068 | A | T | 0.6139 | 0.2657 | 0.0311 | 1.18E-17 | 738169 | 5 | 53283630 | 9.89E-05 | 72.98963 |
| rs10043077 | T | C | 0.639 | -0.1931 | 0.0324 | 2.52E-09 | 737163 | 5 | 55692939 | 4.82E-05 | 35.52003 |
| rs34496659 | A | G | 0.0702 | 0.4545 | 0.0616 | 1.54E-13 | 738167 | 5 | 61798934 | 7.37E-05 | 54.43838 |
| rs6870654 | T | C | 0.7454 | 0.2136 | 0.0347 | 7.58E-10 | 729908 | 5 | 63831964 | 5.19E-05 | 37.89155 |
| rs4286632 | A | G | 0.7306 | 0.211 | 0.0343 | 7.64E-10 | 738169 | 5 | 66291370 | 5.13E-05 | 37.84212 |
| rs7703560 | A | G | 0.7002 | -0.2246 | 0.0333 | 1.51E-11 | 729449 | 5 | 67678506 | 6.24E-05 | 45.49146 |
| rs246973 | T | C | 0.2882 | 0.2479 | 0.0335 | 1.45E-13 | 737164 | 5 | 68007803 | 7.43E-05 | 54.75985 |
| rs6452769 | A | G | 0.2053 | -0.3143 | 0.0377 | 7.82E-17 | 737165 | 5 | 87389027 | 9.43E-05 | 69.50321 |
| rs76443575 | C | G | 0.0359 | -0.5233 | 0.0816 | 1.40E-10 | 737711 | 5 | 96211594 | 5.57E-05 | 41.12633 |
| rs1871190 | T | G | 0.3349 | 0.1954 | 0.0324 | 1.66E-09 | 737164 | 5 | 97953719 | 4.93E-05 | 36.37122 |
| rs9486916 | T | C | 0.1979 | 0.2657 | 0.0385 | 5.42E-12 | 729449 | 6 | 1.09E+08 | 6.53E-05 | 47.62779 |
| rs961764 | C | G | 0.4254 | -0.1909 | 0.0305 | 3.75E-10 | 738170 | 6 | 1.18E+08 | 5.31E-05 | 39.17518 |
| rs1630736 | T | C | 0.465 | -0.1706 | 0.0309 | 3.52E-08 | 737165 | 6 | 12295987 | 4.13E-05 | 30.48175 |
| rs10782230 | A | G | 0.4845 | 0.2106 | 0.0302 | 2.91E-12 | 738169 | 6 | 1.26E+08 | 6.59E-05 | 48.62971 |
| rs9401913 | A | G | 0.4387 | 0.5202 | 0.0305 | 3.66E-65 | 738170 | 6 | 1.27E+08 | 0.000394 | 290.8974 |
| rs9349379 | A | G | 0.593 | 0.2664 | 0.0312 | 1.31E-17 | 737164 | 6 | 12903957 | 9.89E-05 | 72.90513 |
| rs2327429 | T | C | 0.7083 | 0.2 | 0.0338 | 3.16E-09 | 737164 | 6 | 1.34E+08 | 4.75E-05 | 35.01268 |
| rs8180684 | T | C | 0.2896 | 0.2134 | 0.0335 | 1.80E-10 | 737164 | 6 | 1.43E+08 | 5.50E-05 | 40.57869 |
| rs7765526 | A | G | 0.4633 | 0.201 | 0.0307 | 5.88E-11 | 737165 | 6 | 1.48E+08 | 5.81E-05 | 42.86612 |
| rs17080102 | C | G | 0.0694 | -0.8085 | 0.0594 | 3.52E-42 | 738169 | 6 | 1.51E+08 | 0.000251 | 185.2618 |
| rs1293969 | T | C | 0.7484 | -0.1988 | 0.0347 | 1.03E-08 | 738169 | 6 | 1.52E+08 | 4.45E-05 | 32.82257 |
| rs509833 | A | G | 0.1386 | 0.329 | 0.044 | 7.08E-14 | 737164 | 6 | 1.6E+08 | 7.58E-05 | 55.90946 |
| rs2745599 | A | G | 0.552 | 0.2164 | 0.0317 | 8.96E-12 | 728445 | 6 | 1613686 | 6.40E-05 | 46.60095 |
| rs12661036 | T | C | 0.775 | -0.2104 | 0.0374 | 1.82E-08 | 737165 | 6 | 1.64E+08 | 4.29E-05 | 31.64806 |
| rs7744902 | A | G | 0.0766 | -0.4088 | 0.0593 | 5.64E-12 | 713753 | 6 | 1.66E+08 | 6.66E-05 | 47.5238 |
| rs9368222 | A | C | 0.2688 | 0.2281 | 0.0339 | 1.84E-11 | 738169 | 6 | 20686996 | 6.13E-05 | 45.27412 |
| rs9393231 | A | C | 0.4924 | -0.2148 | 0.0309 | 3.39E-12 | 737164 | 6 | 22123695 | 6.55E-05 | 48.32261 |
| rs7753826 | A | T | 0.1899 | 0.4276 | 0.0385 | 9.96E-29 | 738170 | 6 | 26042239 | 0.000167 | 123.3539 |
| rs2596498 | T | C | 0.6379 | -0.233 | 0.0337 | 4.90E-12 | 692081 | 6 | 31322688 | 6.91E-05 | 47.80252 |
| rs3132442 | T | C | 0.5199 | 0.3931 | 0.0304 | 2.65E-38 | 727903 | 6 | 31839494 | 0.00023 | 167.2083 |
| rs7763558 | A | G | 0.3241 | 0.3363 | 0.0321 | 1.17E-25 | 738170 | 6 | 43349215 | 0.000149 | 109.7596 |
| rs11967262 | C | G | 0.5132 | -0.1715 | 0.0311 | 3.43E-08 | 730376 | 6 | 43760327 | 4.16E-05 | 30.40929 |
| rs78648104 | T | C | 0.9075 | -0.4287 | 0.0541 | 2.37E-15 | 735105 | 6 | 50683009 | 8.54E-05 | 62.793 |
| rs1575290 | T | C | 0.4733 | 0.1973 | 0.0301 | 5.59E-11 | 738170 | 6 | 7715689 | 5.82E-05 | 42.96551 |
| rs1984195 | A | G | 0.4887 | 0.2409 | 0.0303 | 1.77E-15 | 729908 | 6 | 79657391 | 8.66E-05 | 63.2102 |
| rs9361836 | T | C | 0.3172 | 0.2196 | 0.0324 | 1.25E-11 | 738170 | 6 | 82235408 | 6.22E-05 | 45.93815 |
| rs6921291 | T | C | 0.1907 | 0.3575 | 0.0385 | 1.58E-20 | 738169 | 6 | 97066242 | 0.000117 | 86.22426 |
| rs2392929 | T | G | 0.7973 | -0.7507 | 0.0379 | 1.96E-87 | 737165 | 7 | 1.06E+08 | 0.000532 | 392.3315 |
| rs34072724 | A | G | 0.4889 | -0.2422 | 0.0303 | 1.37E-15 | 736111 | 7 | 1.3E+08 | 8.68E-05 | 63.89426 |
| rs35680304 | T | C | 0.5929 | 0.2694 | 0.031 | 3.76E-18 | 736051 | 7 | 1.31E+08 | 0.000103 | 75.5215 |
| rs75672964 | T | C | 0.0418 | 0.5885 | 0.0839 | 2.35E-12 | 712274 | 7 | 1.31E+08 | 6.91E-05 | 49.2003 |
| rs73727605 | A | G | 0.0663 | 0.3616 | 0.0623 | 6.60E-09 | 726466 | 7 | 1.49E+08 | 4.64E-05 | 33.68834 |
| rs3918226 | T | C | 0.0811 | 0.664 | 0.0575 | 8.46E-31 | 731379 | 7 | 1.51E+08 | 0.000182 | 133.3519 |
| rs10224210 | T | C | 0.7211 | -0.3831 | 0.034 | 1.60E-29 | 738170 | 7 | 1.51E+08 | 0.000172 | 126.9595 |
| rs1870735 | C | G | 0.4531 | 0.206 | 0.0311 | 3.61E-11 | 737163 | 7 | 1.56E+08 | 5.95E-05 | 43.87453 |
| rs3807925 | A | G | 0.6496 | -0.1859 | 0.0319 | 5.39E-09 | 736051 | 7 | 18543250 | 4.61E-05 | 33.96067 |
| rs28688791 | T | C | 0.8018 | -0.3222 | 0.038 | 2.34E-17 | 738167 | 7 | 19039605 | 9.74E-05 | 71.89235 |
| rs6959688 | A | G | 0.5981 | -0.2344 | 0.031 | 4.22E-14 | 733939 | 7 | 1966831 | 7.79E-05 | 57.17296 |
| rs112509803 | C | G | 0.1138 | -0.2641 | 0.0477 | 3.18E-08 | 738168 | 7 | 24735004 | 4.15E-05 | 30.65483 |
| rs10282122 | T | C | 0.6684 | -0.302 | 0.0327 | 2.46E-20 | 735052 | 7 | 2529623 | 0.000116 | 85.29375 |
| rs3735533 | T | C | 0.0743 | -0.91 | 0.0577 | 5.29E-56 | 737165 | 7 | 27245893 | 0.000337 | 248.731 |
| rs6961048 | C | G | 0.896 | -0.5304 | 0.0497 | 1.43E-26 | 734292 | 7 | 27328187 | 0.000155 | 113.892 |
| rs977184 | T | C | 0.6252 | -0.184 | 0.0314 | 4.86E-09 | 729451 | 7 | 28650761 | 4.71E-05 | 34.33801 |
| rs11977526 | A | G | 0.4009 | -0.3213 | 0.0312 | 6.62E-25 | 732149 | 7 | 46008110 | 0.000145 | 106.0501 |
| rs73049928 | A | G | 0.8061 | -0.2382 | 0.0392 | 1.20E-09 | 737053 | 7 | 4669949 | 5.01E-05 | 36.92412 |
| rs12668436 | T | C | 0.7541 | -0.2151 | 0.035 | 7.88E-10 | 738168 | 7 | 47548893 | 5.12E-05 | 37.7697 |
| rs848445 | T | C | 0.2851 | -0.2025 | 0.0339 | 2.28E-09 | 738169 | 7 | 77572461 | 4.83E-05 | 35.68202 |
| rs42377 | A | G | 0.3045 | -0.3153 | 0.0331 | 1.69E-21 | 726789 | 7 | 92243672 | 0.000125 | 90.73833 |
| rs79069610 | T | C | 0.95 | -0.4005 | 0.0727 | 3.68E-08 | 738168 | 8 | 1.06E+08 | 4.11E-05 | 30.34835 |
| rs35783704 | A | G | 0.1042 | -0.4619 | 0.0507 | 8.81E-20 | 737055 | 8 | 1.06E+08 | 0.000113 | 83.00014 |
| rs1821002 | C | G | 0.4108 | 0.3794 | 0.0307 | 5.19E-35 | 738169 | 8 | 10640065 | 0.000207 | 152.7273 |
| rs7830607 | A | G | 0.3046 | -0.206 | 0.0327 | 3.09E-10 | 738170 | 8 | 1.1E+08 | 5.38E-05 | 39.68604 |
| rs2470004 | T | C | 0.8175 | -0.3454 | 0.0392 | 1.28E-18 | 738168 | 8 | 1.2E+08 | 0.000105 | 77.63747 |
| rs4598218 | T | C | 0.6158 | 0.1911 | 0.0313 | 1.00E-09 | 728337 | 8 | 1.29E+08 | 5.12E-05 | 37.27619 |
| rs7012866 | T | G | 0.4991 | -0.2325 | 0.0301 | 1.21E-14 | 737165 | 8 | 1.36E+08 | 8.09E-05 | 59.66391 |
| rs4440615 | A | G | 0.6321 | -0.2201 | 0.0312 | 1.87E-12 | 738170 | 8 | 1.41E+08 | 6.74E-05 | 49.76565 |
| rs4961293 | T | C | 0.4513 | 0.2268 | 0.0303 | 7.35E-14 | 738169 | 8 | 1.42E+08 | 7.59E-05 | 56.0273 |
| rs7463212 | A | T | 0.5445 | -0.2753 | 0.0305 | 1.81E-19 | 728903 | 8 | 1.44E+08 | 0.000112 | 81.4726 |
| rs71499040 | C | G | 0.7076 | 0.2215 | 0.0338 | 5.63E-11 | 732671 | 8 | 1711918 | 5.86E-05 | 42.94503 |
| rs10866828 | T | C | 0.2496 | 0.2476 | 0.0355 | 3.19E-12 | 729449 | 8 | 23401534 | 6.67E-05 | 48.64558 |
| rs7821832 | T | G | 0.7447 | 0.4222 | 0.0348 | 6.67E-34 | 729908 | 8 | 25889446 | 0.000202 | 147.1895 |
| rs77375686 | A | G | 0.8883 | -0.3467 | 0.0485 | 8.38E-13 | 738166 | 8 | 26043622 | 6.92E-05 | 51.10025 |
| rs1906672 | A | G | 0.2319 | 0.2966 | 0.0358 | 1.20E-16 | 738169 | 8 | 38130025 | 9.30E-05 | 68.63965 |
| rs4873492 | T | C | 0.1724 | 0.3431 | 0.0403 | 1.61E-17 | 738170 | 8 | 51947549 | 9.82E-05 | 72.482 |
| rs2354862 | A | C | 0.6407 | 0.2507 | 0.0317 | 2.42E-15 | 729451 | 8 | 64501744 | 8.57E-05 | 62.54448 |
| rs13253358 | T | C | 0.2979 | 0.2127 | 0.033 | 1.13E-10 | 738169 | 8 | 68920135 | 5.63E-05 | 41.54377 |
| rs2126474 | T | G | 0.4125 | -0.2601 | 0.0306 | 1.87E-17 | 737054 | 8 | 76878957 | 9.80E-05 | 72.2498 |
| rs9918879 | T | G | 0.103 | -0.2984 | 0.0499 | 2.28E-09 | 738169 | 8 | 77681093 | 4.84E-05 | 35.75982 |
| rs148401029 | A | C | 0.0352 | -0.4623 | 0.0848 | 4.97E-08 | 738168 | 8 | 81386066 | 4.03E-05 | 29.72042 |
| rs10091532 | A | C | 0.4168 | -0.2067 | 0.0305 | 1.33E-11 | 738170 | 8 | 82853793 | 6.22E-05 | 45.92827 |
| rs843093 | A | G | 0.7088 | -0.2085 | 0.0338 | 6.95E-10 | 737165 | 8 | 92528310 | 5.16E-05 | 38.052 |
| rs2613203 | A | T | 0.8148 | -0.2681 | 0.0389 | 5.81E-12 | 738168 | 8 | 95253197 | 6.43E-05 | 47.49996 |
| rs10980408 | T | C | 0.9641 | -0.7606 | 0.0827 | 3.83E-20 | 745817 | 9 | 1.13E+08 | 0.000113 | 84.58638 |
| rs2900568 | T | C | 0.5184 | -0.1889 | 0.03 | 2.96E-10 | 745820 | 9 | 1.17E+08 | 5.32E-05 | 39.6479 |
| rs34025993 | A | G | 0.414 | 0.223 | 0.0308 | 4.71E-13 | 744814 | 9 | 1.24E+08 | 7.04E-05 | 52.42122 |
| rs7854147 | A | G | 0.877 | 0.3056 | 0.0461 | 3.29E-11 | 737099 | 9 | 1.26E+08 | 5.96E-05 | 43.94441 |
| rs13289468 | A | C | 0.5743 | 0.2488 | 0.0306 | 3.93E-16 | 744815 | 9 | 1.28E+08 | 8.88E-05 | 66.10841 |
| rs6271 | T | C | 0.0735 | -0.5547 | 0.0611 | 1.18E-19 | 736797 | 9 | 1.37E+08 | 0.000112 | 82.42002 |
| rs11145807 | A | G | 0.4057 | 0.2135 | 0.0322 | 3.54E-11 | 721505 | 9 | 1.4E+08 | 6.09E-05 | 43.96254 |
| rs9886665 | T | C | 0.2671 | 0.2048 | 0.0343 | 2.47E-09 | 736095 | 9 | 22942770 | 4.84E-05 | 35.6509 |
| rs4553000 | T | C | 0.5141 | -0.2035 | 0.03 | 1.09E-11 | 745820 | 9 | 34223553 | 6.17E-05 | 46.01349 |
| rs76452347 | T | C | 0.205 | -0.2974 | 0.0397 | 7.13E-14 | 743700 | 9 | 35906471 | 7.55E-05 | 56.11768 |
| rs927315 | T | C | 0.4713 | 0.1689 | 0.0303 | 2.44E-08 | 744705 | 9 | 4117713 | 4.17E-05 | 31.07226 |
| rs60191654 | A | G | 0.8118 | -0.2382 | 0.0385 | 5.88E-10 | 745818 | 9 | 753648 | 5.13E-05 | 38.27903 |
| rs10746963 | A | G | 0.1834 | -0.2177 | 0.0388 | 2.05E-08 | 745820 | 9 | 77238558 | 4.22E-05 | 31.48127 |
| rs1332813 | T | C | 0.3514 | 0.2203 | 0.0314 | 2.32E-12 | 745819 | 9 | 9350706 | 6.60E-05 | 49.22305 |
| rs7045409 | A | T | 0.3669 | -0.1862 | 0.0313 | 2.55E-09 | 741943 | 9 | 95201540 | 4.77E-05 | 35.3891 |

Table S4. Genetic proxies identified for each antihypertensive drug class.

AGTi, angiotensinogen inhibitor; ERA, endothelin receptor antagonist; PDE5 inhibitor, phosphodiesterase-5 inhibitor; sGC stimulator, soluble guanylate cyclase stimulator. Chr, chromosom; pos, position; sbp_ea, effect allele for systolic blood pressure; sbp_nea, non-effect allele; sbp_eaf, effect allele frequency; sbp_b, beta estimate; sbp_se, standard error; sbp_p, p-value; sbp_n , sample size; F_stat, F statistics.

| **Drug class** | **Target gene** | **SNP** | **chr** | **pos** | **sbp_ea** | **sbp_nea** | **sbp_eaf** | | **sbp_b** | **sbp_se** | **sbp_p** | **sbp_n** | **R^2^** | **F_stat** |
| --- | --- | --- | --- | --- | --- | --- | --- | --- | --- | --- | --- | --- | --- | --- |
| AGTi | *AGT* | rs61751101 | 1 | 230836734 | A | G | 0.93 | -0.404 | | 0.061 | 2.73E-11 | 738168 | 0.0001 | 44.356 |
|  | *AGT* | rs699 | 1 | 230845794 | A | G | 0.593 | -0.375 | | 0.031 | 5.59E-34 | 721189 | 0.0002 | 148.08 |
| ERA | *EDNRA* | rs13143677 | 4 | 148419040 | A | G | 0.293 | 0.224 | | 0.033 | 1.82E-11 | 738169 | 0.0001 | 45.128 |
| PDE5 inhibitor | *PDE5A* | rs4834792 | 4 | 120555696 | A | T | 0.48 | 0.197 | | 0.03 | 7.24E-11 | 735152 | 0.0001 | 42.4 |
|  | *NOS3* | rs740956 | 7 | 150598440 | T | C | 0.567 | 0.2 | | 0.031 | 5.21E-11 | 736110 | 0.0001 | 43.042 |
|  | *NOS3* | rs3918226 | 7 | 150690176 | T | C | 0.081 | 0.664 | | 0.058 | 8.46E-31 | 731379 | 0.0002 | 133.352 |
|  | *NOS3* | rs891511 | 7 | 150704843 | A | G | 0.334 | -0.351 | | 0.033 | 6.13E-26 | 735050 | 0.0002 | 110.85 |
|  | *NOS3* | rs34581141 | 7 | 150810073 | A | G | 0.684 | -0.188 | | 0.033 | 9.08E-09 | 738169 | 0.0000 | 33.018 |
| sGC stimulator | *GUCY1A3* | rs1483040 | 4 | 156503399 | C | G | 0.094 | 0.346 | | 0.053 | 5.22E-11 | 738168 | 0.0001 | 43.055 |
|  | *GUCY1A3* | rs990619 | 4 | 156507678 | C | G | 0.476 | -0.297 | | 0.03 | 4.68E-23 | 738170 | 0.0001 | 97.622 |
|  | *GUCY1A3* | rs10010626 | 4 | 156519815 | A | T | 0.072 | 0.369 | | 0.063 | 4.12E-09 | 719168 | 0.0000 | 34.541 |
|  | *GUCY1A3* | rs28531888 | 4 | 156613960 | T | C | 0.199 | -0.226 | | 0.04 | 1.17E-08 | 737164 | 0.0000 | 32.455 |
|  | *GUCY1A3* | rs12643599 | 4 | 156639846 | A | G | 0.64 | 0.313 | | 0.031 | 1.23E-23 | 738170 | 0.0001 | 100.256 |
|  | *GUCY1A3* | rs78975989 | 4 | 156718205 | A | G | 0.109 | -0.32 | | 0.05 | 1.67E-10 | 738167 | 0.0001 | 40.771 |

Table S5. Genetic instruments information

| **Drug class** | **Target gene** | **SNP** | **chr** | **position_SNP** | **position_gene** | **SNP details** |
| --- | --- | --- | --- | --- | --- | --- |
| AGTi | *AGT* | rs699 | 1 | 230,845,794 | chr1:230,838,269-230,881,329 | missense variant |
|  | *AGT* | rs61751101 | 1 | 230,836,734 | chr1:230,838,269-230,881,329 | intergenic variant |
| ERA | *EDNRA* | rs13143677 | 4 | 148,419,040 | chr4:148,402,249-148,466,106 | intron variant |
| PDE5 inhibitor | *PDE5A* | rs4834792 | 4 | 120,555,696 | chr4:120,415,558-120,549,959 | intergenic variant |
|  | *NOS3* | rs740956 | 7 | 150,598,440 | chr7:150,688,105-150,711,676 | regulatory region variant |
|  | *NOS3* | rs3918226 | 7 | 150,690,176 | chr7:150,688,105-150,711,676 | intron variant |
|  | *NOS3* | rs891511 | 7 | 150,704,843 | chr7:150,688,105-150,711,676 | intron variant |
|  | *NOS3* | rs34581141 | 7 | 150,810,073 | chr7:150,688,105-150,711,676 | intron variant |
| sGC stimulator | *GUCY1A3* | rs1483040 | 4 | 156,503,399 | chr4:156,588,000-156,658,211 | intergenic variant |
|  | *GUCY1A3* | rs990619 | 4 | 156,507,678 | chr4:156,588,000-156,658,211 | intergenic variant |
|  | *GUCY1A3* | rs10010626 | 4 | 156,519,815 | chr4:156,588,000-156,658,211 | intergenic variant |
|  | *GUCY1A3* | rs28531888 | 4 | 156,613,960 | chr4:156,588,000-156,658,211 | intron variant |
|  | *GUCY1A3* | rs12643599 | 4 | 156,639,846 | chr4:156,588,000-156,658,211 | intron variant |
|  | *GUCY1A3* | rs78975989 | 4 | 156,718,205 | chr4:156,588,000-156,658,211 | intron variant |

Table S6. MR estimates of the effect of genetically predicted systolic blood pressure on main outcomes.

SBP, systolic blood pressure; CAD indicates coronary artery disease; MI, myocardial infarction; AF, atrial fibrillation; HF, heart failure; CKD, chronic kidney disease; T2D, type 2 diabetes; IVW, inverse-variant weighted method; nSNPs, number of single nucleotide polymorphisms; OR, odds ratio per 10 mmHg decrease in SBP; OR_lci95, lower bound of 95% confidence interval of odds ratio ; OR_uci95, upper bound of 95% confidence interval of odds ratio; pval, p-value; NA, not applicable.

| **Method** | **Exposure** | **Outcome** | **nSNPs** | **OR** | **OR_lci95** | **OR_uci95** | **p** |
| --- | --- | --- | --- | --- | --- | --- | --- |
| Simple median | SBP | AF | 454 | 0.839 | 0.805 | 0.873 | 1.57E-17 |
| Weighted median | SBP | AF | 454 | 0.833 | 0.8 | 0.868 | 2.15E-18 |
| IVW | SBP | AF | 454 | 0.851 | 0.817 | 0.887 | 9.49E-15 |
| MR-Egger | SBP | AF | 454 | 0.851 | 0.767 | 0.944 | 0.002 |
| (intercept) | SBP | AF | 454 | NA | NA | NA | 0.994 |
| Simple median | SBP | CAD | 454 | 0.714 | 0.691 | 0.739 | 4.85E-87 |
| Weighted median | SBP | CAD | 454 | 0.704 | 0.681 | 0.729 | 3.46E-90 |
| IVW | SBP | CAD | 454 | 0.711 | 0.678 | 0.745 | 8.14E-47 |
| MR-Egger | SBP | CAD | 454 | 0.715 | 0.635 | 0.806 | 3.44E-08 |
| (intercept) | SBP | CAD | 454 | NA | NA | NA | 0.912 |
| Simple median | SBP | MI | 454 | 0.738 | 0.693 | 0.786 | 1.89E-21 |
| Weighted median | SBP | MI | 454 | 0.725 | 0.68 | 0.773 | 5.94E-23 |
| IVW | SBP | MI | 454 | 0.745 | 0.704 | 0.789 | 5.00E-24 |
| MR-Egger | SBP | MI | 454 | 0.732 | 0.633 | 0.846 | 2.51E-05 |
| (intercept) | SBP | MI | 454 | NA | NA | NA | 0.793 |
| Simple median | SBP | HF | 454 | 0.79 | 0.754 | 0.828 | 2.96E-23 |
| Weighted median | SBP | HF | 454 | 0.789 | 0.752 | 0.828 | 9.58E-22 |
| IVW | SBP | HF | 454 | 0.808 | 0.777 | 0.839 | 1.53E-27 |
| MR-Egger | SBP | HF | 454 | 0.801 | 0.726 | 0.884 | 1.01E-05 |
| (intercept) | SBP | HF | 454 | NA | NA | NA | 0.865 |
| Simple median | SBP | Ischemic stroke | 454 | 0.764 | 0.73 | 0.798 | 1.12E-32 |
| Weighted median | SBP | Ischemic stroke | 454 | 0.748 | 0.716 | 0.782 | 4.57E-38 |
| IVW | SBP | Ischemic stroke | 454 | 0.754 | 0.726 | 0.782 | 2.18E-50 |
| MR-Egger | SBP | Ischemic stroke | 454 | 0.69 | 0.628 | 0.758 | 1.04E-14 |
| (intercept) | SBP | Ischemic stroke | 454 | NA | NA | NA | 0.044 |
| Simple median | SBP | CKD | 454 | 0.871 | 0.825 | 0.92 | 7.54E-07 |
| Weighted median | SBP | CKD | 454 | 0.86 | 0.814 | 0.909 | 7.83E-08 |
| IVW | SBP | CKD | 454 | 0.864 | 0.817 | 0.914 | 3.76E-07 |
| MR-Egger | SBP | CKD | 454 | 0.836 | 0.724 | 0.966 | 0.015 |
| (intercept) | SBP | CKD | 454 | NA | NA | NA | 0.626 |
| Simple median | SBP | T2D | 454 | 0.858 | 0.823 | 0.895 | 8.11E-13 |
| Weighted median | SBP | T2D | 454 | 0.896 | 0.858 | 0.935 | 4.92E-07 |
| IVW | SBP | T2D | 454 | 0.83 | 0.779 | 0.883 | 4.56E-09 |
| MR-Egger | SBP | T2D | 454 | 1.078 | 0.921 | 1.262 | 0.35 |
| (intercept) | SBP | T2D | 454 | NA | NA | NA | 0.0004 |

Table S7. MR analysis results for genetically proxied drug classes on the main outcomes.

The bold values in the p column indicate that the main IVW analysis results passed the Bonferroni-corrected threshold of 0.0014. SBP, systolic blood pressure; CAD indicates coronary artery disease; MI, myocardial infarction; AF, atrial fibrillation; HF, heart failure; CKD, chronic kidney disease; T2D, type 2 diabetes; IVW, inverse-variant weighted method; nSNP, number of single nucleotide polymorphisms; OR, odds ratio per 10 mmHg decrease in SBP; OR_lci, lower bound of 95% confidence interval of odds ratio ; OR_uci, upper bound of 95% confidence interval of odds ratio; pval, p-value; NA, not applicable; AGTi, Angiotensinogen inhibitor; ERAs, endothelin receptor antagonists; PDE5 inhibitors, phosphodiesterase type 5; sGC; soluble guanylate cyclase.

| **Drug class** | **Exposure** | **Outcome** | **Method** | **n_**  **SNP** | **OR** | **OR_**  **lci** | **OR_**  **uci** | **p** |
| --- | --- | --- | --- | --- | --- | --- | --- | --- |
| AGTi | SBP | AF | IVW | 2 | 0.758 | 0.555 | 1.035 | 0.081 |
|  |  | CAD |  | 2 | 0.647 | 0.515 | 0.814 | **0.0002** |
|  |  | MI |  | 2 | 0.468 | 0.29 | 0.755 | 0.002 |
|  |  | HF |  | 2 | 0.814 | 0.564 | 1.175 | 0.271 |
|  |  | Ischemic stroke |  | 2 | 0.487 | 0.333 | 0.712 | **0.0002** |
|  |  | CKD |  | 2 | 0.711 | 0.46 | 1.099 | 0.125 |
|  |  | T2D |  | 2 | 0.951 | 0.707 | 1.279 | 0.74 |
| ERAs | SBP | AF | IVW | 1 | 0.409 | 0.216 | 0.775 | 0.006 |
|  |  | CAD |  | 1 | 0.197 | 0.123 | 0.315 | **1.19E-11** |
|  |  | MI |  | 1 | 0.188 | 0.071 | 0.496 | **0.0007** |
|  |  | HF |  | 1 | 0.687 | 0.321 | 1.472 | 0.334 |
|  |  | Ischemic stroke |  | 1 | 0.218 | 0.107 | 0.443 | **2.56E-05** |
|  |  | CKD |  | 1 | 1.234 | 0.487 | 3.123 | 0.657 |
|  |  | T2D |  | 1 | 0.793 | 0.425 | 1.476 | 0.464 |
| PDE5 inhibitors | SBP | AF | Simple median | 5 | 0.772 | 0.57 | 1.047 | 0.096 |
|  |  |  | Weighted median | 5 | 0.785 | 0.598 | 1.032 | 0.083 |
|  |  |  | IVW | 5 | 0.766 | 0.612 | 0.958 | 0.019 |
|  |  |  | MR-Egger | 5 | 0.823 | 0.494 | 1.371 | 0.454 |
|  |  |  | (intercept) | 5 | NA | NA | NA | 0.759 |
| PDE5 inhibitors | SBP | CAD | Simple median | 5 | 0.393 | 0.241 | 0.64 | 0.0002 |
|  |  |  | Weighted median | 5 | 0.338 | 0.228 | 0.5 | 5.76E-08 |
|  |  |  | IVW | 5 | 0.348 | 0.199 | 0.607 | **0.0002** |
|  |  |  | MR-Egger | 5 | 0.197 | 0.054 | 0.714 | 0.013 |
|  |  |  | (intercept) | 5 | NA | NA | NA | 0.336 |
| PDE5 inhibitors | SBP | MI | Simple median | 5 | 0.33 | 0.162 | 0.67 | 0.002 |
|  |  |  | Weighted median | 5 | 0.33 | 0.177 | 0.618 | 0.001 |
|  |  |  | IVW | 5 | 0.381 | 0.2 | 0.728 | 0.003 |
|  |  |  | MR-Egger | 5 | 0.231 | 0.041 | 1.295 | 0.096 |
|  |  |  | (intercept) | 5 | NA | NA | NA | 0.533 |
| PDE5 inhibitors | SBP | HF | Simple median | 5 | 0.812 | 0.526 | 1.256 | 0.35 |
|  |  |  | Weighted median | 5 | 0.755 | 0.513 | 1.111 | 0.154 |
|  |  |  | IVW | 5 | 0.652 | 0.452 | 0.94 | 0.022 |
|  |  |  | MR-Egger | 5 | 0.362 | 0.199 | 0.659 | 0.001 |
|  |  |  | (intercept) | 5 | NA | NA | NA | 0.032 |
| PDE5 inhibitors | SBP | Ischemic stroke | Simple median | 5 | 0.663 | 0.466 | 0.944 | 0.023 |
|  |  |  | Weighted median | 5 | 0.561 | 0.406 | 0.777 | 0.001 |
|  |  |  | IVW | 5 | 0.588 | 0.453 | 0.763 | **6.55E-05** |
|  |  |  | MR-Egger | 5 | 0.417 | 0.218 | 0.801 | 0.009 |
|  |  |  | (intercept) | 5 | NA | NA | NA | 0.26 |
| PDE5 inhibitors | SBP | CKD | Simple median | 5 | 0.73 | 0.435 | 1.225 | 0.234 |
|  |  |  | Weighted median | 5 | 0.534 | 0.344 | 0.831 | 0.005 |
|  |  |  | IVW | 5 | 0.532 | 0.345 | 0.82 | 0.004 |
|  |  |  | MR-Egger | 5 | 0.236 | 0.118 | 0.47 | 3.96E-05 |
|  |  |  | (intercept) | 5 | NA | NA | NA | 0.01 |
| PDE5 inhibitors | SBP | T2D | Simple median | 5 | 0.951 | 0.643 | 1.407 | 0.802 |
|  |  |  | Weighted median | 5 | 0.808 | 0.613 | 1.066 | 0.132 |
|  |  |  | IVW | 5 | 0.951 | 0.677 | 1.336 | 0.772 |
|  |  |  | MR-Egger | 5 | 0.536 | 0.315 | 0.912 | 0.022 |
|  |  |  | (intercept) | 5 | NA | NA | NA | 0.019 |
| sGC stimulators | SBP | AF | Simple median | 6 | 0.924 | 0.696 | 1.226 | 0.583 |
|  |  |  | Weighted median | 6 | 0.938 | 0.715 | 1.231 | 0.644 |
|  |  |  | IVW | 6 | 0.951 | 0.755 | 1.197 | 0.669 |
|  |  |  | MR-Egger | 6 | 2.152 | 0.352 | 13.159 | 0.407 |
|  |  |  | (intercept) | 6 | NA | NA | NA | 0.373 |
| sGC stimulators | SBP | CAD | Simple median | 6 | 0.319 | 0.239 | 0.426 | 1.01E-14 |
|  |  |  | Weighted median | 6 | 0.325 | 0.243 | 0.434 | 2.77E-14 |
|  |  |  | IVW | 6 | 0.332 | 0.236 | 0.469 | **3.45E-10** |
|  |  |  | MR-Egger | 6 | 1.281 | 0.091 | 18.074 | 0.855 |
|  |  |  | (intercept) | 6 | NA | NA | NA | 0.314 |
| sGC stimulators | SBP | MI | Simple median | 6 | 0.212 | 0.129 | 0.348 | 7.80E-10 |
|  |  |  | Weighted median | 6 | 0.208 | 0.129 | 0.335 | 1.29E-10 |
|  |  |  | IVW | 6 | 0.238 | 0.168 | 0.337 | **5.68E-16** |
|  |  |  | MR-Egger | 6 | 0.378 | 0.024 | 5.843 | 0.486 |
|  |  |  | (intercept) | 6 | NA | NA | NA | 0.739 |
| sGC stimulators | SBP | HF | Simple median | 6 | 1.131 | 0.794 | 1.611 | 0.495 |
|  |  |  | Weighted median | 6 | 1.002 | 0.708 | 1.418 | 0.99 |
|  |  |  | IVW | 6 | 0.987 | 0.676 | 1.442 | 0.947 |
|  |  |  | MR-Egger | 6 | 18.748 | 2.222 | 158.217 | 0.007 |
|  |  |  | (intercept) | 6 | NA | NA | NA | 0.006 |
| sGC stimulators | SBP | Ischemic stroke | Simple median | 6 | 0.626 | 0.458 | 0.857 | 0.003 |
|  |  |  | Weighted median | 6 | 0.629 | 0.463 | 0.854 | 0.003 |
|  |  |  | IVW | 6 | 0.686 | 0.54 | 0.872 | 0.002 |
|  |  |  | MR-Egger | 6 | 2.272 | 0.341 | 15.137 | 0.396 |
|  |  |  | (intercept) | 6 | NA | NA | NA | 0.212 |
| sGC stimulators | SBP | CKD | Simple median | 6 | 0.567 | 0.373 | 0.86 | 0.008 |
|  |  |  | Weighted median | 6 | 0.585 | 0.396 | 0.865 | 0.007 |
|  |  |  | IVW | 6 | 0.55 | 0.398 | 0.761 | **0.0003** |
|  |  |  | MR-Egger | 6 | 0.618 | 0.045 | 8.506 | 0.719 |
|  |  |  | (intercept) | 6 | NA | NA | NA | 0.93 |
| sGC stimulators | SBP | T2D | Simple median | 6 | 0.715 | 0.531 | 0.962 | 0.027 |
|  |  |  | Weighted median | 6 | 0.722 | 0.542 | 0.962 | 0.026 |
|  |  |  | IVW | 6 | 0.793 | 0.628 | 1 | 0.05 |
|  |  |  | MR-Egger | 6 | 2.022 | 0.34 | 12.036 | 0.439 |
|  |  |  | (intercept) | 6 | NA | NA | NA | 0.299 |

Table S8. Effects of genetic instruments on systolic blood pressure and the significant outcomes.

| **Drug.class** | **Target.gene** | **SNP** | **Chr** | **Pos** | **Trait** | **EA** | **NEA** | **EAF** | **Beta** | **SE** | **P** |
| --- | --- | --- | --- | --- | --- | --- | --- | --- | --- | --- | --- |
| AGTi | AGT | rs61751101 | 1 | 230836734 | SBP | A | G | 0.9298 | -0.4036 | 0.0606 | 2.73E-11 |
| AGTi | AGT | rs699 | 1 | 230845794 | SBP | A | G | 0.5928 | -0.3748 | 0.0308 | 5.59E-34 |
| AGTi | AGT | rs61751101 | 1 | 230836734 | CAD | A | G | 0.932 | -0.01239 | 0.009969 | 0.2141 |
| AGTi | AGT | rs699 | 1 | 230845794 | CAD | A | G | 0.5659 | -0.0177 | 0.004976 | 0.000376 |
| AGTi | AGT | rs61751101 | 1 | 230836734 | MI | A | G | 0.9335 | -0.03187 | 0.020746 | 0.124529 |
| AGTi | AGT | rs699 | 1 | 230845794 | MI | A | G | 0.5415 | -0.02812 | 0.010377 | 0.006735 |
| AGTi | AGT | rs61751101 | 1 | 230836734 | Ischemic stroke | A | G | 0.9258 | -0.0282 | 0.0136 | 0.03841 |
| AGTi | AGT | rs699 | 1 | 230845794 | Ischemic stroke | A | G | 0.5812 | -0.0274 | 0.0089 | 0.001944 |
| ERAs | EDNRA | rs13143677 | 4 | 148419040 | SBP | A | G | 0.2933 | 0.2237 | 0.0333 | 1.82E-11 |
| ERAs | EDNRA | rs13143677 | 4 | 148419040 | CAD | A | G | 0.305 | 0.036338 | 0.005359 | 1.19E-11 |
| ERAs | EDNRA | rs13143677 | 4 | 148419040 | MI | A | G | 0.314531 | 0.037429 | 0.011098 | 0.000744 |
| ERAs | EDNRA | rs13143677 | 4 | 148419040 | Ischemic stroke | A | G | 0.2784 | 0.0341 | 0.0081 | 2.76E-05 |
| PDE5 inhibitors | PDE5A | rs4834792 | 4 | 120555696 | SBP | A | T | 0.4796 | 0.1973 | 0.0303 | 7.24E-11 |
| PDE5 inhibitors | NOS3 | rs740956 | 7 | 150598440 | SBP | T | C | 0.5669 | 0.2001 | 0.0305 | 5.21E-11 |
| PDE5 inhibitors | NOS3 | rs3918226 | 7 | 150690176 | SBP | T | C | 0.0811 | 0.664 | 0.0575 | 8.46E-31 |
| PDE5 inhibitors | NOS3 | rs891511 | 7 | 150704843 | SBP | A | G | 0.3335 | -0.3506 | 0.0333 | 6.13E-26 |
| PDE5 inhibitors | NOS3 | rs34581141 | 7 | 150810073 | SBP | A | G | 0.6841 | -0.1879 | 0.0327 | 9.08E-09 |
| PDE5 inhibitors | PDE5A | rs4834792 | 4 | 120555696 | CAD | A | T | 0.4838 | 0.027995 | 0.004891 | 1.04E-08 |
| PDE5 inhibitors | NOS3 | rs740956 | 7 | 150598440 | CAD | T | C | 0.5628 | 0.018707 | 0.004943 | 0.000154 |
| PDE5 inhibitors | NOS3 | rs3918226 | 7 | 150690176 | CAD | T | C | 0.0807 | 0.111949 | 0.009498 | 4.60E-32 |
| PDE5 inhibitors | NOS3 | rs891511 | 7 | 150704843 | CAD | A | G | 0.3425 | -0.01654 | 0.005372 | 0.002083 |
| PDE5 inhibitors | NOS3 | rs34581141 | 7 | 150810073 | CAD | A | G | 0.6913 | -0.00504 | 0.005335 | 0.3446 |
| PDE5 inhibitors | PDE5A | rs4834792 | 4 | 120555696 | Ischemic stroke | A | T | 0.4769 | 0.0081 | 0.0069 | 0.246 |
| PDE5 inhibitors | NOS3 | rs740956 | 7 | 150598440 | Ischemic stroke | T | C | 0.579 | 0.0053 | 0.007 | 0.4541 |
| PDE5 inhibitors | NOS3 | rs3918226 | 7 | 150690176 | Ischemic stroke | T | C | 0.0782 | 0.0452 | 0.0173 | 0.0091 |
| PDE5 inhibitors | NOS3 | rs891511 | 7 | 150704843 | Ischemic stroke | A | G | 0.3645 | -0.0221 | 0.0078 | 0.004603 |
| PDE5 inhibitors | NOS3 | rs34581141 | 7 | 150810073 | Ischemic stroke | A | G | 0.6861 | -0.0062 | 0.0082 | 0.4518 |
| sGC stimulators | GUCY1A3 | rs1483040 | 4 | 156503399 | SBP | C | G | 0.094 | 0.3458 | 0.0527 | 5.22E-11 |
| sGC stimulators | GUCY1A3 | rs990619 | 4 | 156507678 | SBP | C | G | 0.4763 | -0.2974 | 0.0301 | 4.68E-23 |
| sGC stimulators | GUCY1A3 | rs10010626 | 4 | 156519815 | SBP | A | T | 0.0724 | 0.3685 | 0.0627 | 4.12E-09 |
| sGC stimulators | GUCY1A3 | rs28531888 | 4 | 156613960 | SBP | T | C | 0.1985 | -0.2256 | 0.0396 | 1.17E-08 |
| sGC stimulators | GUCY1A3 | rs12643599 | 4 | 156639846 | SBP | A | G | 0.6395 | 0.3134 | 0.0313 | 1.23E-23 |
| sGC stimulators | GUCY1A3 | rs78975989 | 4 | 156718205 | SBP | A | G | 0.1091 | -0.3199 | 0.0501 | 1.67E-10 |
| sGC stimulators | GUCY1A3 | rs1483040 | 4 | 156503399 | CAD | C | G | 0.1075 | 0.057099 | 0.008353 | 8.14E-12 |
| sGC stimulators | GUCY1A3 | rs990619 | 4 | 156507678 | CAD | C | G | 0.4885 | -0.02651 | 0.004936 | 7.81E-08 |
| sGC stimulators | GUCY1A3 | rs10010626 | 4 | 156519815 | CAD | A | T | 0.0694 | 0.009665 | 0.010208 | 0.3437 |
| sGC stimulators | GUCY1A3 | rs28531888 | 4 | 156613960 | CAD | T | C | 0.1914 | -0.03798 | 0.006459 | 4.12E-09 |
| sGC stimulators | GUCY1A3 | rs12643599 | 4 | 156639846 | CAD | A | G | 0.6273 | 0.035657 | 0.005199 | 7.00E-12 |
| sGC stimulators | GUCY1A3 | rs78975989 | 4 | 156718205 | CAD | A | G | 0.1161 | -0.03666 | 0.007998 | 4.55E-06 |
| sGC stimulators | GUCY1A3 | rs1483040 | 4 | 156503399 | MI | C | G | 0.141546 | 0.056653 | 0.015099 | 0.000175 |
| sGC stimulators | GUCY1A3 | rs990619 | 4 | 156507678 | MI | C | G | 0.483027 | -0.03168 | 0.010353 | 0.002209 |
| sGC stimulators | GUCY1A3 | rs10010626 | 4 | 156519815 | MI | A | T | 0.068442 | 0.036191 | 0.021945 | 0.099102 |
| sGC stimulators | GUCY1A3 | rs28531888 | 4 | 156613960 | MI | T | C | 0.186065 | -0.0392 | 0.013691 | 0.004191 |
| sGC stimulators | GUCY1A3 | rs12643599 | 4 | 156639846 | MI | A | G | 0.642464 | 0.053137 | 0.010537 | 4.59E-07 |
| sGC stimulators | GUCY1A3 | rs78975989 | 4 | 156718205 | MI | A | G | 0.116327 | -0.04684 | 0.016042 | 0.003505 |
| sGC stimulators | GUCY1A3 | rs1483040 | 4 | 156503399 | CKD | C | G | 0.09 | 0.0376 | 0.0166 | 0.02316 |
| sGC stimulators | GUCY1A3 | rs990619 | 4 | 156507678 | CKD | C | G | 0.49 | -0.019 | 0.0091 | 0.03649 |
| sGC stimulators | GUCY1A3 | rs10010626 | 4 | 156519815 | CKD | A | T | 0.066 | 0.0135 | 0.0214 | 0.5298 |
| sGC stimulators | GUCY1A3 | rs28531888 | 4 | 156613960 | CKD | T | C | 0.19 | -0.0159 | 0.0127 | 0.2121 |
| sGC stimulators | GUCY1A3 | rs12643599 | 4 | 156639846 | CKD | A | G | 0.62 | 0.0137 | 0.0094 | 0.1436 |
| sGC stimulators | GUCY1A3 | rs78975989 | 4 | 156718205 | CKD | A | G | 0.12 | -0.0159 | 0.0154 | 0.3003 |

Table S9. Sensitivity analysis for sGC stimulation and coronary artery disease risk after removing a SNP

| **outcome** | **exposure** | **method** | **nsnp** | **OR_per10mmHg** | **lo_ci** | **up_ci** | **p-value** |
| --- | --- | --- | --- | --- | --- | --- | --- |
| CAD | SBP | Inverse variance weighted | 5 | 0.302969 | 0.227836 | 0.402878 | 2.17E-16 |
| CAD | SBP | Weighted median | 5 | 0.325578 | 0.242323 | 0.437437 | 9.53E-14 |
| CAD | SBP | Simple median | 5 | 0.317865 | 0.223256 | 0.452564 | 2.04E-10 |
| CAD | SBP | MR Egger | 5 | 0.525023 | 0.037747 | 7.302613 | 0.664236 |

Table S10. MR analyses on negative control outcomes, using inverse-variant weighted method.

SBP, systolic blood pressure; nSNPs, number of single nucleotide polymorphisms; beta, effect size per 10 mmHg decrease in SBP; lo_ci, lower bound of 95% confidence interval of beta ; up_ci, upper bound of 95% confidence interval of beta; pval, p-value; AGTi, Angiotensinogen inhibitor; ERAs, endothelin receptor antagonists; PDE5i, phosphodiesterase type 5 inhibitors; sGC; soluble guanylate cyclase.

| **Drug class** | **nSNP** | **exposure** | **outcome** | **beta** | **lo_ci** | **up_ci** | **pval** |
| --- | --- | --- | --- | --- | --- | --- | --- |
| AGTi | 2 | SBP | low hand grip strength | -0.014 | -0.048 | 0.02 | 0.425 |
| AGTi | 2 | SBP | heel bone mineral density | 0.009 | 0.000 | 0.017 | 0.053 |
| AGTi | 2 | SBP | myopia | 0.008 | -0.028 | 0.045 | 0.666 |
| AGTi | 2 | SBP | Parkinson disease | -0.063 | -0.144 | 0.017 | 0.123 |
| PDE5i | 5 | SBP | low hand grip strength | 0.009 | -0.015 | 0.034 | 0.457 |
| PDE5i | 5 | SBP | heel bone mineral density | 0.013 | 0.003 | 0.022 | 0.01 |
| PDE5i | 5 | SBP | myopia | -0.031 | -0.057 | -0.004 | 0.022 |
| PDE5i | 5 | SBP | Parkinson disease | 0.038 | -0.023 | 0.1 | 0.223 |
| ERAs | 1 | SBP | low hand grip strength | -0.06 | -0.131 | 0.011 | 0.098 |
| ERAs | 1 | SBP | heel bone mineral density | 0.02 | 0.002 | 0.038 | 0.029 |
| ERAs | 1 | SBP | myopia | 0.031 | -0.044 | 0.107 | 0.416 |
| ERAs | 1 | SBP | Parkinson disease | -0.111 | -0.298 | 0.077 | 0.247 |
| sGC stimulators | 6 | SBP | low hand grip strength | -0.012 | -0.039 | 0.015 | 0.38 |
| sGC stimulators | 6 | SBP | heel bone mineral density | 0.009 | 0.003 | 0.016 | 0.01 |
| sGC stimulators | 6 | SBP | myopia | -0.007 | -0.034 | 0.02 | 0.618 |
| sGC stimulators | 6 | SBP | Parkinson disease | -0.05 | -0.124 | 0.023 | 0.18 |

Table S11. MR analysis results for genetically proxied drug classes on the main outcome, using blood pressure estimates from UKB GWAS.

SBP, systolic blood pressure; CAD indicates coronary artery disease; MI, myocardial infarction; AF, atrial fibrillation; HF, heart failure; CKD, chronic kidney disease; T2D, type 2 diabetes; IVW, inverse-variant weighted method; nSNPs, number of single nucleotide polymorphisms; beta, effect size per 1 SD increase in SBP; lo_ci, lower bound of 95% confidence interval of beta ; up_ci, upper bound of 95% confidence interval of beta; pval, p-value; AGTi, Angiotensinogen inhibitor; ERAs, endothelin receptor antagonists; PDE5 inhibitors, phosphodiesterase type 5; sGC; soluble guanylate cyclase.

| **Drug class** | **nSNPs** | **exposure** | **outcome** | **beta** | **lo_ci** | **up_ci** | **pval** |
| --- | --- | --- | --- | --- | --- | --- | --- |
| AGTi | 2 | SBP | AF | 0.228 | -0.485 | 0.94 | 0.532 |
|  | 2 | SBP | CAD | 1.045 | 0.527 | 1.563 | 7.73E-05 |
|  | 2 | SBP | MI | 1.764 | 0.717 | 2.811 | 0.0009 |
|  | 2 | SBP | HF | -0.12 | -0.973 | 0.733 | 0.783 |
|  | 2 | SBP | Ischemic stroke | 1.155 | 0.397 | 1.913 | 0.003 |
|  | 2 | SBP | CKD | -0.019 | -1.002 | 0.965 | 0.97 |
|  | 2 | SBP | T2D | -0.371 | -1.057 | 0.315 | 0.289 |
| ERAs | 1 | SBP | AF | 1.516 | 0.431 | 2.6 | 0.006 |
|  | 1 | SBP | CAD | 2.754 | 1.958 | 3.55 | 1.19E-11 |
|  | 1 | SBP | MI | 2.837 | 1.188 | 4.485 | 0.001 |
|  | 1 | SBP | HF | 0.637 | -0.656 | 1.929 | 0.334 |
|  | 1 | SBP | Ischemic stroke | 2.584 | 1.381 | 3.787 | 2.56E-05 |
|  | 1 | SBP | CKD | -0.356 | -1.931 | 1.218 | 0.657 |
|  | 1 | SBP | T2D | 0.394 | -0.661 | 1.449 | 0.464 |
| PDE5 inhibitors | 5 | SBP | AF | 0.663 | 0.135 | 1.191 | 0.014 |
|  | 5 | SBP | CAD | 2.218 | 0.525 | 3.912 | 0.01 |
|  | 5 | SBP | MI | 2.009 | 0.359 | 3.66 | 0.017 |
|  | 5 | SBP | HF | 0.858 | -0.18 | 1.896 | 0.105 |
|  | 5 | SBP | Ischemic stroke | 1.26 | 0.671 | 1.85 | 2.76E-05 |
|  | 5 | SBP | CKD | 1.434 | 0.211 | 2.658 | 0.022 |
|  | 5 | SBP | T2D | 0.047 | -0.76 | 0.854 | 0.909 |
| sGC stimulators | 6 | SBP | AF | 0.118 | -0.352 | 0.589 | 0.622 |
|  | 6 | SBP | CAD | 2.102 | 1.181 | 3.023 | 7.75E-06 |
|  | 6 | SBP | MI | 2.758 | 1.825 | 3.69 | 6.90E-09 |
|  | 6 | SBP | HF | 0.044 | -0.685 | 0.774 | 0.905 |
|  | 6 | SBP | Ischemic stroke | 0.725 | 0.17 | 1.279 | 0.01 |
|  | 6 | SBP | CKD | 1.168 | 0.508 | 1.829 | 0.001 |
|  | 6 | SBP | T2D | 0.382 | -0.143 | 0.907 | 0.154 |

Table S12. SMR associations of antihypertensive target gene-expression levels with systolic blood pressure

b_SMR_, SMR effect size per 1 SD increase in gene expression; se_SMR_, standard error; A1, effect allele; A2, non-effect allele, Freq, frequency of A1.

| **Drug class** | **Gene** | **Outcome** | **Tissue** | **Top SNP** | **Chr** | **Pos_build37** | **A1** | **A2** | **Freq** | **b_SMR_** | **se_SMR_** | **P_SMR_** |
| --- | --- | --- | --- | --- | --- | --- | --- | --- | --- | --- | --- | --- |
| AGTi | *AGT* | SBP | Adipose Subcutaneous | rs5050 | 1 | 230849886 | G | T | 0.178 | 0.126 | 0.043 | 0.004 |
| AGTi | *AGT* | SBP | Adipose Visceral Omentum | rs5050 | 1 | 230849886 | G | T | 0.178 | 0.302 | 0.114 | 0.008 |
| AGTi | *AGT* | SBP | Adrenal Gland | rs5050 | 1 | 230849886 | G | T | 0.178 | 0.168 | 0.059 | 0.004 |
| AGTi | *AGT* | SBP | Artery tibial | rs1977413 | 1 | 230852533 | A | G | 0.129 | -0.46 | 0.094 | 9.22E-07 |
| AGTi | *AGT* | SBP | Brain Anterior cingulate cortex BA24 | rs10864768 | 1 | 230825447 | C | T | 0.211 | -0.471 | 0.133 | 0.0004 |
| AGTi | *AGT* | SBP | Brain Caudate basal ganglia | rs3789649 | 1 | 230816460 | T | C | 0.211 | -0.336 | 0.087 | 0.0001 |
| AGTi | *AGT* | SBP | Brain Cerebellar Hemisphere | rs5051 | 1 | 230849872 | T | C | 0.413 | -0.467 | 0.056 | 1.15E-16 |
| AGTi | *AGT* | SBP | Brain Cerebellum | rs2493134 | 1 | 230849359 | C | T | 0.411 | -0.472 | 0.054 | 4.47E-18 |
| AGTi | *AGT* | SBP | Brain Cortex | rs10864769 | 1 | 230833927 | G | A | 0.209 | -0.386 | 0.102 | 0.0002 |
| AGTi | *AGT* | SBP | Brain Frontal Cortex BA9 | rs3789649 | 1 | 230816460 | T | C | 0.211 | -0.595 | 0.166 | 0.0003 |
| AGTi | *AGT* | SBP | Brain Nucleus accumbens basal ganglia | rs10864768 | 1 | 230825447 | C | T | 0.211 | -0.347 | 0.093 | 0.0002 |
| AGTi | *AGT* | SBP | Brain Putamen basal ganglia | rs3789649 | 1 | 230816460 | T | C | 0.211 | -0.618 | 0.172 | 0.0003 |
| AGTi | *AGT* | SBP | Muscle Skeletal | rs5050 | 1 | 230849886 | G | T | 0.178 | 0.264 | 0.093 | 0.005 |
| AGTi | *AGT* | SBP | Pancreas | rs5050 | 1 | 230849886 | G | T | 0.178 | 0.261 | 0.097 | 0.007 |
| sGC stimulator | *GUCY1A2* | SBP | Pancreas | rs10895931 | 11 | 106064055 | A | G | 0.776 | -0.196 | 0.06 | 0.001 |
| sGC stimulator | *GUCY1A3* | SBP | Artery aorta | rs17033041 | 4 | 156391307 | G | A | 0.186 | -0.882 | 0.128 | 6.27E-12 |
| sGC stimulator | *GUCY1A3* | SBP | Artery tibial | rs72687508 | 4 | 156628921 | G | A | 0.234 | -2.341 | 0.493 | 2.02E-06 |
| sGC stimulator | *GUCY1A3* | SBP | Heart Atrial Appendage | rs72679986 | 4 | 156469749 | A | T | 0.158 | 0.679 | 0.187 | 0.0003 |
| sGC stimulator | *GUCY1A3* | SBP | Thyroid | rs4690974 | 4 | 156393641 | C | T | 0.508 | -2.174 | 0.354 | 7.93E-10 |
| PDE5i | *PDE5A* | SBP | Artery aorta | rs1393966 | 4 | 120798857 | C | T | 0.538 | 0.619 | 0.202 | 0.002 |
| PDE5i | *PDE5A* | SBP | Thyroid | rs1987179 | 4 | 120519934 | C | T | 0.787 | -0.923 | 0.257 | 0.0003 |

Table S13. SMR associations of antihypertensive target gene-expression with CKM outcomes

b_SMR_, SMR effect size per 1 SD increase in gene expression; se_SMR_, standard error; A1, effect allele; A2, non-effect allele, Freq, frequency of A1.

| **Drug class** | **Gene** | **Outcome** | **Tissue** | **TopSNP** | **Chr** | **A1** | **A2** | **Freq** | **b_SMR_** | **se_SMR_** | **P_SMR_** | **p_HEIDI_** | **nsnp_HEIDI_** |
| --- | --- | --- | --- | --- | --- | --- | --- | --- | --- | --- | --- | --- | --- |
| AGTi | *AGT* | CAD | BC | rs2493134 | 1 | C | T | 0.411 | -0.023 | 0.007 | 0.001 | 0.009 | 20 |
| AGTi | *AGT* | CAD | BCH | rs5051 | 1 | T | C | 0.413 | -0.022 | 0.007 | 0.001 | 0.057 | 20 |
| AGTi | *AGT* | MI | BC | rs2493134 | 1 | C | T | 0.411 | -0.037 | 0.013 | 0.006 | 0.009 | 20 |
| AGTi | *AGT* | MI | BCH | rs5051 | 1 | T | C | 0.413 | -0.04 | 0.014 | 0.004 | 0.009 | 20 |
| AGTi | *AGT* | AF | BCH | rs5051 | 1 | T | C | 0.413 | -0.02 | 0.009 | 0.026 | 0.101 | 20 |
| AGTi | *AGT* | Ischemic stroke | BC | rs2493134 | 1 | C | T | 0.411 | -0.023 | 0.009 | 0.012 | 0.06 | 20 |
| AGTi | *AGT* | Ischemic stroke | BCH | rs5051 | 1 | T | C | 0.413 | -0.038 | 0.012 | 0.001 | 0.001 | 20 |
| AGTi | *AGT* | T2D | ASc | rs5050 | 1 | G | T | 0.178 | 0.018 | 0.009 | 0.048 | 0.646 | 20 |
| sGC stimulators | *GUCY1A3* | CAD | AAo | rs17033041 | 4 | G | A | 0.186 | -0.056 | 0.016 | 0.0003 | 0.001 | 20 |
| sGC stimulators | *GUCY1A3* | CAD | ATib | rs72687508 | 4 | G | A | 0.234 | -0.404 | 0.084 | 1.74E-06 | 0.664 | 20 |
| sGC stimulators | *GUCY1A3* | CAD | HAA | rs72679986 | 4 | A | T | 0.158 | 0.06 | 0.026 | 0.019 | 2.94E-05 | 11 |
| sGC stimulators | *GUCY1A3* | CAD | Thy | rs4690974 | 4 | C | T | 0.508 | -0.173 | 0.038 | 6.13E-06 | 0.0001 | 15 |
| sGC stimulators | *GUCY1A3* | MI | ATib | rs72687508 | 4 | G | A | 0.234 | -0.473 | 0.123 | 0.0001 | 0.873 | 20 |
| sGC stimulators | *GUCY1A3* | MI | HAA | rs72679986 | 4 | A | T | 0.158 | 0.108 | 0.053 | 0.043 | 0.001 | 11 |
| sGC stimulators | *GUCY1A3* | MI | Thy | rs4690974 | 4 | C | T | 0.508 | -0.2 | 0.069 | 0.004 | 0.031 | 15 |
| sGC stimulators | *GUCY1A3* | HF | AAo | rs17033041 | 4 | G | A | 0.186 | -0.052 | 0.024 | 0.028 | 0.555 | 20 |
| sGC stimulators | *GUCY1A3* | Ischemic stroke | ATib | rs72687508 | 4 | G | A | 0.234 | -0.168 | 0.066 | 0.011 | 0.481 | 20 |
| sGC stimulators | *GUCY1A3* | CKD | AAo | rs17033041 | 4 | G | A | 0.186 | -0.061 | 0.029 | 0.039 | 0.237 | 20 |
| sGC stimulators | *GUCY1A3* | CKD | ATib | rs72687508 | 4 | G | A | 0.234 | -0.189 | 0.09 | 0.036 | 0.514 | 20 |
| sGC stimulators | *GUCY1A3* | T2D | AAo | rs17033041 | 4 | G | A | 0.186 | -0.039 | 0.019 | 0.041 | 0.796 | 20 |
| PDE5 inhibitors | *PDE5A* | CAD | AAo | rs1393966 | 4 | C | T | 0.538 | 0.171 | 0.039 | 9.11E-06 | 0.338 | 20 |
| PDE5 inhibitors | *PDE5A* | MI | AAo | rs1393966 | 4 | C | T | 0.538 | 0.141 | 0.064 | 0.027 | 0.306 | 20 |
| PDE5 inhibitors | *PDE5A* | T2D | AAo | rs1393966 | 4 | C | T | 0.538 | 0.105 | 0.041 | 0.01 | 0.907 | 20 |

Table S14. Colocalisation results.

nSNP, number of SNPs; PP, posterior probability; CAD, coronary artery disease, MI, myocardial infarction.

| **nSNP** | **PP.H0** | **PP.H1** | **PP.H2** | **PP.H3** | **PP.H4** | **Trait 1** | **Trait 2** |
| --- | --- | --- | --- | --- | --- | --- | --- |
| 7986 | <0.00001 | <0.00001 | <0.00001 | 0.0604 | 0.93933 | *GUCY1A3* (in artery tibial) | CAD |
| 6737 | <0.00001 | 0.00017 | 0.0002 | 0.04692 | 0.95268 | *GUCY1A3* (in artery tibial) | MI |
| 7241 | <0.00001 | <0.00001 | <0.00001 | 0.52156 | 0.47844 | *PDE5A* (in artery aorta) | CAD |

Supplementary References

1. Evangelou E, Warren HR, Mosen-Ansorena D, Mifsud B, Pazoki R, Gao H, et al. Genetic analysis of over 1 million people identifies 535 new loci associated with blood pressure traits. Nature Genetics. 2018;50(10):1412-25.

2. Gill D, Georgakis MK, Koskeridis F, Jiang L, Feng Q, Wei WQ, et al. Use of Genetic Variants Related to Antihypertensive Drugs to Inform on Efficacy and Side Effects. Circulation. 2019;140(4):270-9.

3. Palmer TM, Lawlor DA, Harbord RM, Sheehan NA, Tobias JH, Timpson NJ, et al. Using multiple genetic variants as instrumental variables for modifiable risk factors. Statistical Methods in Medical Research. 2011;21(3):223-42.

4. Consortium GT. The GTEx Consortium atlas of genetic regulatory effects across human tissues. Science. 2020;369(6509):1318-30.

5. Bowden J, Davey Smith G, Haycock PC, Burgess S. Consistent Estimation in Mendelian Randomization with Some Invalid Instruments Using a Weighted Median Estimator. Genet Epidemiol. 2016;40(4):304-14.

6. Bowden J, Davey Smith G, Burgess S. Mendelian randomization with invalid instruments: effect estimation and bias detection through Egger regression. Int J Epidemiol. 2015;44(2):512-25.

7. Zhu Z, Zhang F, Hu H, Bakshi A, Robinson MR, Powell JE, et al. Integration of summary data from GWAS and eQTL studies predicts complex trait gene targets. Nat Genet. 2016;48(5):481-7.

8. Chauquet S, Zhu Z, O'Donovan MC, Walters JTR, Wray NR, Shah S. Association of Antihypertensive Drug Target Genes With Psychiatric Disorders: A Mendelian Randomization Study. JAMA Psychiatry. 2021;78(6):623-31.

9. Jones G, Trajanoska K, Santanasto AJ, Stringa N, Kuo CL, Atkins JL, et al. Genome-wide meta-analysis of muscle weakness identifies 15 susceptibility loci in older men and women. Nat Commun. 2021;12(1):654.

10. Jiang L, Zheng Z, Fang H, Yang J. A generalized linear mixed model association tool for biobank-scale data. Nat Genet. 2021;53(11):1616-21.

11. Nalls MA, Blauwendraat C, Vallerga CL, Heilbron K, Bandres-Ciga S, Chang D, et al. Identification of novel risk loci, causal insights, and heritable risk for Parkinson's disease: a meta-analysis of genome-wide association studies. Lancet Neurol. 2019;18(12):1091-102.

12. Loh PR, Kichaev G, Gazal S, Schoech AP, Price AL. Mixed-model association for biobank-scale datasets. Nat Genet. 2018;50(7):906-8.
